# Emotional and Behavioural Problems in Children With Functional Gastrointestinal Disorders

**DOI:** 10.1111/apa.70521

**Published:** 2026-03-19

**Authors:** Sebastian Weber, Anna Geser, Eva Unternaehrer, Margarete Bolten, Corinne Légeret

**Affiliations:** ^1^ Faculty of Medicine University of Basel Basel Switzerland; ^2^ Child and Adolescent Psychiatric Research Department, University Psychiatric Clinics Basel University of Basel Basel Switzerland; ^3^ Luzerner Psychiatrie AG Child & Adolescent Psychiatry at Children's Hospital Central Switzerland (KidZ) Lucerne Switzerland; ^4^ Department of Gastroenterology University Children's Hospital Basel (UKBB) Basel Switzerland

**Keywords:** child mental health, disorders of gut–brain interaction, emotional and behavioural problems, functional gastrointestinal disorders, psychosocial factors

## Abstract

**Aim:**

Functional gastrointestinal disorders (FGIDs) are common in childhood, but psychological correlates remain poorly understood. We compared emotional and behavioural functioning in children with FGIDs with somatic and healthy controls.

**Methods:**

Parents of children aged 2–12 years were recruited at the University Children's Hospital Basel (February 2024–February 2025). Children meeting Rome IV criteria formed the *Functional Group*; those with somatic gastrointestinal diseases and healthy peers served as *Somatic* and *Healthy Controls*. Parents completed the Strengths and Difficulties Questionnaire (SDQ) and Patient Health Questionnaire (PHQ‐4). Group differences were tested using non‐parametric models and adjusted negative binomial regression.

**Results:**

Data from 218 children were analysed (*Functional n* = 66, *Somatic n* = 62, *Healthy n* = 90). SDQ total difficulties were markedly higher in FGIDs than in both control groups (*p* < 0.001). Differences were most pronounced for emotional and hyperactivity problems. Using age‐adjusted SDQ total difficulties cut‐offs, elevated scores occurred in 45.5% of children with FGIDs, compared with 8.1% in somatic and 10.0% in healthy peers. Parental psychological distress and separation predicted higher difficulties.

**Conclusion:**

Emotional and behavioural difficulties in FGIDs exceed those observed in somatic gastrointestinal diseases, indicating a particularly high psychosocial burden. These findings highlight the relevance of psychological aspects within integrated paediatric gastroenterology care.

## Key Notes

- Almost half of children with functional gastrointestinal disorders show clinically relevant emotional and behavioural difficulties.
- The psychosocial burden is higher than in children with organic gastrointestinal disease or healthy peers.
- These findings support the importance of integrated, multidisciplinary care addressing psychological and family‐level factors in children with functional gastrointestinal disorders.

**Abbreviations**
ADHD: Attention‐Deficit/Hyperactivity Disorder
AIC: Akaike Information Criterion
CI: confidence interval
DGBI: disorders of gut–brain interaction
FG: functional group
FGIDs: functional gastrointestinal disorders
GI: gastrointestinal
HC: healthy control
IRR: incidence rate ratio
KW: Kruskal–Wallis
PHQ‐4: Patient Health Questionnaire‐4
SC: somatic control
SD: standard deviation
SDQ: Strengths and Difficulties Questionnaire
VIF: variance inflation factor

## 1 Introduction

Functional gastrointestinal disorders (FGIDs) are among the most common chronic conditions in childhood, affecting approximately 22%–25% of children worldwide [1, 2, 3, 4, 5], with no consistent sex differences reported [5]. FGIDs represent an umbrella term for recurrent gastrointestinal symptoms without identifiable organic pathology. These disorders are conceptualised within a biopsychosocial framework, in which genetic, sociocultural and early‐life factors shape vulnerability to altered gastrointestinal functioning, including motility changes, visceral hypersensitivity, immune dysfunction and microbiota disturbances [6]. Psychological stress and emotional states are further recognised as important modulator of gut function via autonomic and neuroendocrine pathways [7], although the directionality remains unclear [8, 9]. While paediatric Rome IV criteria retain the term FGIDs, the adult Rome IV classification has shifted towards the concept of disorders of gut–brain interaction (DGBI), reflecting growing recognition of bidirectional communication between the central nervous system and the gastrointestinal tract [1, 6, 10]. This framework provides a theoretical basis for examining psychological and behavioural dimensions in the present study.

Daily stressors and adverse life events (e.g., abuse, parental separation) are consistently linked to symptom onset and severity [6, 8, 11, 12]. Children with FGIDs report reduced quality of life [5, 13] and elevated rates of mental health difficulties, including symptoms of anxiety (60%–80%) and depression (38%–40%) [12, 14, 15]. Notably, over 40% of anxious children meet FGID criteria compared with 6% of peers [16]. Emotional dysregulation (58%) and Attention‐Deficit/Hyperactivity Disorder (ADHD; 24%) are also frequently observed in children with FGIDs [15]. Despite increasing recognition of these comorbidities, no previous study has directly compared emotional and behavioural profiles between children with functional and organic gastrointestinal (GI) disorders, limiting insight into whether these difficulties may be more pronounced in FGIDs or reflect psychosocial distress common to chronic illness in general.

This study aimed to examine emotional and behavioural problems in children with FGIDs using the Strengths and Difficulties Questionnaire (SDQ) [17]. We hypothesised that children with FGIDs would exhibit higher SDQ scores than both healthy peers and children with organic GI disease.

## 2 Methods

### 2.1 Study Design and Participants

We conducted a cross‐sectional study at the University Children's Hospital Basel (UKBB), recruiting parents of children aged 2–12 years between February 2024 and February 2025.

Parents of children meeting the Rome IV criteria [5] for FGIDs and showing no abnormal findings in standard laboratory investigations (including coeliac screening, allergy testing, thyroid‐stimulating hormone and erythrocyte sedimentation rate) or endoscopic biopsy were invited to participate in the *functional group* (*FG*). Exclusion criteria included any other chronic condition or regular medication use.

The *somatic control group* (*SC*) comprised parents of children with confirmed organic gastrointestinal (GI) diseases, defined as: (i) moderate to severe chronic inflammatory bowel disease treated with biologics, (ii) dependence on enteral (via percutaneous endoscopic gastrostomy or jejunal tube) or parenteral nutrition or (iii) chronic liver disease requiring immunosuppressive therapy. Children with any coexisting FGIDs or other chronic conditions were excluded. Regular medication use not related to the underlying organic GI disease was also an exclusion criterion.

FG and SC groups were recruited during paediatric gastroenterology outpatient visits or inpatient stays.

The *healthy control group* (*HC*) comprised parents of children without any chronic medical condition. Recruitment occurred via (i) a school and daycare centre in Basel (*n* = 61) and (ii) the University Children's Hospital Basel outpatient clinic (*n* = 29), where inclusion was restricted to children presenting with minor acute traumatic conditions. To ensure a healthy comparison group, parents completed a screening questionnaire at enrolment. Children with any chronic health condition, regular medication intake or regular medical follow‐up outside routine preventive care were excluded. As a second control step, all children with elevated scores on SDQ item 3 (recurrent abdominal pain, headaches or nausea) were removed.

Questionnaires were available in both digital and paper formats. Online completion was encouraged to ensure complete data submission; only datasets with fully completed SDQs were included in the analysis.

### 2.2 Variables

The Rome IV guideline distinguishes FGIDs for infants/toddlers (≤ 4 years) [18] and children/adolescents (4–18 years) [10], subdividing paediatric FGIDs into functional nausea and vomiting, abdominal pain and defecation disorders [10]. To maximise sample size. FGID subtypes were not analysed separately. Parents reported demographic characteristics of the child and family, including age, sex, parental separation, educational level and employment status of both parents.

The primary outcome was the SDQ total difficulties score (range 0–40), and secondary outcomes were the five SDQ subscales (5 items per subscale, range 0–10): emotional problems, conduct problems, hyperactivity, peer problems and prosocial behaviour. On the SDQ, parents rated statements on a Likert scale from 0 (not true) to 2 (certainly true). The total difficulties score was calculated as the sum of all subscales except the prosocial scale. For clinical interpretation, established parent‐report cut‐offs were applied to classify total difficulties as normal (0–13), borderline (14–16) and abnormal (17–40) for children aged 4–17 years, with age‐adjusted cut‐offs applied for younger children. Borderline and abnormal scores indicate an increased risk for clinically relevant emotional or behavioural difficulties. Higher scores indicate greater difficulties, except for the prosocial subscales, where higher values reflect better social functioning [19, 20]. Parents additionally completed the Patient Health Questionnaire (PHQ‐4) to assess parental anxiety and depression symptoms (total score 0–12) [21, 22].

### 2.3 Bias

To minimise selection bias, the study included both somatic and healthy control groups. Potential reporting bias related to parental mood was addressed by adjusting analyses for parental PHQ‐4.

### 2.4 Statistical Methods

Descriptive statistics summarised demographic variables and SDQ scores. Group comparisons were performed using Chi‐squared tests for categorical variables and Kruskal–Wallis tests with Dunn's post hoc analyses for ordinal or non‐normally distributed continuous variables. Associations were examined using Spearman correlations for ordinal or continuous variables and point‐biserial correlations for dichotomous variables. SDQ total and subscale scores represent sums of item ratings and were analysed as discrete, bounded count outcomes. As score distributions showed overdispersion, with variances exceeding the means, negative binomial regression was applied. Covariates were defined a priori based on clinical relevance and variables showing significant group differences or significant associations with SDQ outcomes, including child age, sex, parental separation, parental education and parental psychological distress. Multicollinearity was checked using VIF. Sensitivity analyses comprised exclusion of residual outliers and multiple imputation for missing data. For the primary outcome, additional interaction testing between study groups and key demographic variables as well as age‐stratified and overlapping age‐range analyses were performed. All regression analyses were based on raw SDQ scores. Model estimates are reported as IRR with 95% CI and SE. Using the FG as reference, IRR values < 1 indicate lower SDQ scores in comparison groups. Model performance was assessed with AIC and Nagelkerke's *R* ^2^. Statistical significance was defined as *p* < 0.05 (two‐tailed).

All analyses were performed using R (version 4.3.2) within RStudio (version 2023.12.1; Posit Software, PBC, Boston, MA, USA). Data management, visualisation and modelling were conducted using tidyverse, dplyr, ggplot2, easystats. Additional packages are listed in Appendix S1.

### 2.5 Ethics

Given the anonymous study design, the local Ethics Committee of Northwestern and Central Switzerland (EKNZ) waived the requirement for written informed consent (EKNZ Req‐2024‐00184). Participation was considered as implied consent.

## 3 Results

### 3.1 Sample Characteristics

Of 259 initiated questionnaires, 41 were excluded (20 incomplete and 21 not meeting HC screening criteria), resulting in a final sample of 218 parents (FG *n* = 66; SC *n* = 62; HC *n* = 90) (Table 1). Most questionnaires were completed by mothers (74.5%). The mean child age across all groups was 7.31 years (SD = 2.91) with no significant sex differences between groups. Child age differed significantly (*p* = 0.007), with FG children being younger than those in the HC. Parental separation was more frequent in FG compared with the HC group (34.9% vs. 14.4%, post hoc *p* = 0.013), and the educational level of the index parent was highest in the HC group (*p* = 0.003).

**TABLE 1:** Demographic characteristics of the study sample.

	Functional group	Somatic control	Healthy control	*p*	Group difference (*χ* ^2^/KW)
Completed questionnaires, *n* (% of all)	66 (30.3%)	62 (28.4%)	90 (41.3%)		
Filled out by mother, *n* (% per group)	50 (75.8%)	42 (67.7%)	69 (76.7%)	0.47	*χ* ^2^ = 1.510
Child characteristics					
Age, years, mean (SD)	6.56 (2.8)	8.15 (3.0)	7.27 (2.78)	0.007	KW *χ* ^2^ = 9.797
Female, *n* (%)	32 (48.5%)	32 (51.6%)	41 (45.6%)	0.762	*χ* ^2^ = 0.543
Parents separated, *n* (%)	23 (35.4%)	12 (19.4%)	13 (14.4%)	0.007	*χ* ^2^ = 9.991
Index parent					
Age, years, mean (SD)	40.1 (6.4)	41.2 (5.8)	41.9 (5.7)	0.188	KW *χ* ^2^ = 3.340
Educational level^a^				0.003	KW *χ* ^2^ = 11.643
High, *n* (%)	35 (53.8%)	37 (59.7%)	71 (78.9%)		
Intermediate, *n* (%)	25 (38.5%)	24 (38.7%)	16 (17.8%)		
Low, *n* (%)	5 (7.8%)	1 (1.6%)	3 (3.3%)		
Employment status				0.952	KW *χ* ^2^ = 0.097
Fulltime, *n* (%)	19 (28.8%)	15 (24.2%)	25 (27.8%)		
Parttime, *n* (%)	36 (54.5%)	42 (67.7%)	54 (60.0%)		
Currently no paid work^b^, *n* (%)	11 (16.7%)	5 (8.1%)	11 (12.2%)		
Employment percentage, mean (SD)	62.10% (35)	67.70% (28.9)	62.78% (32.6)	0.669	KW *χ* ^2^ = 0.802
Second parent					
Educational level^a^				0.223	KW *χ* ^2^ = 2.999
High, *n* (%)	40 (62.5%)	43 (69.4%)	67 (74.4%)		
Intermediate, *n* (%)	17 (26.6%)	15 (24.2%)	19 (21.2%)		
Low, *n* (%)	4 (6.2%)	4 (6.5%)	3 (3.3%)		
Unknown, *n* (%)	3 (4.7%)	0 (0%)	1 (1.1%)		
Employment status				0.717	KW *χ* ^2^ = 0.664
Fulltime, *n* (%)	34 (51.5%)	35 (56.5%)	47 (52.2%)		
Part‐time, *n* (%)	21 (31.8%)	20 (32.3%)	37 (41.4%)		
Currently no paid work^b^, *n* (%)	8 (12.1%)	6 (9.7%)	4 (4.4%)		
Unknown, *n* (%)	3 (4.5%)	1 (1.6%)	2 (2.2%)		
Employment percentage, mean (SD)	77.90% (34.1)	79.67% (31.5)	83.69% (24.5)	0.972	KW *χ* ^2^ = 0.058

*Note:* Overall missing values per variable (*n*): Filled out by (2), Parents separated (1), Index parent Age (15), Index parent Educational level (1), Second parent Educational level (2). *p*‐values refer to overall group comparisons (KW = Kruskal–Wallis test or *χ* ^2^ test). All detailed post hoc contrasts are available in R Markdown script (Appendix S1).

^a^ Educational level: high (University, University of applied sciences), intermediate (apprenticeship, high school, higher vocational training), low (compulsory education, no completed schooling).

^b^ Currently no paid work defined as: household, student, social insurance, unemployed.

### 3.2 Emotional and Behavioural Outcomes

#### 3.2.1 Psychometric Properties of the SDQ

The SDQ showed acceptable to good internal consistency across all groups (Cronbach's *α* = 0.79 for SDQ total; subscales *α* = 0.70–0.82), comparable to published parent‐report reference data (mean *α* = 0.73; total scores > 0.80). Internal consistencies were slightly lower for the Conduct and Hyperactivity scales, consistent with previous studies [20].

#### 3.2.2 Correlations

Across the full sample, higher SDQ total scores were associated with younger child age (*r* = −0.25, *p* < 0.001) and greater parental psychological distress (PHQ‐4 total; *r* = 0.49, *p* < 0.001). Girls had slightly lower SDQ total scores (*r* = −0.15, *p* = 0.029), and children of separated parents had significantly higher SDQ scores (*r* = 0.27, *p* < 0.001). Whether the questionnaire was completed by the mother or father showed no significant association with SDQ total scores (*r* = −0.09, *p* = 0.20).

#### 3.2.3 Group Differences

**FIGURE 1:**
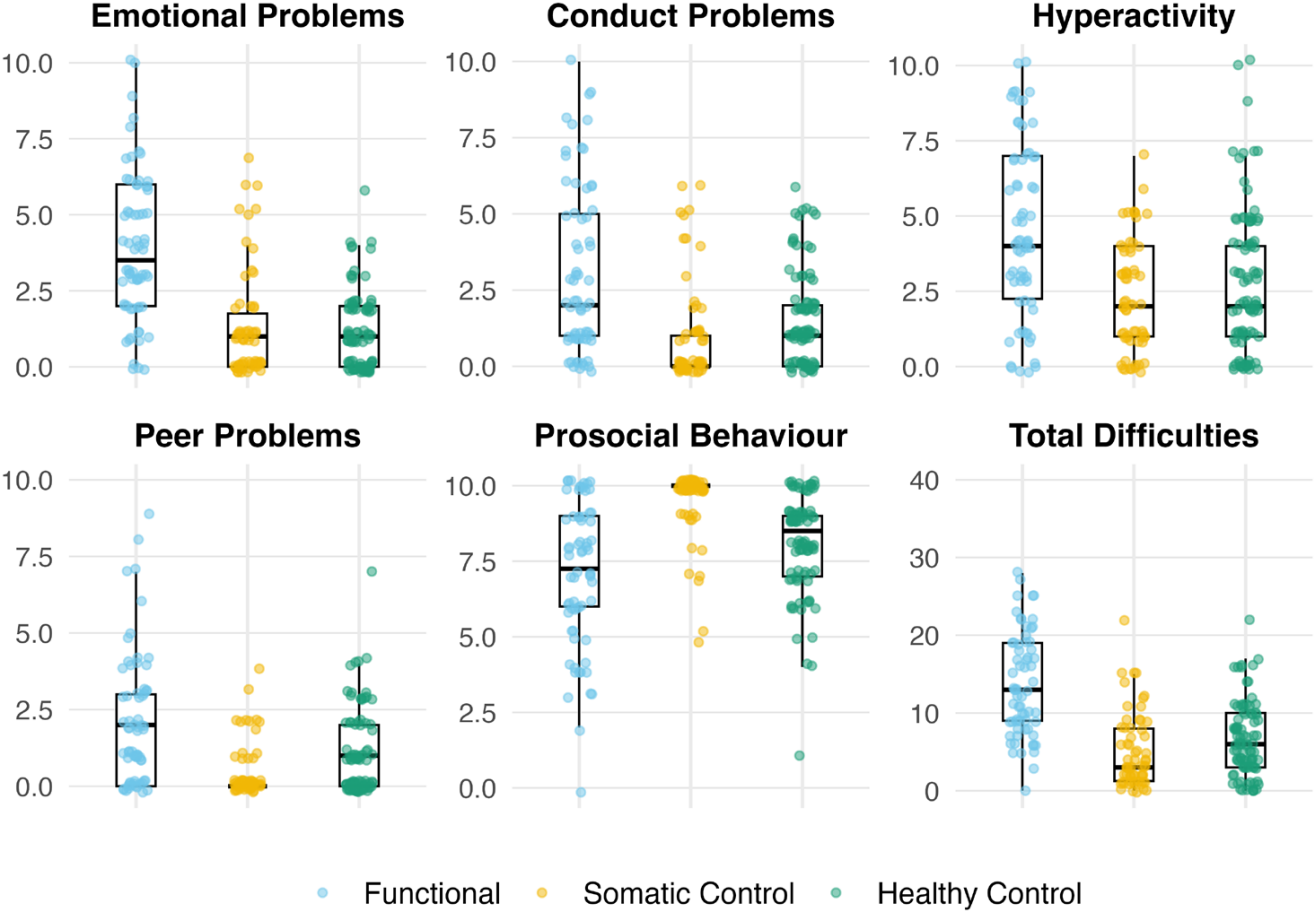
SDQ total difficulty and subscale scores for FG, SC, HC. IRRs < 1 indicate lower SDQ scores relative to the functional group. Points show IRRs; horizontal bars represent 95% confidence intervals. Models adjusted for age, sex, parental separation, parental education and parental psychological distress. SDQ subscale scores range from 0 to 10 and total difficulties from 0 to 40. Boxes represent interquartile ranges with medians (black line); individual dots show participant raw scores.

Descriptive results of SDQ subscales and total score for each group are presented in Table 2. Children with FGIDs showed higher mean scores on all SDQ subscales and on total difficulties score, indicating greater emotional and behavioural difficulties, except on the prosocial scale, where lower scores reflect reduced prosocial behaviour compared with both control groups. The largest effect sizes were found for emotional problems and total difficulties. The distribution of SDQ scores across groups is shown in Figure 1.

**TABLE 2:** Group differences in SDQ total and subscale scores.

SDQ scale	FG	SC	HC	FG–SC, Cliff's Δ	*p*	FG–HC, Cliff's Δ	*p*
Emotional problems	3.92 (2.42)	1.27 (1.78)	1.14 (1.28)	0.65 [0.48–0.77]	< 0.001	0.69 [0.55–0.80]	< 0.001
Conduct problems	3.08 (2.78)	1.00 (1.67)	1.58 (1.56)	0.52 [0.34–0.66]	< 0.001	0.30 [0.12–0.47]	0.006
Hyperactivity	4.48 (2.89)	2.31 (1.81)	2.76 (2.43)	0.44 [0.25–0.60]	< 0.001	0.35 [0.17–0.51]	< 0.001
Peer problems	2.09 (2.12)	0.44 (0.88)	1.04 (1.36)	0.54 [0.37–0.67]	< 0.001	0.31 [0.13–0.47]	0.003
Prosocial behaviour	7.07 (2.40)	9.52 (1.13)	8.12 (1.65)	‐ 0.66 [− 0.77 to −0.50]	< 0.001	−0.25 [−0.42 to −0.06]	0.031
Total difficulties	13.58 (6.55)	5.02 (4.74)	6.52 (4.73)	0.72 [0.57–0.83]	< 0.001	0.62 [0.46–0.73]	< 0.001

*Note:* Values are means (Standard deviations). Cliff's Δ indicates effect size with [95% Confidence interval]. Value < 1 = lower score compared to Functional Group. All *p*‐values Dunn–Bonferroni adjusted (two‐tailed).

Abbreviations: FG, Functional Group; HC, Healthy Control; SC, Somatic Control.

#### 3.2.4 Clinical Cut‐Offs

Using age‐adjusted SDQ cut‐offs indicating risk of clinical impairment, 45.5% of children with FGIDs had an elevated total difficulties score (borderline 9.1%, abnormal 36.4%), compared with 8.1% in the SC (borderline 6.5%, abnormal 1.6%) and 10.0% in the HC (borderline 6.7%, abnormal 3.3%). A graphical and tabular summary of this analysis is provided in Appendix S1.

### 3.3 Primary Outcome Model

**FIGURE 2:**
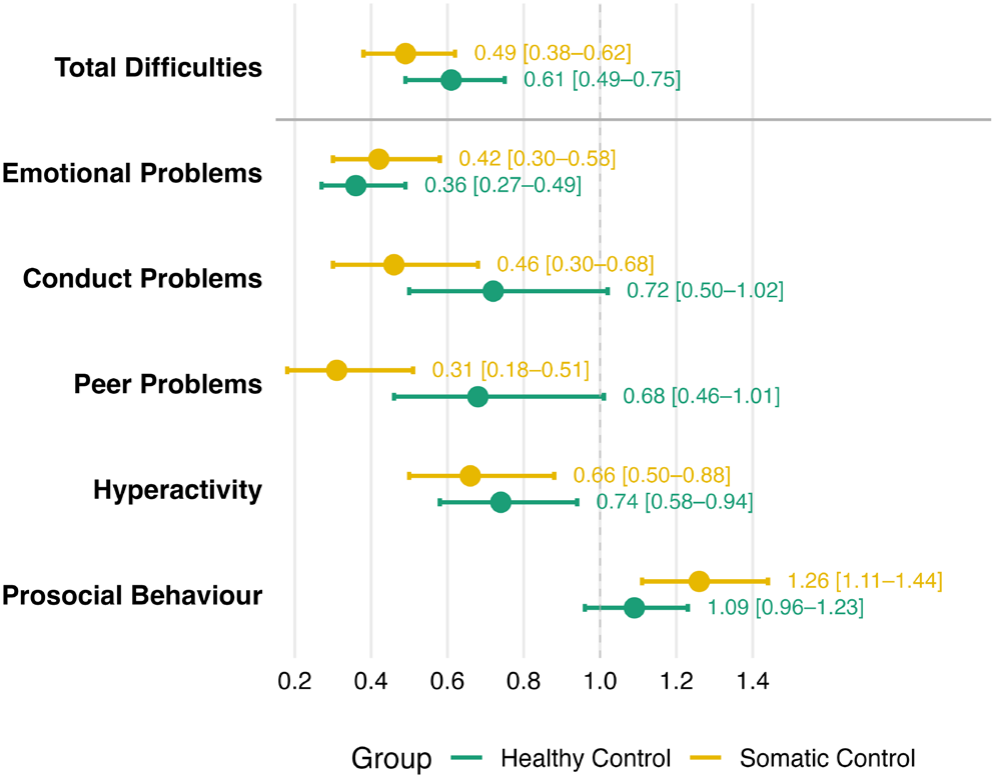
Adjusted incidence rate ratios (IRRs) for SDQ total difficulty and subscale scores (reference: functional group). Values are IRRs with 95% confidence intervals.

Negative binomial regression confirmed that children in both control groups had significantly lower SDQ total difficulty scores compared with the FG. Adjusted IRR with 95% CI are reported in Table 3 and summarised graphically in Figure 2. Among covariates, younger child age, male sex, parental separation and higher parental PHQ‐4 scores were significantly associated with higher SDQ total scores. Overall fit was good (Nagelkerke's *R* ^2^ = 0.57).

**TABLE 3:** Predictors of SDQ total difficulties score (negative binomial regression model).

Parameter	IRR	SE	95% CI	*p*
(Intercept)	10.96	2.06	[7.63, 15.81]	< 0.001
Somatic control	0.49	0.06	[0.38, 0.62]	< 0.001
Healthy control	0.61	0.07	[0.49, 0.75]	< 0.001
Child gender (male)	0.78	0.07	[0.66, 0.93]	0.006
Child age (years)	0.96	0.02	[0.93, 0.99]	0.012
Parents separated	1.24	0.13	[1.00, 1.53]	0.05
Parental education level	1.12	0.09	[0.95, 1.31]	0.171
PHQ‐4 total score	1.12	0.02	[1.07, 1.17]	< 0.001

*Note:* IRR Values < 1 indicate lower SDQ total difficulties relative to the reference group (FG). Model adjusted for all listed covariates.

Abbreviations: CI, Confidence Interval; IRR, Incidence Rate Ratio; SE, Standard Error.

### 3.4 Secondary Outcome Models

Results of the SDQ subscale regressions are summarised in Figure 2. Across all domains except prosocial behaviour, children with FGIDs showed significantly higher adjusted scores than both control groups. The largest group effects were observed for emotional problems and total difficulties, confirming the robustness of group differences across domains. Parental psychological distress (PHQ‐4) emerged as a consistent and independent predictor of higher SDQ subscale scores (all *p* < 0.001). Parental separation was specifically associated with higher conduct problem scores (IRR = 1.45, *p* = 0.032), whereas female sex was linked to lower hyperactivity (IRR = 0.65, *p* < 0.001). For prosocial behaviour, only children in the SC group showed significantly higher scores compared to FG (IRR = 1.26, *p* < 0.001).

Sensitivity analyses, including interaction testing and age‐stratified analyses, showed no evidence of effect modification and did not change interpretation of the results. A fully reproducible R script including all session information and supplementary analyses (e.g., correlations, post hoc tests) is provided in the Appendix S1.

## 4 Discussion

This study demonstrates that children with FGIDs had significantly higher SDQ total difficulties scores than both healthy controls and children with somatic GI disorders, confirming our primary hypothesis. Group differences were most pronounced for internalising symptoms, particularly emotional problems. Using age‐adjusted SDQ cut‐offs, almost half of children with FGIDs scored in the borderline or abnormal range, indicating a markedly increased risk for clinically relevant psychosocial impairment. Importantly, children with FGIDs showed greater difficulties than those with chronic somatic GI disorders, suggesting that these patterns are not solely a consequence of physical illness but may reflect a particularly strong psychosocial component associated with FGIDs.

FGIDs are highly prevalent in children (22%–25%) [1, 2, 3, 4, 5] and substantially impair development, leading to reduced quality of life, increased school absenteeism and higher healthcare utilisation [5, 13]. These impairments often exceed those observed in children with somatic GI disease, underscoring their underestimated public health relevance [13]. Our findings extend prior research by showing that emotional and behavioural problems are closely linked to FGIDs, reinforcing their biopsychosocial conceptualisation. Previous studies have reported strong associations between FGIDs and psychiatric disorders, emphasising their bidirectional pathophysiology, although the causal direction remains unclear [8, 9]. By including children with somatic GI diseases as a comparison group, our findings suggest that emotional and behavioural difficulties in children with FGIDs extend beyond a general response to chronic gastrointestinal illness. These associations remained robust after adjustment for key psychosocial covariates. Together, this pattern raises the hypothesis that, in some children, FGIDs may reflect a somatic expression of broader emotional regulatory vulnerability within a gut‐brain interaction framework.

Interestingly, children in the SC group showed lower SDQ scores than HC in several domains. This counterintuitive finding may reflect selection bias, as families managing chronic somatic disease are often well integrated into medical care and may benefit from structured support. Alternatively, repeated exposure to illness‐related challenges might foster resilience and adaptive coping, mitigating emotional and behavioural difficulties despite ongoing disease burden. Families facing chronic illness often develop flexible organisational patterns, effective communication and problem‐solving skills that promote emotional adjustment and positive parenting [23, 24, 25, 26, 27].

Within the biopsychosocial framework, early‐life, genetic and environmental factors shape gut physiology, while psychological processes contribute to symptom generation via the gut‐brain axis. Relevant mechanisms include stress exposure, somatisation and psychiatric comorbidity such as anxiety, depression and ADHD [9, 15]. Maladaptive coping (e.g., catastrophizing, withdrawal, low self‐efficacy) is linked to poorer outcomes, whereas adaptive strategies (acceptance, positive reframing) mitigate symptoms [28]. Parental psychological distress emerged as a strong correlate of child emotional and behavioural difficulties. Importantly group differences of SDQ scores remained robust after adjustment for parental PHQ‐4 scores, indicating that observed group effects were not solely explained by parental mental health. This finding is consistent with previous literature describing bidirectional associations between parental mental health and child functioning, highlighting the relevance of family‐level mental health within a biopsychosocial framework of paediatric FGIDs [29, 30, 31]. Findings regarding parental separation remain inconsistent. While some studies link divorce to increased FGID risk [29], others report no associations with separation, occupation or educational level [32]. In our sample, separation rates were higher among FG parents, whereas parental occupation and education did not differ.

The SDQ is a validated screening tool capturing distinct domains of child mental health and psychopathology. Elevated emotional scores reflect internalising symptoms such as anxiety or depression, whereas high conduct and hyperactivity scores indicate externalising issues, including attention or behavioural regulation problems [20, 33]. The peer problems subscale generally shows lower reliability but may reflect social withdrawal or peer relationship difficulties, while lower prosocial scores indicate reduced empathy and social engagement [20, 34]. Our findings highlight the predominance of internalising processes in FGIDs, consistent with studies linking emotional difficulties to somatic complaints, whereas behavioural problems show weaker associations [35]. As no Swiss SDQ norms exist, interpretations rely on European reference data, which may limit cultural comparability.

Strengths of this study include the comparison with both somatic and healthy control groups and the use of validated, standardised measures. Some limitations should be acknowledged. Selection bias cannot be fully excluded, as HC were recruited from both school‐ and hospital‐based settings, which may limit generalisability. However, strict exclusion criteria were applied to ensure a well‐defined healthy comparison group. In addition, reasons and frequencies of exclusion were not systematically recorded across all groups. Multiple psychosocial risk factors were more common in FG; despite statistical adjustment, their clustering may introduce residual confounding. Further limitations include modest sample size, reliance on parent‐reported data and the cross‐sectional design, which precludes causal inference. Syndrome‐specific analyses were not feasible. Nevertheless, to our knowledge, this is the first study to directly compare SDQ profiles between children with functional and somatic GI conditions.

Our findings underscore the relevance of psychological factors within an integrated, multidisciplinary approach to paediatric FGIDs, consistent with the bidirectional gut‐brain axis and the need to address both somatic and psychosocial components in clinical care. Such an approach aligns with current recommendations, including cognitive‐behavioural therapy and hypnotherapy [9, 12, 36]. Despite their high burden, FGIDs remain stigmatised and perceived as less severe than organic disorders [9]. Furthermore, awareness of psychosocial issues and the availability of appropriate interventions among paediatricians are often insufficiently implemented [37]. Strengthening physician‐patient communication, validating children's symptoms and incorporating routine mental health assessment into gastroenterological care may improve outcomes and reduce unnecessary diagnostic procedures [6]. Future research should focus on identifying specific psychological mechanisms and resilience factors underlying FGIDs and on developing targeted, family‐based interventions that integrate medical and psychosocial care.

## Author Contributions

**Sebastian Weber:** conceptualization, methodology, data curation, visualization, writing – original draft, investigation, formal analysis. **Anna Geser:** conceptualization, methodology, data curation, investigation, formal analysis, visualization, writing – original draft. **Eva Unternaehrer:** conceptualization, methodology, data curation, supervision, formal analysis, writing – review and editing, visualization, validation. **Margarete Bolten:** conceptualization, methodology, validation, supervision, project administration, resources, writing – review and editing. **Corinne Légeret:** conceptualization, methodology, investigation, validation, supervision, project administration, resources, writing – review and editing.

## Funding

The authors have nothing to report.

## Disclosure

Use of Artificial Intelligence: Artificial intelligence tools (ChatGPT, OpenAI) were not used to generate or interpret scientific content. They were applied solely for minor language editing and R code formatting. All analyses and text were produced and verified by the authors, who retain full responsibility for the scientific content.

## Ethics Statement

The study was conducted in accordance with the Declaration of Helsinki.

## Consent

Given the anonymous study design, the Ethics Committee of Northwestern and Central Switzerland (EKNZ) waived the requirement for written informed consent (EKNZ Req‐2024‐00184).

## Conflicts of Interest

The authors declare no conflicts of interest.

## Supporting information


**Appendix S1:** apa70521‐sup‐0001‐AppendixS1.html.

## Data Availability

The data that support the findings of this study are available from the corresponding author upon reasonable request.
