# Supplementary material for: Emotional and Behavioural Problems in Children With Functional Gastrointestinal Disorders
Source: Acta Paediatr. 2026 Mar 19;115(7):1510–8. doi: 10.1111/apa.70521 (PMC13250960; doi:10.1111/apa.70521)
Supplement: Supplementary file 1 — Appendix S1: apa70521‐sup‐0001‐AppendixS1.html. [file APA-115-1510-s001.html]

Appendix S1. Statistical code and detailed analysis


Code 

- Show All Code
- Hide All Code

# Appendix S1. Statistical code and detailed analysis

#### Sebastian Weber, Anna Geser, Eva Unternährer

#### 2026-01-09

# Clean and prepare workspace

# Settings

Meta-Information: This is the data analysis for CroFam Study 1
focussing on behavioral and emotional problems in children with FGIDs,
comparing results in the functional group versus the somatic and healthy
control groups.

# Install and Load packages

```
#install.packages("tableone", "dplyr", "tidyr", "FSA", "psych", "tidyverse", "lmtest", "pwr","ggplot2", "performance", "car", "effsize", "QuantPsyc", "jtools", "easystats", "janitor", "lm.beta", "stats", "Hmisc", "reshape2", "forcats")
packages <- c("tableone", "dplyr", "tidyr", "FSA", "psych", "tidyverse", "lmtest", "pwr","ggplot2", "performance", "car", "effsize", "QuantPsyc", "jtools", "easystats", "janitor", "lm.beta", "stats", "Hmisc", "reshape2", "forcats")
invisible(lapply(packages, library, character.only = TRUE))
```

# Package citations

Show package citations


tableone (click to expand)

To cite package ‘tableone’ in publications use:

Yoshida K, Bartel A (2022). *tableone: Create ‘Table 1’ to
Describe Baseline Characteristics with or without Propensity Score
Weights*. R package version 0.13.2, https://CRAN.R-project.org/package=tableone.

A BibTeX entry for LaTeX users is

@Manual{, title = {tableone: Create ‘Table
1’ to Describe Baseline Characteristics with or without Propensity Score
Weights}, author = {Kazuki Yoshida and Alexander Bartel}, year = {2022},
note = {R package version 0.13.2}, url = {https://CRAN.R-project.org/package=tableone}, }


dplyr (click to expand)

To cite package ‘dplyr’ in publications use:

Wickham H, François R, Henry L, Müller K, Vaughan D (2023).
*dplyr: A Grammar of Data Manipulation*. R package version 1.1.4,
https://CRAN.R-project.org/package=dplyr.

A BibTeX entry for LaTeX users is

@Manual{, title = {dplyr: A Grammar of
Data Manipulation}, author = {Hadley Wickham and Romain François and
Lionel Henry and Kirill Müller and Davis Vaughan}, year = {2023}, note =
{R package version 1.1.4}, url = {https://CRAN.R-project.org/package=dplyr}, }


tidyr (click to expand)

To cite package ‘tidyr’ in publications use:

Wickham H, Vaughan D, Girlich M (2024). *tidyr: Tidy Messy
Data*. R package version 1.3.1, https://CRAN.R-project.org/package=tidyr.

A BibTeX entry for LaTeX users is

@Manual{, title = {tidyr: Tidy Messy
Data}, author = {Hadley Wickham and Davis Vaughan and Maximilian
Girlich}, year = {2024}, note = {R package version 1.3.1}, url = {https://CRAN.R-project.org/package=tidyr}, }


FSA (click to expand)

To cite package ‘FSA’ in publications use:

Ogle DH, Doll JC, Wheeler AP, Dinno A (2025). *FSA: Simple
Fisheries Stock Assessment Methods*. R package version 0.9.6, https://CRAN.R-project.org/package=FSA.

A BibTeX entry for LaTeX users is

@Manual{, title = {FSA: Simple Fisheries
Stock Assessment Methods}, author = {Derek H. Ogle and Jason C. Doll and
A. Powell Wheeler and Alexis Dinno}, year = {2025}, note = {R package
version 0.9.6}, url = {https://CRAN.R-project.org/package=FSA}, }


psych (click to expand)

To cite package ‘psych’ in publications use:

William Revelle (2025). *psych: Procedures for Psychological,
Psychometric, and Personality Research*. Northwestern University,
Evanston, Illinois. R package version 2.5.3, https://CRAN.R-project.org/package=psych.

A BibTeX entry for LaTeX users is

@Manual{, title = {psych: Procedures for
Psychological, Psychometric, and Personality Research}, author =
{{William Revelle}}, organization = {Northwestern University}, address =
{Evanston, Illinois}, year = {2025}, note = {R package version 2.5.3},
url = {https://CRAN.R-project.org/package=psych}, }


tidyverse (click to expand)

To cite package ‘tidyverse’ in publications use:

Wickham H, Averick M, Bryan J, Chang W, McGowan LD, François R,
Grolemund G, Hayes A, Henry L, Hester J, Kuhn M, Pedersen TL, Miller E,
Bache SM, Müller K, Ooms J, Robinson D, Seidel DP, Spinu V, Takahashi K,
Vaughan D, Wilke C, Woo K, Yutani H (2019). “Welcome to the tidyverse.”
*Journal of Open Source Software*, *4*(43), 1686. doi:10.21105/joss.01686
https://doi.org/10.21105/joss.01686.

A BibTeX entry for LaTeX users is

@Article{, title = {Welcome to the
{tidyverse}}, author = {Hadley Wickham and Mara Averick and Jennifer
Bryan and Winston Chang and Lucy D’Agostino McGowan and Romain François
and Garrett Grolemund and Alex Hayes and Lionel Henry and Jim Hester and
Max Kuhn and Thomas Lin Pedersen and Evan Miller and Stephan Milton
Bache and Kirill Müller and Jeroen Ooms and David Robinson and Dana
Paige Seidel and Vitalie Spinu and Kohske Takahashi and Davis Vaughan
and Claus Wilke and Kara Woo and Hiroaki Yutani}, year = {2019}, journal
= {Journal of Open Source Software}, volume = {4}, number = {43}, pages
= {1686}, doi = {10.21105/joss.01686}, }


lmtest (click to expand)

To cite lmtest in publications use:

Achim Zeileis, Torsten Hothorn (2002). Diagnostic Checking in
Regression Relationships. R News 2(3), 7-10. URL https://CRAN.R-project.org/doc/Rnews/

A BibTeX entry for LaTeX users is

@Article{, title = {Diagnostic Checking in
Regression Relationships}, author = {Achim Zeileis and Torsten Hothorn},
journal = {R News}, year = {2002}, volume = {2}, number = {3}, pages =
{7–10}, url = {https://CRAN.R-project.org/doc/Rnews/}, }


pwr (click to expand)

To cite package ‘pwr’ in publications use:

Champely S (2020). *pwr: Basic Functions for Power Analysis*.
R package version 1.3-0, https://CRAN.R-project.org/package=pwr.

A BibTeX entry for LaTeX users is

@Manual{, title = {pwr: Basic Functions
for Power Analysis}, author = {Stephane Champely}, year = {2020}, note =
{R package version 1.3-0}, url = {https://CRAN.R-project.org/package=pwr}, }


ggplot2 (click to expand)

To cite ggplot2 in publications, please use

H. Wickham. ggplot2: Elegant Graphics for Data Analysis.
Springer-Verlag New York, 2016.

A BibTeX entry for LaTeX users is

@Book{, author = {Hadley Wickham}, title =
{ggplot2: Elegant Graphics for Data Analysis}, publisher =
{Springer-Verlag New York}, year = {2016}, isbn = {978-3-319-24277-4},
url = {https://ggplot2.tidyverse.org}, }


performance (click to expand)

To cite package ‘performance’ in publications use:

Lüdecke et al., (2021). performance: An R Package for Assessment,
Comparison and Testing of Statistical Models. Journal of Open Source
Software, 6(60), 3139. https://doi.org/10.21105/joss.03139

A BibTeX entry for LaTeX users is

@Article{, title = {{performance}: An {R}
Package for Assessment, Comparison and Testing of Statistical Models},
author = {Daniel Lüdecke and Mattan S. Ben-Shachar and Indrajeet Patil
and Philip Waggoner and Dominique Makowski}, year = {2021}, journal =
{Journal of Open Source Software}, volume = {6}, number = {60}, pages =
{3139}, doi = {10.21105/joss.03139}, }


car (click to expand)

To cite the car package in publications use:

Fox J, Weisberg S (2019). *An R Companion to Applied
Regression*, Third edition. Sage, Thousand Oaks CA. https://www.john-fox.ca/Companion/.

A BibTeX entry for LaTeX users is

@Book{, title = {An {R} Companion to
Applied Regression}, edition = {Third}, author = {John Fox and Sanford
Weisberg}, year = {2019}, publisher = {Sage}, address = {Thousand Oaks
{CA}}, url = {https://www.john-fox.ca/Companion/}, }


effsize (click to expand)

To cite package ‘effsize’ in publications use:

Torchiano M (2020). *effsize: Efficient Effect Size
Computation*. doi:10.5281/zenodo.1480624 https://doi.org/10.5281/zenodo.1480624, R package
version 0.8.1, https://CRAN.R-project.org/package=effsize.

A BibTeX entry for LaTeX users is

@Manual{, title = {effsize: Efficient
Effect Size Computation}, author = {Marco Torchiano}, year = {2020},
note = {R package version 0.8.1}, doi = {10.5281/zenodo.1480624}, url =
{https://CRAN.R-project.org/package=effsize}, }


QuantPsyc (click to expand)

To cite package ‘QuantPsyc’ in publications use:

Fletcher TD (2022). *QuantPsyc: Quantitative Psychology
Tools*. R package version 1.6, https://CRAN.R-project.org/package=QuantPsyc.

A BibTeX entry for LaTeX users is

@Manual{, title = {QuantPsyc:
Quantitative Psychology Tools}, author = {Thomas D. Fletcher}, year =
{2022}, note = {R package version 1.6}, url = {https://CRAN.R-project.org/package=QuantPsyc}, }

ATTENTION: This citation information has been auto-generated from the
package DESCRIPTION file and may need manual editing, see
‘help(“citation”)’.


jtools (click to expand)

To cite package ‘jtools’ in publications use:

Long JA (2022). *jtools: Analysis and Presentation of Social
Scientific Data*. R package version 2.2.0, https://cran.r-project.org/package=jtools.

A BibTeX entry for LaTeX users is

@Manual{jtools, title = {jtools: Analysis
and Presentation of Social Scientific Data}, author = {Jacob A. Long},
year = {2022}, note = {R package version 2.2.0}, url = {https://cran.r-project.org/package=jtools}, }


easystats (click to expand)

To cite easystats in publications use:

Lüdecke, Patil, Ben-Shachar, Wiernik, Bacher, Thériault, &
Makowski (2022). easystats: Framework for Easy Statistical Modeling,
Visualization, and Reporting. CRAN. doi:10.32614/CRAN.package.easystats https://doi.org/10.32614/CRAN.package.easystats

A BibTeX entry for LaTeX users is

@Article{, title = {easystats: Framework
for Easy Statistical Modeling, Visualization, and Reporting}, author =
{Daniel Lüdecke and Mattan S. Ben-Shachar and Indrajeet Patil and
Brenton M. Wiernik and Etienne Bacher and Rémi Thériault and Dominique
Makowski}, journal = {CRAN}, doi = {10.32614/CRAN.package.easystats},
year = {2022}, note = {R package}, url = {https://easystats.github.io/easystats/}, }


janitor (click to expand)

To cite package ‘janitor’ in publications use:

Firke S (2024). *janitor: Simple Tools for Examining and Cleaning
Dirty Data*. R package version 2.2.1, https://CRAN.R-project.org/package=janitor.

A BibTeX entry for LaTeX users is

@Manual{, title = {janitor: Simple Tools
for Examining and Cleaning Dirty Data}, author = {Sam Firke}, year =
{2024}, note = {R package version 2.2.1}, url = {https://CRAN.R-project.org/package=janitor}, }


lm.beta (click to expand)

To cite package ‘lm.beta’ in publications use:

Behrendt S (2023). *lm.beta: Add Standardized Regression
Coefficients to Linear-Model-Objects*. R package version 1.7-2, https://CRAN.R-project.org/package=lm.beta.

A BibTeX entry for LaTeX users is

@Manual{, title = {lm.beta: Add
Standardized Regression Coefficients to Linear-Model-Objects}, author =
{Stefan Behrendt}, year = {2023}, note = {R package version 1.7-2}, url
= {https://CRAN.R-project.org/package=lm.beta}, }


stats (click to expand)

The ‘stats’ package is part of R. To cite R in publications use:

R Core Team (2024). *R: A Language and Environment for Statistical
Computing*. R Foundation for Statistical Computing, Vienna, Austria.
https://www.R-project.org/.

A BibTeX entry for LaTeX users is

@Manual{, title = {R: A Language and
Environment for Statistical Computing}, author = {{R Core Team}},
organization = {R Foundation for Statistical Computing}, address =
{Vienna, Austria}, year = {2024}, url = {https://www.R-project.org/}, }

We have invested a lot of time and effort in creating R, please cite it
when using it for data analysis. See also ‘citation(“pkgname”)’ for
citing R packages.


Hmisc (click to expand)

To cite package ‘Hmisc’ in publications use:

Harrell Jr F (2025). *Hmisc: Harrell Miscellaneous*. R package
version 5.2-2, https://CRAN.R-project.org/package=Hmisc.

A BibTeX entry for LaTeX users is

@Manual{, title = {Hmisc: Harrell
Miscellaneous}, author = {Frank E {Harrell Jr}}, year = {2025}, note =
{R package version 5.2-2}, url = {https://CRAN.R-project.org/package=Hmisc}, }


reshape2 (click to expand)

To cite reshape2 in publications use:

Hadley Wickham (2007). Reshaping Data with the reshape Package.
Journal of Statistical Software, 21(12), 1-20. URL http://www.jstatsoft.org/v21/i12/.

A BibTeX entry for LaTeX users is

@Article{, title = {Reshaping Data with
the {reshape} Package}, author = {Hadley Wickham}, journal = {Journal of
Statistical Software}, year = {2007}, volume = {21}, number = {12},
pages = {1–20}, url = {http://www.jstatsoft.org/v21/i12/}, }


forcats (click to expand)

To cite package ‘forcats’ in publications use:

Wickham H (2023). *forcats: Tools for Working with Categorical
Variables (Factors)*. R package version 1.0.0, https://CRAN.R-project.org/package=forcats.

A BibTeX entry for LaTeX users is

@Manual{, title = {forcats: Tools for
Working with Categorical Variables (Factors)}, author = {Hadley
Wickham}, year = {2023}, note = {R package version 1.0.0}, url = {https://CRAN.R-project.org/package=forcats}, }

# Output info

```
cat("The following packages were loaded:\n", paste(packages, collapse = ", "), "\n")
cat("Working directory:", getwd(), "\n")
cat("Loaded dataset:", dataSetInput, "\n")
```

```
## The following packages were loaded:
##  tableone, dplyr, tidyr, FSA, psych, tidyverse, lmtest, pwr, ggplot2, performance, car, effsize, QuantPsyc, jtools, easystats, janitor, lm.beta, stats, Hmisc, reshape2, forcats 
## Working directory: /Users/sebastianweber/switchdrive/Projekt CroFam/5. Data and analysis/5 Analysis 
## Loaded dataset: Input/CroFam_MergingData_250308.RData
```

# Data Import and preparation

```
load(dataSetInput)
dim(mydata) # [1]  239 133
mydata0 <- mydata #create copy

# Exclude healthy controls with SDQ3 >0 (exclusion criteria): 
num_healthy_control_above_0 <- sum(mydata$Group == "Healthy Control" & mydata$SDQ3 > 0)
cat("Excluded healthy controls (SDQ3 > 0):", num_healthy_control_above_0, "\n") # = 21
mydata <- mydata[!(mydata$Group == "Healthy Control" & mydata$SDQ3 > 0), ] # retain 218 participants

# Define factor levels
mydata$Group <- factor(mydata$Group, levels = c("Functional", "Somatic Control", "Healthy Control"))

# Add unique ID
mydata$ID_all <- 1:nrow(mydata)
mydata <- mydata [,c("ID_all", setdiff(names(mydata),"ID_all"))]

# Keep copy with all questionnaires
mydata_all <- mydata 

# Keep only relevant variables: SDQ and PHQ
mydata <- mydata_all %>%
  dplyr::select(-starts_with("SSS"),
                -starts_with("PSS"),
                -starts_with("CDR"),
                -starts_with("BFI"),
                -starts_with("Contributing_factor"),
                -starts_with("Child_Impairment"))

# Data overview 
cat("Group distribution:\n")
print(table(mydata$Group))
cat("\nStructure of dataset:\n")
str(mydata)
```

```
## [1] 239 133
## Excluded healthy controls (SDQ3 > 0): 21 
## Group distribution:
## 
##      Functional Somatic Control Healthy Control 
##              66              62              90 
## 
## Structure of dataset:
## 'data.frame':    218 obs. of  65 variables:
##  $ ID_all                      : int  1 2 3 4 5 6 7 8 9 10 ...
##  $ ID                          : int  1 2 3 4 5 6 7 8 9 10 ...
##  $ Group                       : Factor w/ 3 levels "Functional","Somatic Control",..: 1 1 1 1 1 1 1 1 1 1 ...
##  $ DateCompleted               : Date, format: "2024-02-06" "2024-02-09" ...
##  $ Parent1                     : Factor w/ 2 levels "Mother","Father": 1 1 1 1 2 1 2 1 1 1 ...
##  $ ChildAge                    : num  12 9 8 6 3 5 9 7 9 10 ...
##  $ ChildGender                 : Factor w/ 3 levels "Male","Female",..: 2 1 2 2 2 1 2 2 2 1 ...
##  $ ParentsSeparated            : logi  FALSE FALSE TRUE TRUE FALSE FALSE ...
##  $ Parent1_Age                 : num  47 36 NA NA NA NA NA NA NA 43 ...
##  $ Parent1_Work_Fulltime       : logi  TRUE TRUE FALSE FALSE TRUE FALSE ...
##  $ Parent1_Work_Parttime       : logi  FALSE FALSE TRUE TRUE FALSE TRUE ...
##  $ Parent1_Work_Percent        : num  100 100 90 60 100 40 0 35 100 60 ...
##  $ Parent1_Work_Household      : logi  FALSE FALSE FALSE FALSE FALSE FALSE ...
##  $ Parent1_Work_Student        : logi  FALSE FALSE FALSE FALSE FALSE FALSE ...
##  $ Parent1_Work_Unemployed     : logi  FALSE FALSE FALSE FALSE FALSE FALSE ...
##  $ Parent1_Work_SocialInsurance: logi  FALSE FALSE FALSE FALSE FALSE FALSE ...
##  $ Parent1_Education           : Factor w/ 7 levels "No completed schooling",..: 7 7 7 3 4 3 2 4 5 3 ...
##  $ Parent2_Work_Fulltime       : logi  TRUE FALSE TRUE TRUE FALSE TRUE ...
##  $ Parent2_Work_Parttime       : logi  FALSE TRUE FALSE FALSE TRUE FALSE ...
##  $ Parent2_Work_Percent        : num  100 80 100 100 60 100 0 100 100 100 ...
##  $ Parent2_Work_Household      : logi  FALSE FALSE FALSE FALSE FALSE FALSE ...
##  $ Parent2_Work_Student        : logi  FALSE FALSE FALSE FALSE FALSE FALSE ...
##  $ Parent2_Work_Unemployed     : logi  FALSE FALSE FALSE FALSE FALSE FALSE ...
##  $ Parent2_Work_SocialInsurance: logi  FALSE FALSE FALSE FALSE FALSE FALSE ...
##  $ Parent2_Work_Unknown        : logi  FALSE FALSE FALSE FALSE FALSE FALSE ...
##  $ Parent2_Education           : Factor w/ 8 levels "No completed schooling",..: 7 7 7 2 7 3 8 7 7 5 ...
##  $ Comments                    : chr  "" "" "" "" ...
##  $ SDQ1                        : num  2 2 2 2 2 2 2 2 2 2 ...
##  $ SDQ2                        : num  0 1 1 2 0 1 0 1 0 0 ...
##  $ SDQ3                        : num  0 0 2 1 0 2 0 2 2 1 ...
##  $ SDQ4                        : num  2 2 2 2 1 0 1 2 2 2 ...
##  $ SDQ5                        : num  0 1 1 0 0 2 0 1 0 1 ...
##  $ SDQ6                        : num  0 0 1 0 1 0 1 2 0 0 ...
##  $ SDQ7                        : num  0 0 1 1 0 1 1 0 1 0 ...
##  $ SDQ8                        : num  0 0 2 1 0 0 1 0 1 0 ...
##  $ SDQ9                        : num  2 2 2 0 2 0 0 2 2 2 ...
##  $ SDQ10                       : num  0 1 0 1 0 2 0 1 0 0 ...
##  $ SDQ11                       : num  0 0 0 2 1 1 1 0 0 1 ...
##  $ SDQ12                       : num  0 0 1 0 0 1 1 1 0 0 ...
##  $ SDQ13                       : num  0 0 0 1 0 1 1 1 0 0 ...
##  $ SDQ14                       : num  0 0 1 0 0 1 0 0 0 1 ...
##  $ SDQ15                       : num  0 1 2 1 0 1 0 1 0 0 ...
##  $ SDQ16                       : num  0 1 2 0 2 0 0 2 1 1 ...
##  $ SDQ17                       : num  2 2 2 2 2 1 1 2 2 2 ...
##  $ SDQ18                       : num  0 0 0 0 0 1 0 1 0 0 ...
##  $ SDQ19                       : num  0 0 1 0 0 0 0 1 0 1 ...
##  $ SDQ20                       : num  2 2 1 2 1 0 0 2 2 2 ...
##  $ SDQ21                       : num  0 0 1 1 1 1 0 0 0 0 ...
##  $ SDQ22                       : num  0 0 0 0 0 1 0 1 0 0 ...
##  $ SDQ23                       : num  0 0 0 1 1 0 1 0 0 1 ...
##  $ SDQ24                       : num  0 0 1 0 0 0 0 1 0 1 ...
##  $ SDQ25                       : num  0 1 2 1 1 2 1 1 1 1 ...
##  $ SDQ_Emotional               : num  0 1 7 3 2 3 2 6 4 3 ...
##  $ SDQ_Conduct                 : num  0 1 3 1 0 6 2 4 1 1 ...
##  $ SDQ_Hyperact                : num  0 4 6 6 2 7 1 4 1 1 ...
##  $ SDQ_PeerProb                : num  0 0 3 3 3 2 3 3 0 4 ...
##  $ SDQ_Prosocial               : num  10 10 9 8 8 3 4 10 10 10 ...
##  $ SDQ_Total                   : num  0 6 19 13 7 18 8 17 6 9 ...
##  $ PHQ1                        : num  0 0 0 0 2 0 2 1 0 1 ...
##  $ PHQ2                        : num  0 0 0 0 0 1 1 0 0 1 ...
##  $ PHQ3                        : num  0 0 0 0 1 3 1 1 1 1 ...
##  $ PHQ4                        : num  0 0 0 0 1 2 0 1 2 0 ...
##  $ PHQ_Depression              : num  0 0 0 0 2 1 3 1 0 2 ...
##  $ PHQ_Anxiety                 : num  0 0 0 0 2 5 1 2 3 1 ...
##  $ PHQ_Total                   : num  0 0 0 0 4 6 4 3 3 3 ...
```

The raw data contains N=218 cases and 65 variables. The variable
names are: ID\_all, ID, Group, DateCompleted, Parent1, ChildAge,
ChildGender, ParentsSeparated, Parent1\_Age, Parent1\_Work\_Fulltime,
Parent1\_Work\_Parttime, Parent1\_Work\_Percent, Parent1\_Work\_Household,
Parent1\_Work\_Student, Parent1\_Work\_Unemployed,
Parent1\_Work\_SocialInsurance, Parent1\_Education, Parent2\_Work\_Fulltime,
Parent2\_Work\_Parttime, Parent2\_Work\_Percent, Parent2\_Work\_Household,
Parent2\_Work\_Student, Parent2\_Work\_Unemployed,
Parent2\_Work\_SocialInsurance, Parent2\_Work\_Unknown, Parent2\_Education,
Comments, SDQ1, SDQ2, SDQ3, SDQ4, SDQ5, SDQ6, SDQ7, SDQ8, SDQ9, SDQ10,
SDQ11, SDQ12, SDQ13, SDQ14, SDQ15, SDQ16, SDQ17, SDQ18, SDQ19, SDQ20,
SDQ21, SDQ22, SDQ23, SDQ24, SDQ25, SDQ\_Emotional, SDQ\_Conduct,
SDQ\_Hyperact, SDQ\_PeerProb, SDQ\_Prosocial, SDQ\_Total, PHQ1, PHQ2, PHQ3,
PHQ4, PHQ\_Depression, PHQ\_Anxiety, PHQ\_Total

# BASELINE CHARACTERISTICS ANALYSIS —

## Create combined variables for Baseline & Checking missing values

```
# --- Gender Child ---
  # Confirm absence of non-binary entries for readability.
  sum_nonbinary <- sum(mydata$ChildGender == "non-binär/Gender-Divers", na.rm = TRUE)
  cat("Non-binary entries:", sum_nonbinary, "\n")  # 0 expected
  mydata$ChildGender <-factor(mydata$ChildGender, levels = c("Male", "Female")) 

# --- Parent 1: Employment & Education ---
  # New Variable: Grouping of no paid work Parent1
  mydata$Parent1_Work_currently_no_paid_work <- with(mydata, 
    (Parent1_Work_Household | Parent1_Work_Student | Parent1_Work_Unemployed | Parent1_Work_SocialInsurance) & 
    !(Parent1_Work_Parttime | Parent1_Work_Fulltime))
  # Summarize Variable for Parent1_Work status
  mydata$Parent1_Work <- ifelse(mydata$Parent1_Work_Parttime, "Parttime", ifelse(mydata$Parent1_Work_Fulltime, "Fulltime", ifelse(mydata$Parent1_Work_currently_no_paid_work, "No_paid_work", NA)))
  mydata$Parent1_Work <- factor(mydata$Parent1_Work, levels = c("Fulltime", "Parttime", "No_paid_work"), ordered = TRUE)
  
  # New Variable: Grouping Educational Level Parent1 
  mydata$Parent1_Education_level <- factor(mydata$Parent1_Education, levels = c("University", "University of applied sciences", "Higher vocational training ", "High school diploma", "Apprenticeship" , "Compulsory education", "No completed schooling"), labels = c("high", "high", "intermediate", "intermediate", "intermediate", "low", "low"), ordered = TRUE) 

# --- Parent 2: Employment & Education ---
  # New Variable: Grouping of no paid work Parent2
  mydata$Parent2_Work_currently_no_paid_work <- with(mydata, 
    (Parent2_Work_Household | Parent2_Work_Student | Parent2_Work_Unemployed | Parent2_Work_SocialInsurance) & 
    !(Parent2_Work_Parttime | Parent2_Work_Fulltime))
  # New variable to summarize work Parent 2
  mydata$Parent2_Work <- ifelse(mydata$Parent2_Work_Parttime, "Parttime", ifelse(mydata$Parent2_Work_Fulltime, "Fulltime", ifelse(mydata$Parent2_Work_Unknown, "Unknown", ifelse(mydata$Parent2_Work_currently_no_paid_work, "No_paid_work", NA)))) 
  mydata$Parent2_Work <- factor(mydata$Parent2_Work, levels = c("Fulltime", "Parttime", "No_paid_work", "Unknown"), ordered = TRUE)
  
  # New Variable: Grouping of Educational Level Parent2
  mydata$Parent2_Education_level <- factor(mydata$Parent2_Education, levels = c("University", "University of applied sciences", "Higher vocational training ", "High school diploma", "Apprenticeship" , "Compulsory education", "No completed schooling", "Unknown"), labels = c("high", "high", "intermediate", "intermediate", "intermediate", "low", "low", "unknown"), ordered = TRUE) 
  
# --- Missing values summary for baseline table footnotes ---
  vars_to_check <- c("Parent1", "ChildAge", "ChildGender", "ParentsSeparated",
"Parent1_Age", "Parent1_Education_level", "Parent1_Work",
"Parent2_Education_level", "Parent2_Work", "SDQ_Total", "PHQ_Total")
  na_counts <- sapply(vars_to_check, function(var) sum(is.na(mydata[[var]])))
  cat("\nMissing values per variable:\n")
  print(na_counts)

# --- PHQ missing per group ---
  missing_values <- mydata %>%
  dplyr::group_by(Group) %>%
  dplyr::summarise(missing_PHQ = sum(is.na(PHQ_Total)), .groups = "drop")
  cat("\nMissing PHQ values per group:\n")
  print(missing_values)
```

```
## Non-binary entries: 0 
## 
## Missing values per variable:
##                 Parent1                ChildAge             ChildGender 
##                       2                       0                       0 
##        ParentsSeparated             Parent1_Age Parent1_Education_level 
##                       1                      15                       1 
##            Parent1_Work Parent2_Education_level            Parent2_Work 
##                       0                       2                       0 
##               SDQ_Total               PHQ_Total 
##                       0                       1 
## 
## Missing PHQ values per group:
## # A tibble: 3 × 2
##   Group           missing_PHQ
##   <fct>                 <int>
## 1 Functional                1
## 2 Somatic Control           0
## 3 Healthy Control           0
```

## Baseline: completed questionnaires and sex of index parent

```
# Summary table: n per group, number and % mothers
  bl_Basic <- mydata %>%
  dplyr::group_by(Group) %>%
  dplyr::summarise(
  n = n(),
  Mother = sum(Parent1 == "Mother", na.rm = TRUE),
  PercentMother = round(Mother / n * 100, 2))
# Print table neatly formatted for HTML output
  knitr::kable(bl_Basic, caption = "Baseline: Parent respondent (Mother/Father) by group")

# Chi-square test: Parent1 (Mother/Father) by Group ---
  chisq_Parent1 <- chisq.test(table(mydata$Parent1, mydata$Group))
  cat("\nChi-square test results:\n")
  print(chisq_Parent1)

# Overall frequencies of Parent1 (not stratified) ---
  cat("\nOverall distribution of Parent1:\n")
  print(table(mydata$Parent1))
  cat("\nPercent distribution of Parent1:\n")
  print(round(prop.table(table(mydata$Parent1)) * 100, 1))
```

Baseline: Parent respondent (Mother/Father) by group

| Group | n | Mother | PercentMother |
| --- | --- | --- | --- |
| Functional | 66 | 50 | 75.76 |
| Somatic Control | 62 | 42 | 67.74 |
| Healthy Control | 90 | 69 | 76.67 |

```
## 
## Chi-square test results:
## 
##  Pearson's Chi-squared test
## 
## data:  table(mydata$Parent1, mydata$Group)
## X-squared = 1.5099, df = 2, p-value = 0.47
## 
## 
## Overall distribution of Parent1:
## 
## Mother Father 
##    161     55 
## 
## Percent distribution of Parent1:
## 
## Mother Father 
##   74.5   25.5
```

## Baseline: Child characteristics and parental separation

```
# Table ChildAge, Gender, and Parental Separation by Group
bl_child <- CreateTableOne(
  vars   = c("ChildAge", "ChildGender", "ParentsSeparated"),
  data   = mydata,
  strata = "Group")
tbl_child <- print(bl_child, showAllLevels = TRUE, quote = FALSE, noSpaces = TRUE, printToggle = FALSE)
knitr::kable(as.data.frame(tbl_child),caption = "Baseline: Child characteristics and parental separation by group")
invisible(NULL)

# Overall descriptive statistics (not stratified)
  cat("\nDescriptive statistics for full sample:\n")
  psych::describe(mydata$ChildAge)
  cat("\nGender distribution (n, %):\n")
  gender_tab <- table(mydata$ChildGender)
  print(gender_tab)
  print(round(prop.table(gender_tab) * 100, 1))
  cat("\nParental separation status (n, %):\n")
  sep_tab <- table(mydata$ParentsSeparated)
  print(sep_tab)
  print(round(prop.table(sep_tab) * 100, 1))

# Child age: continuous variable ---
  cat("\nNormality test (Shapiro-Wilk):\n")
  print(shapiro.test(mydata$ChildAge)) # not normal
  cat("\nKruskal-Wallis test for ChildAge by Group:\n")
  kruskal_ChildAge <- kruskal.test(ChildAge ~ Group, data = mydata)
  print(kruskal_ChildAge)
  cat("\nPost-hoc Dunn test for ChildAge (Bonferroni-adjusted):\n")
  dunn_ChildAge <- dunnTest(ChildAge ~ Group, data = mydata, method = "bonferroni")
  print(dunn_ChildAge)
  
# Child gender: categorical variable ---
  cat("\nChi-square test for ChildGender by Group:\n")
  ChildGender_table <- table(mydata$ChildGender, mydata$Group)
  chi_ChildGender <- chisq.test(ChildGender_table)
  print(chi_ChildGender)

# Parents separation status: Categorical -> Chi-Quadrat-Test
  mydata$ParentsSeparated <- factor(mydata$ParentsSeparated, levels = c(FALSE, TRUE), labels = c("No", "Yes"))
  table_parents_separated <- table(mydata$Group, mydata$ParentsSeparated)
  table_parents_separated_percent <- prop.table(table_parents_separated, margin = 1) * 100
  table_parents_separated_combined <- cbind(table_parents_separated, table_parents_separated_percent)
  round(table_parents_separated_percent, 1)
  cat("\nParental separation by group (n and % within group):\n")
  print(table_parents_separated_combined)
  cat("\nChi-square test for ParentsSeparated by Group:\n")
  print(chisq.test(table(mydata$ParentsSeparated, mydata$Group)))
  cat("\nPairwise comparison (Bonferroni-adjusted):\n")
  pair_sep <- pairwise.prop.test(table(mydata$Group, mydata$ParentsSeparated), p.adjust.method = "bonferroni")
  print(pair_sep)
```

Baseline: Child characteristics and parental separation by
group


|  | level | Functional | Somatic Control | Healthy Control | p | test |
| --- | --- | --- | --- | --- | --- | --- |
| n |  | 66 | 62 | 90 |  |  |
| ChildAge..mean..SD.. |  | 6.56 (2.80) | 8.15 (3.03) | 7.27 (2.78) | 0.008 |  |
| ChildGender…. | Male | 34 (51.5) | 30 (48.4) | 49 (54.4) | 0.762 |  |
| X | Female | 32 (48.5) | 32 (51.6) | 41 (45.6) |  |  |
| ParentsSeparated…. | FALSE | 42 (64.6) | 50 (80.6) | 77 (85.6) | 0.007 |  |
| X.1 | TRUE | 23 (35.4) | 12 (19.4) | 13 (14.4) |  |  |

```
## 
## Descriptive statistics for full sample:
```

```
## 
## Gender distribution (n, %):
## 
##   Male Female 
##    113    105 
## 
##   Male Female 
##   51.8   48.2 
## 
## Parental separation status (n, %):
## 
## FALSE  TRUE 
##   169    48 
## 
## FALSE  TRUE 
##  77.9  22.1 
## 
## Normality test (Shapiro-Wilk):
## 
##  Shapiro-Wilk normality test
## 
## data:  mydata$ChildAge
## W = 0.94852, p-value = 5.175e-07
## 
## 
## Kruskal-Wallis test for ChildAge by Group:
## 
##  Kruskal-Wallis rank sum test
## 
## data:  ChildAge by Group
## Kruskal-Wallis chi-squared = 9.7967, df = 2, p-value = 0.007459
## 
## 
## Post-hoc Dunn test for ChildAge (Bonferroni-adjusted):
##                          Comparison         Z     P.unadj       P.adj
## 1      Functional - Healthy Control -1.524238 0.127449223 0.382347668
## 2      Functional - Somatic Control -3.126310 0.001770151 0.005310453
## 3 Healthy Control - Somatic Control -1.853512 0.063809001 0.191427003
## 
## Chi-square test for ChildGender by Group:
## 
##  Pearson's Chi-squared test
## 
## data:  ChildGender_table
## X-squared = 0.54339, df = 2, p-value = 0.7621
## 
##                  
##                     No  Yes
##   Functional      64.6 35.4
##   Somatic Control 80.6 19.4
##   Healthy Control 85.6 14.4
## 
## Parental separation by group (n and % within group):
##                 No Yes       No      Yes
## Functional      42  23 64.61538 35.38462
## Somatic Control 50  12 80.64516 19.35484
## Healthy Control 77  13 85.55556 14.44444
## 
## Chi-square test for ParentsSeparated by Group:
## 
##  Pearson's Chi-squared test
## 
## data:  table(mydata$ParentsSeparated, mydata$Group)
## X-squared = 9.9919, df = 2, p-value = 0.006765
## 
## 
## Pairwise comparison (Bonferroni-adjusted):
## 
##  Pairwise comparisons using Pairwise comparison of proportions 
## 
## data:  table(mydata$Group, mydata$ParentsSeparated) 
## 
##                 Functional Somatic Control
## Somatic Control 0.205      -              
## Healthy Control 0.013      1.000          
## 
## P value adjustment method: bonferroni
```

## Baseline: Indexparent work, education and age

```
# Table for Educational level and Working status
  Parent1_Work_columns <- grep("^Parent1_Work", names(mydata), value = TRUE)
  variables_to_include <- c("Parent1_Education", "Parent1_Education_level", Parent1_Work_columns)
  bl_parent1 <- CreateTableOne(
  vars = variables_to_include,
  data = mydata,strata = "Group")
  tbl_parent1 <- print(bl_parent1,, showAllLevels = TRUE, quote = FALSE, noSpaces = TRUE, printToggle = FALSE)
knitr::kable(as.data.frame(tbl_parent1), caption = "Baseline: Index parent education and work status by group")
invisible(NULL) 

# --- Parent1 Work: ordinal variable ---
  cat("\nKruskal-Wallis test for Parent1_Work by Group:\n")
  print(kruskal.test(mydata$Parent1_Work ~ mydata$Group))
  cat("\nKruskal-Wallis test for Parent1_Work_Percent by Group (if available):\n")
  if ("Parent1_Work_Percent" %in% names(mydata)) {print(kruskal.test(mydata$Parent1_Work_Percent ~ mydata$Group))}

# --- Parent1 Education Level: ordinal variable ---
  cat("\nKruskal-Wallis test for Parent1_Education_level by Group:\n")
  print(kruskal.test(mydata$Parent1_Education_level ~ mydata$Group))
  mydata$Parent1_Education_level_num <- as.numeric(mydata$Parent1_Education_level)
  cat("\nPost-hoc Dunn test for Parent1_Education_level (Bonferroni-adjusted):\n")
  dunn_Parent1_Education_level <- dunnTest(Parent1_Education_level_num ~ Group,data = mydata, method = "bonferroni")
  print(dunn_Parent1_Education_level)

# --- Parent1 Age summary ---
  Parent1_age <- mydata %>% dplyr::group_by(Group) %>% dplyr::summarise(
median_Parent1_Age = median(Parent1_Age, na.rm = TRUE),
iqr_Parent1_Age = IQR(Parent1_Age, na.rm = TRUE),
mean_Parent1_Age = mean(Parent1_Age, na.rm = TRUE),
sd_Parent1_Age = sd(Parent1_Age, na.rm = TRUE))
  knitr::kable(Parent1_age, caption = "Parent1 Age by group (mean, SD, median, IQR)")
  
# --- Parent1 Age: continuous variable ---
  cat("\nNormality test (Shapiro-Wilk) for Parent1_Age:\n")
  print(shapiro.test(mydata$Parent1_Age))
  cat("\nKruskal-Wallis test for Parent1_Age by Group:\n")
  kruskal_Parent1_Age <- kruskal.test(Parent1_Age ~ Group, data = mydata)
  print(kruskal_Parent1_Age)
  cat("\nANOVA (robustness check):\n")
  anova_Parent1_Age <- aov(Parent1_Age ~ Group, data = mydata)
  print(summary(anova_Parent1_Age))
```

Baseline: Index parent education and work status by
group


|  | level | Functional | Somatic Control | Healthy Control | p | test |
| --- | --- | --- | --- | --- | --- | --- |
| n |  | 66 | 62 | 90 |  |  |
| Parent1\_Education…. | No completed schooling | 0 (0.0) | 1 (1.6) | 0 (0.0) | 0.004 |  |
| X | Compulsory education | 5 (7.7) | 0 (0.0) | 3 (3.3) |  |  |
| X.1 | Apprenticeship | 15 (23.1) | 12 (19.4) | 8 (8.9) |  |  |
| X.2 | High school diploma | 2 (3.1) | 1 (1.6) | 1 (1.1) |  |  |
| X.3 | Higher vocational training | 8 (12.3) | 11 (17.7) | 7 (7.8) |  |  |
| X.4 | University of applied sciences | 8 (12.3) | 14 (22.6) | 10 (11.1) |  |  |
| X.5 | University | 27 (41.5) | 23 (37.1) | 61 (67.8) |  |  |
| Parent1\_Education\_level…. | high | 35 (53.8) | 37 (59.7) | 71 (78.9) | 0.005 |  |
| X.6 | intermediate | 25 (38.5) | 24 (38.7) | 16 (17.8) |  |  |
| X.7 | low | 5 (7.7) | 1 (1.6) | 3 (3.3) |  |  |
| Parent1\_Work\_Fulltime…. | FALSE | 47 (71.2) | 47 (75.8) | 65 (72.2) | 0.826 |  |
| X.8 | TRUE | 19 (28.8) | 15 (24.2) | 25 (27.8) |  |  |
| Parent1\_Work\_Parttime…. | FALSE | 30 (45.5) | 20 (32.3) | 36 (40.0) | 0.309 |  |
| X.9 | TRUE | 36 (54.5) | 42 (67.7) | 54 (60.0) |  |  |
| Parent1\_Work\_Percent..mean..SD.. |  | 61.97 (35.10) | 67.74 (28.87) | 62.78 (32.57) | 0.546 |  |
| Parent1\_Work\_Household…. | FALSE | 59 (89.4) | 58 (93.5) | 77 (85.6) | 0.300 |  |
| X.10 | TRUE | 7 (10.6) | 4 (6.5) | 13 (14.4) |  |  |
| Parent1\_Work\_Student…. | FALSE | 66 (100.0) | 62 (100.0) | 88 (97.8) | 0.238 |  |
| X.11 | TRUE | 0 (0.0) | 0 (0.0) | 2 (2.2) |  |  |
| Parent1\_Work\_Unemployed…. | FALSE | 63 (95.5) | 61 (98.4) | 87 (96.7) | 0.640 |  |
| X.12 | TRUE | 3 (4.5) | 1 (1.6) | 3 (3.3) |  |  |
| Parent1\_Work\_SocialInsurance…. | FALSE | 64 (97.0) | 62 (100.0) | 90 (100.0) | 0.098 |  |
| X.13 | TRUE | 2 (3.0) | 0 (0.0) | 0 (0.0) |  |  |
| Parent1\_Work\_currently\_no\_paid\_work…. | FALSE | 55 (83.3) | 57 (91.9) | 79 (87.8) | 0.336 |  |
| X.14 | TRUE | 11 (16.7) | 5 (8.1) | 11 (12.2) |  |  |
| Parent1\_Work…. | Fulltime | 19 (28.8) | 15 (24.2) | 25 (27.8) | 0.538 |  |
| X.15 | Parttime | 36 (54.5) | 42 (67.7) | 54 (60.0) |  |  |
| X.16 | No\_paid\_work | 11 (16.7) | 5 (8.1) | 11 (12.2) |  |  |

```
## 
## Kruskal-Wallis test for Parent1_Work by Group:
## 
##  Kruskal-Wallis rank sum test
## 
## data:  mydata$Parent1_Work by mydata$Group
## Kruskal-Wallis chi-squared = 0.097348, df = 2, p-value = 0.9525
## 
## 
## Kruskal-Wallis test for Parent1_Work_Percent by Group (if available):
## 
##  Kruskal-Wallis rank sum test
## 
## data:  mydata$Parent1_Work_Percent by mydata$Group
## Kruskal-Wallis chi-squared = 0.80226, df = 2, p-value = 0.6696
## 
## 
## Kruskal-Wallis test for Parent1_Education_level by Group:
## 
##  Kruskal-Wallis rank sum test
## 
## data:  mydata$Parent1_Education_level by mydata$Group
## Kruskal-Wallis chi-squared = 11.643, df = 2, p-value = 0.002964
## 
## 
## Post-hoc Dunn test for Parent1_Education_level (Bonferroni-adjusted):
##                          Comparison          Z     P.unadj       P.adj
## 1      Functional - Healthy Control  3.2651931 0.001093895 0.003281685
## 2      Functional - Somatic Control  0.9000006 0.368119943 1.000000000
## 3 Healthy Control - Somatic Control -2.2522416 0.024307008 0.072921025
```

Parent1 Age by group (mean, SD, median, IQR)


| Group | median\_Parent1\_Age | iqr\_Parent1\_Age | mean\_Parent1\_Age | sd\_Parent1\_Age |
| --- | --- | --- | --- | --- |
| Functional | 40 | 8 | 40.10169 | 6.359069 |
| Somatic Control | 41 | 7 | 41.16364 | 5.849228 |
| Healthy Control | 41 | 7 | 41.68539 | 5.656042 |

```
## 
## Normality test (Shapiro-Wilk) for Parent1_Age:
## 
##  Shapiro-Wilk normality test
## 
## data:  mydata$Parent1_Age
## W = 0.98526, p-value = 0.03251
## 
## 
## Kruskal-Wallis test for Parent1_Age by Group:
## 
##  Kruskal-Wallis rank sum test
## 
## data:  Parent1_Age by Group
## Kruskal-Wallis chi-squared = 3.3404, df = 2, p-value = 0.1882
## 
## 
## ANOVA (robustness check):
##              Df Sum Sq Mean Sq F value Pr(>F)
## Group         2     89   44.73   1.277  0.281
## Residuals   200   7008   35.04               
## 15 observations deleted due to missingness
```

## Baseline: Second parent (Parent2) education and work

```
# Table for Educational level and Working status
  Parent2_Work_columns <- grep("^Parent2_Work", names(mydata), value = TRUE)
  variables_to_include <- c("Parent2_Education", "Parent2_Education_level", Parent2_Work_columns)
  bl_parent2 <- CreateTableOne(
  vars = variables_to_include,
  data = mydata,
  strata = "Group")
  tbl_parent2 <- print(bl_parent2,, showAllLevels = TRUE, quote = FALSE, noSpaces = TRUE, printToggle = FALSE)
knitr::kable(as.data.frame(tbl_parent2), caption = "Baseline: Second parent education and work status by group")
invisible(NULL) 

# Parent2_Work: ordinal variable
  cat("\nKruskal-Wallis test for Parent2_Work by Group:\n")
  print(kruskal.test(mydata$Parent2_Work ~ mydata$Group))
  cat("\nKruskal-Wallis test for Parent2_Work_Percent by Group (if available):\n")
  if ("Parent2_Work_Percent" %in% names(mydata)) {print(kruskal.test(mydata$Parent2_Work_Percent ~ mydata$Group))}

# Parent1_Education_level: ordinal variable
  cat("\nKruskal-Wallis test for Parent2_Education_level by Group:\n")
  print(kruskal.test(mydata$Parent2_Education_level ~ mydata$Group))
  mydata$Parent2_Education_level_num <- as.numeric(mydata$Parent2_Education_level)
  cat("\nPost-hoc Dunn test for Parent2_Education_level (Bonferroni-adjusted):\n")
  dunn_Parent2_Education_level <- dunnTest(Parent2_Education_level_num ~ Group, data = mydata, method = "bonferroni")
  print(dunn_Parent2_Education_level)
```

Baseline: Second parent education and work status by
group


|  | level | Functional | Somatic Control | Healthy Control | p | test |
| --- | --- | --- | --- | --- | --- | --- |
| n |  | 66 | 62 | 90 |  |  |
| Parent2\_Education…. | No completed schooling | 0 (0.0) | 0 (0.0) | 0 (0.0) | NaN |  |
| X | Compulsory education | 4 (6.2) | 4 (6.5) | 3 (3.3) |  |  |
| X.1 | Apprenticeship | 10 (15.6) | 10 (16.1) | 12 (13.3) |  |  |
| X.2 | High school diploma | 1 (1.6) | 2 (3.2) | 3 (3.3) |  |  |
| X.3 | Higher vocational training | 6 (9.4) | 3 (4.8) | 4 (4.4) |  |  |
| X.4 | University of applied sciences | 10 (15.6) | 13 (21.0) | 11 (12.2) |  |  |
| X.5 | University | 30 (46.9) | 30 (48.4) | 56 (62.2) |  |  |
| X.6 | Unknown | 3 (4.7) | 0 (0.0) | 1 (1.1) |  |  |
| Parent2\_Education\_level…. | high | 40 (62.5) | 43 (69.4) | 67 (74.4) | 0.381 |  |
| X.7 | intermediate | 17 (26.6) | 15 (24.2) | 19 (21.1) |  |  |
| X.8 | low | 4 (6.2) | 4 (6.5) | 3 (3.3) |  |  |
| X.9 | unknown | 3 (4.7) | 0 (0.0) | 1 (1.1) |  |  |
| Parent2\_Work\_Fulltime…. | FALSE | 31 (47.0) | 27 (43.5) | 43 (47.8) | 0.870 |  |
| X.10 | TRUE | 35 (53.0) | 35 (56.5) | 47 (52.2) |  |  |
| Parent2\_Work\_Parttime…. | FALSE | 45 (68.2) | 42 (67.7) | 53 (58.9) | 0.387 |  |
| X.11 | TRUE | 21 (31.8) | 20 (32.3) | 37 (41.1) |  |  |
| Parent2\_Work\_Percent..mean..SD.. |  | 77.94 (34.13) | 79.67 (31.46) | 83.69 (24.50) | 0.471 |  |
| Parent2\_Work\_Household…. | FALSE | 64 (97.0) | 59 (95.2) | 84 (93.3) | 0.589 |  |
| X.12 | TRUE | 2 (3.0) | 3 (4.8) | 6 (6.7) |  |  |
| Parent2\_Work\_Student…. | FALSE | 66 (100.0) | 61 (98.4) | 89 (98.9) | 0.613 |  |
| X.13 | TRUE | 0 (0.0) | 1 (1.6) | 1 (1.1) |  |  |
| Parent2\_Work\_Unemployed…. | FALSE | 60 (90.9) | 62 (100.0) | 89 (98.9) | 0.005 |  |
| X.14 | TRUE | 6 (9.1) | 0 (0.0) | 1 (1.1) |  |  |
| Parent2\_Work\_SocialInsurance…. | FALSE | 64 (97.0) | 60 (96.8) | 89 (98.9) | 0.618 |  |
| X.15 | TRUE | 2 (3.0) | 2 (3.2) | 1 (1.1) |  |  |
| Parent2\_Work\_Unknown…. | FALSE | 63 (95.5) | 61 (98.4) | 88 (97.8) | 0.552 |  |
| X.16 | TRUE | 3 (4.5) | 1 (1.6) | 2 (2.2) |  |  |
| Parent2\_Work\_currently\_no\_paid\_work…. | FALSE | 58 (87.9) | 56 (90.3) | 86 (95.6) | 0.203 |  |
| X.17 | TRUE | 8 (12.1) | 6 (9.7) | 4 (4.4) |  |  |
| Parent2\_Work…. | Fulltime | 34 (51.5) | 35 (56.5) | 47 (52.2) | 0.484 |  |
| X.18 | Parttime | 21 (31.8) | 20 (32.3) | 37 (41.1) |  |  |
| X.19 | No\_paid\_work | 8 (12.1) | 6 (9.7) | 4 (4.4) |  |  |
| X.20 | Unknown | 3 (4.5) | 1 (1.6) | 2 (2.2) |  |  |

```
## 
## Kruskal-Wallis test for Parent2_Work by Group:
## 
##  Kruskal-Wallis rank sum test
## 
## data:  mydata$Parent2_Work by mydata$Group
## Kruskal-Wallis chi-squared = 0.66437, df = 2, p-value = 0.7174
## 
## 
## Kruskal-Wallis test for Parent2_Work_Percent by Group (if available):
## 
##  Kruskal-Wallis rank sum test
## 
## data:  mydata$Parent2_Work_Percent by mydata$Group
## Kruskal-Wallis chi-squared = 0.057618, df = 2, p-value = 0.9716
## 
## 
## Kruskal-Wallis test for Parent2_Education_level by Group:
## 
##  Kruskal-Wallis rank sum test
## 
## data:  mydata$Parent2_Education_level by mydata$Group
## Kruskal-Wallis chi-squared = 2.9993, df = 2, p-value = 0.2232
## 
## 
## Post-hoc Dunn test for Parent2_Education_level (Bonferroni-adjusted):
##                          Comparison          Z    P.unadj     P.adj
## 1      Functional - Healthy Control  1.7314514 0.08337128 0.2501139
## 2      Functional - Somatic Control  0.9599499 0.33708041 1.0000000
## 3 Healthy Control - Somatic Control -0.6789198 0.49718866 1.0000000
```

# Spearman correlation matrix

```
# Calculates pairwise correlations between ordinal or metric variables. Note: Not appropriate for nominal/categorical data.
  x <- data.matrix(mydata[,c("ChildAge", "Parent1_Age", "Parent1_Education_level", "Parent2_Education_level", "Parent1_Work", "Parent2_Work", "PHQ_Depression", "PHQ_Anxiety", "PHQ_Total", "SDQ_Emotional", "SDQ_Conduct", "SDQ_Hyperact", "SDQ_PeerProb", "SDQ_Prosocial", "SDQ_Total")])
  tp <- "spearman"
  R <- rcorr(x, type = tp)$r #r = korrelationcoefficient (from -1 to 1), 0.8-1/-.08--1 very strong, 0.6-0.8 strong, 0.4-0.6 moderate, 0.2-0.4 weak, 0-0.2 very weak/no correlation
  p <- rcorr(x, type = tp)$P #P is p-value
  n <- rcorr(x, type = tp)$n #n count of observations per each correlation
  mystars <- ifelse(p < .001, "***", ifelse(p < .01, "** ", ifelse(p < .05, "* ", " "))) #defines p values 
  R <- format(round(cbind(rep(-1.11, ncol(x)), R), 2))[,-1] 
  Rnew <- matrix(paste(R, mystars, sep=""), ncol=ncol(x)) 
  diag(Rnew) <- paste(diag(R), " ", sep="") 
  rownames(Rnew) <- colnames(x) 
  colnames(Rnew) <- paste(colnames(x), "", sep="") 
  Rnew <- as.matrix(Rnew)
  Rnew[upper.tri(Rnew, diag = TRUE)] <- ""
  Rnew <- as.data.frame(Rnew) 
  Rnew <- cbind(Rnew[1:length(Rnew)-1])
  cat("\nSpearman correlation matrix (lower triangle, with significance stars):\n")
  print(knitr::kable(Rnew, caption = "Spearman correlation coefficients (Spearman’s ρ)"))
  
# Visualization
  cor_spearman_matrix <- rcorr(x, type = "spearman")$r 
  cor_matrix_melt <- melt(cor_spearman_matrix)
  ggplot(cor_matrix_melt, aes(Var1, Var2, fill = value)) + geom_tile() + scale_fill_gradient2(low = "red", high = "green", mid = "white", midpoint = 0) + theme_minimal() + labs(title = "Spearman Correlation Heatmap", x = "Variables", y = "Variables") +
  theme(axis.text.x = element_text(angle = 45, hjust = 1)) +
  theme(axis.text.y = element_text(angle = 0, hjust = 1))
```

```
## 
## Spearman correlation matrix (lower triangle, with significance stars):
## 
## 
## Table: Spearman correlation coefficients (Spearman’s ρ)
## 
## |                        |ChildAge |Parent1_Age |Parent1_Education_level |Parent2_Education_level |Parent1_Work |Parent2_Work |PHQ_Depression |PHQ_Anxiety |PHQ_Total |SDQ_Emotional |SDQ_Conduct |SDQ_Hyperact |SDQ_PeerProb |SDQ_Prosocial |
## |:-----------------------|:--------|:-----------|:-----------------------|:-----------------------|:------------|:------------|:--------------|:-----------|:---------|:-------------|:-----------|:------------|:------------|:-------------|
## |ChildAge                |         |            |                        |                        |             |             |               |            |          |              |            |             |             |              |
## |Parent1_Age             |0.53***  |            |                        |                        |             |             |               |            |          |              |            |             |             |              |
## |Parent1_Education_level |0.01     |0.01        |                        |                        |             |             |               |            |          |              |            |             |             |              |
## |Parent2_Education_level |0.02     |-0.01       |0.52***                 |                        |             |             |               |            |          |              |            |             |             |              |
## |Parent1_Work            |-0.06    |-0.08       |0.13                    |0.04                    |             |             |               |            |          |              |            |             |             |              |
## |Parent2_Work            |-0.06    |-0.07       |0.08                    |0.14*                   |-0.19**      |             |               |            |          |              |            |             |             |              |
## |PHQ_Depression          |-0.16*   |-0.04       |0.16*                   |0.20**                  |0.14*        |-0.04        |               |            |          |              |            |             |             |              |
## |PHQ_Anxiety             |-0.18**  |-0.08       |0.16*                   |0.10                    |0.12         |-0.03        |0.57***        |            |          |              |            |             |             |              |
## |PHQ_Total               |-0.18**  |-0.08       |0.19**                  |0.17*                   |0.14*        |-0.03        |0.83***        |0.91***     |          |              |            |             |             |              |
## |SDQ_Emotional           |-0.17*   |-0.11       |0.18**                  |0.18**                  |0.05         |0.05         |0.46***        |0.44***     |0.51***   |              |            |             |             |              |
## |SDQ_Conduct             |-0.18**  |-0.03       |0.12                    |0.13                    |0.00         |0.10         |0.38***        |0.25***     |0.33***   |0.44***       |            |             |             |              |
## |SDQ_Hyperact            |-0.21**  |-0.09       |0.11                    |0.07                    |0.04         |0.03         |0.26***        |0.23***     |0.26***   |0.32***       |0.59***     |             |             |              |
## |SDQ_PeerProb            |-0.15*   |-0.05       |0.14*                   |0.14*                   |0.03         |0.07         |0.35***        |0.30***     |0.36***   |0.43***       |0.38***     |0.34***      |             |              |
## |SDQ_Prosocial           |0.26***  |0.07        |-0.11                   |-0.07                   |-0.09        |-0.11        |-0.32***       |-0.25***    |-0.31***  |-0.44***      |-0.59***    |-0.47***     |-0.46***     |              |
## |SDQ_Total               |-0.25*** |-0.11       |0.17*                   |0.16*                   |0.06         |0.10         |0.48***        |0.41***     |0.49***   |0.72***       |0.79***     |0.78***      |0.64***      |-0.64***      |
```

# Pearson Point-Biserial Correlation for SDQ Total

```
# Tests correlations between dichotomous (0/1) and continuous variables (e.g., SDQ_Total)
  # Ensure binary coding (0/1)
mydata$ChildGender <- ifelse(mydata$ChildGender == "Male", 0, 1) #Levels: Male Female
mydata$Parent1 <- ifelse(mydata$Parent1 == "Mother", 0, 1) #Levels: Mother Father
mydata$ParentsSeparated <- ifelse(mydata$ParentsSeparated == "No", 0, 1) #Levels: no, yes

cor_PB_Parent1 <- cor(mydata$SDQ_Total, mydata$Parent1, method = "pearson", use = "complete.obs") #1NA
cor_PB_ChildGender <- cor(mydata$SDQ_Total, mydata$ChildGender, method = "pearson", use = "complete.obs") #0 NA
cor_PB_ParentsSeparated <- cor(mydata$SDQ_Total, mydata$ParentsSeparated, method = "pearson", use = "complete.obs") #1NA

n_complete_Parent1 <- sum(complete.cases(mydata$SDQ_Total, mydata$Parent1))
n_complete_ChildGender <- sum(complete.cases(mydata$SDQ_Total, mydata$ChildGender))
n_complete_ParentsSeparated <- sum(complete.cases(mydata$SDQ_Total, mydata$ParentsSeparated))
cat("Complete observations Parent1: ", n_complete_Parent1, "\n")
cat("Complete observations ChildGender: ", n_complete_ChildGender, "\n")
cat("Complete observations ParentsSeparated: ", n_complete_ParentsSeparated, "\n")

cat("Point-Biserial Correlation Parent1 and SDQ Total: ", cor_PB_Parent1, "\n") 
cat("Point-Biserial Correlatio ChildGender and SDQ Total: ", cor_PB_ChildGender, "\n")  
cat("Point-Biserial Correlatio ParentsSeparated and SDQ Total: ", cor_PB_ParentsSeparated, "\n") 

# Variables to investigate
target_var <- "SDQ_Total"
dichotomous_var <- c("Parent1", "ChildGender", "ParentsSeparated")
correlations <- list()
for (target in target_var) {
  for (dichotomous in dichotomous_var) {
    cor_test_result <- cor.test(mydata[[target]], mydata[[dichotomous]], method = "pearson", use = "complete.obs")
    cor_result <- cor_test_result$estimate
    p_value <- cor_test_result$p.value
    if (p_value < 0.001) {
      significance <- "***"
      } else if (p_value < 0.01) {
      significance <- "**"
    } else if (p_value < 0.05) {
      significance <- "*"
    } else {
      significance <- "not significant"}
    correlations[[paste(target, "and", dichotomous)]] <- list(correlation = cor_result, p_value = p_value, significance = significance)
    n_complete <- sum(complete.cases(mydata[[target]], mydata[[dichotomous]]))
    cat("Number of complete observations for", target, "and", dichotomous, ":", n_complete, "\n")
    cat("Point-Biserial Correlation between", target, "and", dichotomous, ":", cor_result, significance, "\n")}}
cat("\nAll Point-Biserial Correlations and p-values with Significance:\n")
for (key in names(correlations)) {
  cat(key, ": Correlation = ", correlations[[key]]$correlation, ", p-value = ", correlations[[key]]$p_value, 
      ", Significance = ", correlations[[key]]$significance, "\n")}

# Visualization
cor_data <- data.frame(
  Variable = c("Parent1", "ChildGender", "ParentsSeparated"),
  Correlation = c(cor_PB_Parent1, cor_PB_ChildGender, cor_PB_ParentsSeparated))
cor_matrix_melted <- reshape2::melt(cor_data, id.vars = "Variable")
cor_matrix_melted$Variable <- factor(cor_matrix_melted$Variable, 
                                      levels = c("Parent1", "ChildGender", "ParentsSeparated"),
                                      labels = c("Parent1 (0=Mother, 1=Father)", 
                                                 "ChildGender (0=Male, 1=Female)", 
                                                 "ParentsSeparated (0= Not Seperated, 1= Separated)"))
ggplot(cor_matrix_melted, aes(x = Variable, y = value, fill = value)) +
  geom_col() +
  scale_fill_gradient2(low = "red", high = "green", mid = "white", midpoint = 0, limits = c(-0.4, 0.4)) +
  theme_minimal() +
  labs(title = "Point-Biserial Correlations SDQ Total and dichotomous Variabels", 
       x = "Dichotome Variablen", 
       y = "Korrelation",
       fill = "Korrelation") +
  theme(axis.text.x = element_text(angle = 45, hjust = 1))
```

```
## Complete observations Parent1:  216 
## Complete observations ChildGender:  218 
## Complete observations ParentsSeparated:  217 
## Point-Biserial Correlation Parent1 and SDQ Total:  -0.08588653 
## Point-Biserial Correlatio ChildGender and SDQ Total:  -0.1477905 
## Point-Biserial Correlatio ParentsSeparated and SDQ Total:  0.2675716 
## Number of complete observations for SDQ_Total and Parent1 : 216 
## Point-Biserial Correlation between SDQ_Total and Parent1 : -0.08588653 not significant 
## Number of complete observations for SDQ_Total and ChildGender : 218 
## Point-Biserial Correlation between SDQ_Total and ChildGender : -0.1477905 * 
## Number of complete observations for SDQ_Total and ParentsSeparated : 217 
## Point-Biserial Correlation between SDQ_Total and ParentsSeparated : 0.2675716 *** 
## 
## All Point-Biserial Correlations and p-values with Significance:
## SDQ_Total and Parent1 : Correlation =  -0.08588653 , p-value =  0.2086562 , Significance =  not significant 
## SDQ_Total and ChildGender : Correlation =  -0.1477905 , p-value =  0.02914283 , Significance =  * 
## SDQ_Total and ParentsSeparated : Correlation =  0.2675716 , p-value =  6.556269e-05 , Significance =  ***
```

# Prepare imputed data

```
# CAVE: Do not impute dependent variable (SDQ_Total)
# Overview of missing data
  na_counts <- sapply(vars_to_check, function(var) sum(is.na(mydata[[var]])))
  cat("\nMissing values per variable:\n")
  print(na_counts)

# Select variables for imputation (exclude depentend var missings) 
  mydata_SDQ_impute <- mydata[!is.na(mydata$SDQ_Total), c("Group", "Parent1", "ChildAge", "ChildGender", "ParentsSeparated", "Parent1_Age", "Parent1_Education_level", "Parent1_Work", "Parent2_Education_level", "Parent2_Work", "PHQ_Total","PHQ_Depression", "PHQ_Anxiety", "SDQ_Total", "SDQ_Emotional", "SDQ_Conduct", "SDQ_Hyperact", "SDQ_PeerProb", "SDQ_Prosocial")] #no SDQ Missings
  cat("\nStructure of dataset used for imputation:\n")
  str(mydata_SDQ_impute) #all numeric / factor / ordinal factor

  # Perform multiple imputation using MICE 
  library(mice)
  imp_SDQ <- mice(mydata_SDQ_impute, m = 5, seed = 500)
  cat("\nImputation summary:\n")
  print(summary(imp_SDQ))
  mydata_SDQ_impute <- complete(imp_SDQ, 1) 
  cat("\nRemaining missing values after imputation: ",
    sum(is.na(mydata_SDQ_impute)), "\n")
  # Final imputed dataset: mydata_SDQ_impute
```

```
## 
## Missing values per variable:
##                 Parent1                ChildAge             ChildGender 
##                       2                       0                       0 
##        ParentsSeparated             Parent1_Age Parent1_Education_level 
##                       1                      15                       1 
##            Parent1_Work Parent2_Education_level            Parent2_Work 
##                       0                       2                       0 
##               SDQ_Total               PHQ_Total 
##                       0                       1 
## 
## Structure of dataset used for imputation:
## 'data.frame':    218 obs. of  19 variables:
##  $ Group                  : Factor w/ 3 levels "Functional","Somatic Control",..: 1 1 1 1 1 1 1 1 1 1 ...
##  $ Parent1                : num  0 0 0 0 1 0 1 0 0 0 ...
##  $ ChildAge               : num  12 9 8 6 3 5 9 7 9 10 ...
##  $ ChildGender            : num  1 0 1 1 1 0 1 1 1 0 ...
##  $ ParentsSeparated       : num  0 0 1 1 0 0 1 0 0 0 ...
##  $ Parent1_Age            : num  47 36 NA NA NA NA NA NA NA 43 ...
##  $ Parent1_Education_level: Ord.factor w/ 3 levels "high"<"intermediate"<..: 1 1 1 2 2 2 3 2 2 2 ...
##  $ Parent1_Work           : Ord.factor w/ 3 levels "Fulltime"<"Parttime"<..: 1 1 2 2 1 2 3 2 1 2 ...
##  $ Parent2_Education_level: Ord.factor w/ 4 levels "high"<"intermediate"<..: 1 1 1 3 1 2 4 1 1 2 ...
##  $ Parent2_Work           : Ord.factor w/ 4 levels "Fulltime"<"Parttime"<..: 1 2 1 1 2 1 3 1 1 1 ...
##  $ PHQ_Total              : num  0 0 0 0 4 6 4 3 3 3 ...
##  $ PHQ_Depression         : num  0 0 0 0 2 1 3 1 0 2 ...
##  $ PHQ_Anxiety            : num  0 0 0 0 2 5 1 2 3 1 ...
##  $ SDQ_Total              : num  0 6 19 13 7 18 8 17 6 9 ...
##  $ SDQ_Emotional          : num  0 1 7 3 2 3 2 6 4 3 ...
##  $ SDQ_Conduct            : num  0 1 3 1 0 6 2 4 1 1 ...
##  $ SDQ_Hyperact           : num  0 4 6 6 2 7 1 4 1 1 ...
##  $ SDQ_PeerProb           : num  0 0 3 3 3 2 3 3 0 4 ...
##  $ SDQ_Prosocial          : num  10 10 9 8 8 3 4 10 10 10 ...
## 
##  iter imp variable
##   1   1  Parent1  ParentsSeparated  Parent1_Age  Parent1_Education_level  Parent2_Education_level  PHQ_Total  PHQ_Depression  PHQ_Anxiety
##   1   2  Parent1  ParentsSeparated  Parent1_Age  Parent1_Education_level  Parent2_Education_level  PHQ_Total  PHQ_Depression  PHQ_Anxiety
##   1   3  Parent1  ParentsSeparated  Parent1_Age  Parent1_Education_level  Parent2_Education_level  PHQ_Total  PHQ_Depression  PHQ_Anxiety
##   1   4  Parent1  ParentsSeparated  Parent1_Age  Parent1_Education_level  Parent2_Education_level  PHQ_Total  PHQ_Depression  PHQ_Anxiety
##   1   5  Parent1  ParentsSeparated  Parent1_Age  Parent1_Education_level  Parent2_Education_level  PHQ_Total  PHQ_Depression  PHQ_Anxiety
##   2   1  Parent1  ParentsSeparated  Parent1_Age  Parent1_Education_level  Parent2_Education_level  PHQ_Total  PHQ_Depression  PHQ_Anxiety
##   2   2  Parent1  ParentsSeparated  Parent1_Age  Parent1_Education_level  Parent2_Education_level  PHQ_Total  PHQ_Depression  PHQ_Anxiety
##   2   3  Parent1  ParentsSeparated  Parent1_Age  Parent1_Education_level  Parent2_Education_level  PHQ_Total  PHQ_Depression  PHQ_Anxiety
##   2   4  Parent1  ParentsSeparated  Parent1_Age  Parent1_Education_level  Parent2_Education_level  PHQ_Total  PHQ_Depression  PHQ_Anxiety
##   2   5  Parent1  ParentsSeparated  Parent1_Age  Parent1_Education_level  Parent2_Education_level  PHQ_Total  PHQ_Depression  PHQ_Anxiety
##   3   1  Parent1  ParentsSeparated  Parent1_Age  Parent1_Education_level  Parent2_Education_level  PHQ_Total  PHQ_Depression  PHQ_Anxiety
##   3   2  Parent1  ParentsSeparated  Parent1_Age  Parent1_Education_level  Parent2_Education_level  PHQ_Total  PHQ_Depression  PHQ_Anxiety
##   3   3  Parent1  ParentsSeparated  Parent1_Age  Parent1_Education_level  Parent2_Education_level  PHQ_Total  PHQ_Depression  PHQ_Anxiety
##   3   4  Parent1  ParentsSeparated  Parent1_Age  Parent1_Education_level  Parent2_Education_level  PHQ_Total  PHQ_Depression  PHQ_Anxiety
##   3   5  Parent1  ParentsSeparated  Parent1_Age  Parent1_Education_level  Parent2_Education_level  PHQ_Total  PHQ_Depression  PHQ_Anxiety
##   4   1  Parent1  ParentsSeparated  Parent1_Age  Parent1_Education_level  Parent2_Education_level  PHQ_Total  PHQ_Depression  PHQ_Anxiety
##   4   2  Parent1  ParentsSeparated  Parent1_Age  Parent1_Education_level  Parent2_Education_level  PHQ_Total  PHQ_Depression  PHQ_Anxiety
##   4   3  Parent1  ParentsSeparated  Parent1_Age  Parent1_Education_level  Parent2_Education_level  PHQ_Total  PHQ_Depression  PHQ_Anxiety
##   4   4  Parent1  ParentsSeparated  Parent1_Age  Parent1_Education_level  Parent2_Education_level  PHQ_Total  PHQ_Depression  PHQ_Anxiety
##   4   5  Parent1  ParentsSeparated  Parent1_Age  Parent1_Education_level  Parent2_Education_level  PHQ_Total  PHQ_Depression  PHQ_Anxiety
##   5   1  Parent1  ParentsSeparated  Parent1_Age  Parent1_Education_level  Parent2_Education_level  PHQ_Total  PHQ_Depression  PHQ_Anxiety
##   5   2  Parent1  ParentsSeparated  Parent1_Age  Parent1_Education_level  Parent2_Education_level  PHQ_Total  PHQ_Depression  PHQ_Anxiety
##   5   3  Parent1  ParentsSeparated  Parent1_Age  Parent1_Education_level  Parent2_Education_level  PHQ_Total  PHQ_Depression  PHQ_Anxiety
##   5   4  Parent1  ParentsSeparated  Parent1_Age  Parent1_Education_level  Parent2_Education_level  PHQ_Total  PHQ_Depression  PHQ_Anxiety
##   5   5  Parent1  ParentsSeparated  Parent1_Age  Parent1_Education_level  Parent2_Education_level  PHQ_Total  PHQ_Depression  PHQ_Anxiety
## 
## Imputation summary:
## Class: mids
## Number of multiple imputations:  5 
## Imputation methods:
##                   Group                 Parent1                ChildAge 
##                      ""                   "pmm"                      "" 
##             ChildGender        ParentsSeparated             Parent1_Age 
##                      ""                   "pmm"                   "pmm" 
## Parent1_Education_level            Parent1_Work Parent2_Education_level 
##                  "polr"                      ""                  "polr" 
##            Parent2_Work               PHQ_Total          PHQ_Depression 
##                      ""                   "pmm"                   "pmm" 
##             PHQ_Anxiety               SDQ_Total           SDQ_Emotional 
##                   "pmm"                      ""                      "" 
##             SDQ_Conduct            SDQ_Hyperact            SDQ_PeerProb 
##                      ""                      ""                      "" 
##           SDQ_Prosocial 
##                      "" 
## PredictorMatrix:
##                  Group Parent1 ChildAge ChildGender ParentsSeparated
## Group                0       1        1           1                1
## Parent1              1       0        1           1                1
## ChildAge             1       1        0           1                1
## ChildGender          1       1        1           0                1
## ParentsSeparated     1       1        1           1                0
## Parent1_Age          1       1        1           1                1
##                  Parent1_Age Parent1_Education_level Parent1_Work
## Group                      1                       1            1
## Parent1                    1                       1            1
## ChildAge                   1                       1            1
## ChildGender                1                       1            1
## ParentsSeparated           1                       1            1
## Parent1_Age                0                       1            1
##                  Parent2_Education_level Parent2_Work PHQ_Total PHQ_Depression
## Group                                  1            1         1              1
## Parent1                                1            1         1              1
## ChildAge                               1            1         1              1
## ChildGender                            1            1         1              1
## ParentsSeparated                       1            1         1              1
## Parent1_Age                            1            1         1              1
##                  PHQ_Anxiety SDQ_Total SDQ_Emotional SDQ_Conduct SDQ_Hyperact
## Group                      1         1             1           1            1
## Parent1                    1         1             1           1            1
## ChildAge                   1         1             1           1            1
## ChildGender                1         1             1           1            1
## ParentsSeparated           1         1             1           1            1
## Parent1_Age                1         1             1           1            1
##                  SDQ_PeerProb SDQ_Prosocial
## Group                       1             1
## Parent1                     1             1
## ChildAge                    1             1
## ChildGender                 1             1
## ParentsSeparated            1             1
## Parent1_Age                 1             1
## Number of logged events:  200 
##   it im                     dep meth                  out
## 1  1  1                 Parent1  pmm            SDQ_Total
## 2  1  1        ParentsSeparated  pmm            SDQ_Total
## 3  1  1             Parent1_Age  pmm            SDQ_Total
## 4  1  1 Parent1_Education_level polr            SDQ_Total
## 5  1  1 Parent2_Education_level polr PHQ_Total, SDQ_Total
## 6  1  1               PHQ_Total  pmm            SDQ_Total
## Class: mids
## Number of multiple imputations:  5 
## Imputation methods:
##                   Group                 Parent1                ChildAge 
##                      ""                   "pmm"                      "" 
##             ChildGender        ParentsSeparated             Parent1_Age 
##                      ""                   "pmm"                   "pmm" 
## Parent1_Education_level            Parent1_Work Parent2_Education_level 
##                  "polr"                      ""                  "polr" 
##            Parent2_Work               PHQ_Total          PHQ_Depression 
##                      ""                   "pmm"                   "pmm" 
##             PHQ_Anxiety               SDQ_Total           SDQ_Emotional 
##                   "pmm"                      ""                      "" 
##             SDQ_Conduct            SDQ_Hyperact            SDQ_PeerProb 
##                      ""                      ""                      "" 
##           SDQ_Prosocial 
##                      "" 
## PredictorMatrix:
##                  Group Parent1 ChildAge ChildGender ParentsSeparated
## Group                0       1        1           1                1
## Parent1              1       0        1           1                1
## ChildAge             1       1        0           1                1
## ChildGender          1       1        1           0                1
## ParentsSeparated     1       1        1           1                0
## Parent1_Age          1       1        1           1                1
##                  Parent1_Age Parent1_Education_level Parent1_Work
## Group                      1                       1            1
## Parent1                    1                       1            1
## ChildAge                   1                       1            1
## ChildGender                1                       1            1
## ParentsSeparated           1                       1            1
## Parent1_Age                0                       1            1
##                  Parent2_Education_level Parent2_Work PHQ_Total PHQ_Depression
## Group                                  1            1         1              1
## Parent1                                1            1         1              1
## ChildAge                               1            1         1              1
## ChildGender                            1            1         1              1
## ParentsSeparated                       1            1         1              1
## Parent1_Age                            1            1         1              1
##                  PHQ_Anxiety SDQ_Total SDQ_Emotional SDQ_Conduct SDQ_Hyperact
## Group                      1         1             1           1            1
## Parent1                    1         1             1           1            1
## ChildAge                   1         1             1           1            1
## ChildGender                1         1             1           1            1
## ParentsSeparated           1         1             1           1            1
## Parent1_Age                1         1             1           1            1
##                  SDQ_PeerProb SDQ_Prosocial
## Group                       1             1
## Parent1                     1             1
## ChildAge                    1             1
## ChildGender                 1             1
## ParentsSeparated            1             1
## Parent1_Age                 1             1
## Number of logged events:  200 
##   it im                     dep meth                  out
## 1  1  1                 Parent1  pmm            SDQ_Total
## 2  1  1        ParentsSeparated  pmm            SDQ_Total
## 3  1  1             Parent1_Age  pmm            SDQ_Total
## 4  1  1 Parent1_Education_level polr            SDQ_Total
## 5  1  1 Parent2_Education_level polr PHQ_Total, SDQ_Total
## 6  1  1               PHQ_Total  pmm            SDQ_Total
## 
## Remaining missing values after imputation:  0
```

# SDQ —

## Operationalization of Research Question

```
### Research Question:
    # Does the prevalence of behavioral and emotional problems, as measured by SDQ scores, differ between children with FGIDs and those without FGIDs?

### Hypothesis:
    # H1a: Children with FGIDs have significantly higher SDQ total scores, suggesting a higher prevalence of behavioral and emotional problems than controls. 
    # H2a: Children with FGIDs will exhibit significantly higher SDQ emotional problems subscores.(reflecting internalizing symptoms such as anxiety/depression)
    # H2b: Children with FGIDs will show significantly higher SDQ conduct  subscores. (reflecting more behavioral problems)
    # H2c: Children with FGIDs will have higher SDQ hyperactivity/inattention subscores. (reflecting more attention and regulation difficulties)

### Operationalization:
    # DV: SDQ_Total 
    # UV: Group
    # CV: Covariate = "ChildGender", "ChildAge", "ParentsSeparated", "PHQ_Total", "Parent1_Education_level"
      # Not used as CV: "Parent1_Age", "Parent1"
```

## Preliminary Data Analysis

```
### Descriptive statistics for each SDQ subscale by Group
SDQ_descriptive <- mydata  %>%
  group_by(Group) %>%
  summarise(
    across(
      starts_with("SDQ_"), list(Mean = ~round(mean(. , na.rm = TRUE), 2),
                               SD = ~round(sd(. , na.rm = TRUE), 2),
                               Skewness = ~round(skew(. , na.rm = TRUE), 2),
                               Kurtosis = ~round(kurtosi(. , na.rm = TRUE), 2))))  %>% 
   pivot_longer(
    cols = -Group,  
    names_to = "Statistic",
    values_to = "Value") %>%
  pivot_wider(
    names_from = Group,    
    values_from = Value)
knitr::kable(SDQ_descriptive, caption = "Descriptive statistics for SDQ subscales by group (Mean, SD, Skewness, Kurtosis).")

# Summary table: Mean (SD)
SDQ_descriptive_table <- mydata %>%
  group_by(Group) %>%
  summarise(
    SDQ_Emotional = paste0(round(mean(SDQ_Emotional, na.rm = TRUE), 2), " (", round(sd(SDQ_Emotional, na.rm = TRUE), 2), ")"),
    SDQ_Conduct = paste0(round(mean(SDQ_Conduct, na.rm = TRUE), 2), " (", round(sd(SDQ_Conduct, na.rm = TRUE), 2), ")"),
    SDQ_Hyperact = paste0(round(mean(SDQ_Hyperact, na.rm = TRUE), 2), " (", round(sd(SDQ_Hyperact, na.rm = TRUE), 2), ")"),
    SDQ_PeerProb = paste0(round(mean(SDQ_PeerProb, na.rm = TRUE), 2), " (", round(sd(SDQ_PeerProb, na.rm = TRUE), 2), ")"),
    SDQ_Prosocial = paste0(round(mean(SDQ_Prosocial, na.rm = TRUE), 2), " (", round(sd(SDQ_Prosocial, na.rm = TRUE), 2), ")"),
    SDQ_Total = paste0(round(mean(SDQ_Total, na.rm = TRUE), 2), " (", round(sd(SDQ_Total, na.rm = TRUE), 2), ")")
  ) %>%
  pivot_longer(
    cols = -Group, 
    names_to = "SDQ_Score",
    values_to = "Mean_SD") %>%
  pivot_wider(
    names_from = Group,
    values_from = Mean_SD)
knitr::kable(SDQ_descriptive_table, caption = "Mean (SD) of SDQ total and subscale scores by group.")
```

Descriptive statistics for SDQ subscales by group (Mean, SD,
Skewness, Kurtosis).

| Statistic | Functional | Somatic Control | Healthy Control |
| --- | --- | --- | --- |
| SDQ\_Emotional\_Mean | 3.92 | 1.27 | 1.14 |
| SDQ\_Emotional\_SD | 2.42 | 1.78 | 1.28 |
| SDQ\_Emotional\_Skewness | 0.47 | 1.59 | 1.17 |
| SDQ\_Emotional\_Kurtosis | -0.32 | 1.64 | 1.27 |
| SDQ\_Conduct\_Mean | 3.08 | 1.00 | 1.58 |
| SDQ\_Conduct\_SD | 2.78 | 1.67 | 1.56 |
| SDQ\_Conduct\_Skewness | 0.74 | 1.73 | 0.94 |
| SDQ\_Conduct\_Kurtosis | -0.65 | 1.81 | -0.01 |
| SDQ\_Hyperact\_Mean | 4.48 | 2.31 | 2.76 |
| SDQ\_Hyperact\_SD | 2.89 | 1.81 | 2.43 |
| SDQ\_Hyperact\_Skewness | 0.16 | 0.45 | 0.87 |
| SDQ\_Hyperact\_Kurtosis | -1.06 | -0.79 | 0.31 |
| SDQ\_PeerProb\_Mean | 2.09 | 0.44 | 1.04 |
| SDQ\_PeerProb\_SD | 2.12 | 0.88 | 1.36 |
| SDQ\_PeerProb\_Skewness | 1.20 | 2.04 | 1.52 |
| SDQ\_PeerProb\_Kurtosis | 1.14 | 3.69 | 2.67 |
| SDQ\_Prosocial\_Mean | 7.07 | 9.52 | 8.12 |
| SDQ\_Prosocial\_SD | 2.40 | 1.13 | 1.65 |
| SDQ\_Prosocial\_Skewness | -0.59 | -2.64 | -1.33 |
| SDQ\_Prosocial\_Kurtosis | -0.36 | 6.66 | 2.68 |
| SDQ\_Total\_Mean | 13.58 | 5.02 | 6.52 |
| SDQ\_Total\_SD | 6.55 | 4.74 | 4.73 |
| SDQ\_Total\_Skewness | 0.28 | 1.28 | 0.75 |
| SDQ\_Total\_Kurtosis | -0.88 | 1.33 | 0.21 |

Mean (SD) of SDQ total and subscale scores by group.

| SDQ\_Score | Functional | Somatic Control | Healthy Control |
| --- | --- | --- | --- |
| SDQ\_Emotional | 3.92 (2.42) | 1.27 (1.78) | 1.14 (1.28) |
| SDQ\_Conduct | 3.08 (2.78) | 1 (1.67) | 1.58 (1.56) |
| SDQ\_Hyperact | 4.48 (2.89) | 2.31 (1.81) | 2.76 (2.43) |
| SDQ\_PeerProb | 2.09 (2.12) | 0.44 (0.88) | 1.04 (1.36) |
| SDQ\_Prosocial | 7.07 (2.4) | 9.52 (1.13) | 8.12 (1.65) |
| SDQ\_Total | 13.58 (6.55) | 5.02 (4.74) | 6.52 (4.73) |

## SDQ Clinical cut-offs

```
## SDQ cut-offs (age-adjusted: 2–3y vs. 4–17y), descriptive by group
# 4–17y parent: Normal 0–13, Borderline 14–16, Abnormal 17–40
# 2–4y parent (3-band derived from official 4-band): Normal 0–12, Borderline 13–15, Abnormal 16–40

SDQ_cutoffs <- mydata %>%
  mutate(
    age_band = case_when(
      !is.na(ChildAge) & ChildAge < 4 ~ "2-4_parent",
      !is.na(ChildAge) & ChildAge >= 4 ~ "4-17_parent",
      TRUE ~ NA_character_),
    SDQ_Total_cat = case_when(
      # 2–4y (3-band)
      age_band == "2-4_parent" & SDQ_Total <= 12 ~ "Normal",
      age_band == "2-4_parent" & SDQ_Total <= 15 ~ "Borderline",
      age_band == "2-4_parent" & SDQ_Total >= 16 ~ "Abnormal",
      # 4–17y
      age_band == "4-17_parent" & SDQ_Total <= 13 ~ "Normal",
      age_band == "4-17_parent" & SDQ_Total <= 16 ~ "Borderline",
      age_band == "4-17_parent" & SDQ_Total >= 17 ~ "Abnormal",
      TRUE ~ NA_character_),
    SDQ_Total_cat = factor(SDQ_Total_cat, levels = c("Normal", "Borderline", "Abnormal")),
    SDQ_Total_elevated = if_else(SDQ_Total_cat %in% c("Borderline", "Abnormal"), 1L, 0L)
  ) %>%
  group_by(Group) %>%
  summarise(
    N = sum(!is.na(SDQ_Total_cat)),
    Normal_n = sum(SDQ_Total_cat == "Normal", na.rm = TRUE),
    Borderline_n = sum(SDQ_Total_cat == "Borderline", na.rm = TRUE),
    Abnormal_n = sum(SDQ_Total_cat == "Abnormal", na.rm = TRUE),
    Normal_pct = round(100 * Normal_n / N, 1),
    Borderline_pct = round(100 * Borderline_n / N, 1),
    Abnormal_pct = round(100 * Abnormal_n / N, 1),
    Elevated_n = sum(SDQ_Total_elevated == 1L, na.rm = TRUE),
    Elevated_pct = round(100 * Elevated_n / N, 1))
knitr::kable(
  SDQ_cutoffs,
  caption = "Age-adjusted SDQ Total Difficulties categories (Normal/Borderline/Abnormal) and elevated proportion (Borderline+Abnormal) by group")

SDQ_elevated_sentence_ageadj <- SDQ_cutoffs %>%
  transmute(Group, txt = paste0(Elevated_pct, "% (", Elevated_n, "/", N, ")")) %>%
  tidyr::pivot_wider(names_from = Group, values_from = txt)
knitr::kable(
  SDQ_elevated_sentence_ageadj,
  caption = "Summary: Elevated SDQ Total (Borderline+Abnormal), age-adjusted cut-offs.")

# figure
SDQ_cutoffs_plotdf <- mydata %>%
  mutate(
    age_band = case_when(
      !is.na(ChildAge) & ChildAge < 4 ~ "2-4_parent",
      !is.na(ChildAge) & ChildAge >= 4 ~ "4-17_parent",
      TRUE ~ NA_character_),
    SDQ_Total_cat = case_when(
      age_band == "2-4_parent" & SDQ_Total <= 12 ~ "Normal",
      age_band == "2-4_parent" & SDQ_Total <= 15 ~ "Borderline",
      age_band == "2-4_parent" & SDQ_Total >= 16 ~ "Abnormal",
      age_band == "4-17_parent" & SDQ_Total <= 13 ~ "Normal",
      age_band == "4-17_parent" & SDQ_Total <= 16 ~ "Borderline",
      age_band == "4-17_parent" & SDQ_Total >= 17 ~ "Abnormal",
      TRUE ~ NA_character_
    ),
    SDQ_Total_cat = factor(SDQ_Total_cat, levels = c("Normal", "Borderline", "Abnormal"))
  ) %>%
  filter(!is.na(Group), !is.na(SDQ_Total_cat)) %>%
  count(Group, SDQ_Total_cat, name = "n") %>%
  group_by(Group) %>%
  mutate(
    N = sum(n),
    pct = 100 * n / N
  ) %>%
  ungroup()

p_SDQ_cutoffs_S1 <- ggplot(SDQ_cutoffs_plotdf, aes(x = Group, y = pct, fill = SDQ_Total_cat)) +
  geom_col(width = 0.7) +
  scale_y_continuous(limits = c(0, 100), expand = c(0, 0),
                     labels = function(x) paste0(round(x), "%")) +
  labs(
    x = NULL,
    y = "Proportion of children",
    fill = "SDQ total difficulties",
    caption = "Age-adjusted cut-offs: 2–4y (0–12/13–15/16–40) and 4–17y (0–13/14–16/17–40).") +
  theme_classic()
p_SDQ_cutoffs_S1
```

```
ggsave("AppendixS1_Figure_SDQ_cutoffs.png", p_SDQ_cutoffs_S1, width = 7, height = 4.5, dpi = 300)
```

Age-adjusted SDQ Total Difficulties categories
(Normal/Borderline/Abnormal) and elevated proportion
(Borderline+Abnormal) by group


| Group | N | Normal\_n | Borderline\_n | Abnormal\_n | Normal\_pct | Borderline\_pct | Abnormal\_pct | Elevated\_n | Elevated\_pct |
| --- | --- | --- | --- | --- | --- | --- | --- | --- | --- |
| Functional | 66 | 36 | 6 | 24 | 54.5 | 9.1 | 36.4 | 30 | 45.5 |
| Somatic Control | 62 | 57 | 4 | 1 | 91.9 | 6.5 | 1.6 | 5 | 8.1 |
| Healthy Control | 90 | 81 | 6 | 3 | 90.0 | 6.7 | 3.3 | 9 | 10.0 |

Summary: Elevated SDQ Total (Borderline+Abnormal), age-adjusted
cut-offs.

| Functional | Somatic Control | Healthy Control |
| --- | --- | --- |
| 45.5% (30/66) | 8.1% (5/62) | 10% (9/90) |

## Plots

```
# Define colors
group_colors <- c("Functional" = "#6EC5E9", 
                  "Somatic Control" = "#F2B705", 
                  "Healthy Control" = "#1B9E77")

# --- Data in long format for plotting ---
SDQ_long <- mydata %>%
  mutate(Group_chr = str_squish(as.character(Group))) %>%
  mutate(Group_chr = str_replace_all(Group_chr, "(?i)somatic control", "Somatic Control"),
         Group_chr = str_replace_all(Group_chr, "(?i)healthy control", "Healthy Control"),
         Group_chr = str_replace_all(Group_chr, "(?i)functional",      "Functional")) %>%
  pivot_longer(starts_with("SDQ_"), names_to = "Subscale", values_to = "Value") %>%
  mutate(
    Group = factor(Group_chr, levels = c("Functional","Somatic Control","Healthy Control")),
    Subscale = factor(Subscale,
                      levels = c("SDQ_Emotional","SDQ_Conduct","SDQ_Hyperact",
                                 "SDQ_PeerProb","SDQ_Prosocial","SDQ_Total"),
                      labels = c("Emotional Problems","Conduct Problems",
                                 "Hyperactivity","Peer Problems",
                                 "Prosocial Behaviour","Total Difficulties")),
    y_max = ifelse(Subscale == "Total Difficulties", 40, 10)
  ) %>%
  filter(!is.na(Group), !is.na(Value))   

# --- Boxplot + Jitter ---
Fig_Box <- ggplot(SDQ_long, aes(Group, Value)) +
  geom_blank(aes(y = 0)) +
  geom_blank(aes(y = y_max)) +
  geom_boxplot(width = 0.35, fill = "white", color = "black",
               outlier.shape = NA, linewidth = 0.4, na.rm = TRUE) +
  geom_jitter(aes(color = Group), width = 0.12, alpha = 0.5, size = 1.2,
              na.rm = TRUE) +
  facet_wrap(~ Subscale, scales = "free_y", ncol = 3) +
  scale_color_manual(values = group_colors) +
  labs(x = NULL, y = NULL) +   # <<< y-axis title removed
  theme_minimal(base_size = 13) +
  theme(
    panel.background = element_rect(fill = "white", color = NA),
    plot.background  = element_rect(fill = "white", color = NA),
    panel.grid.major.y = element_line(color = "grey90", linewidth = 0.3),
    panel.grid.minor   = element_blank(),
    axis.text.x = element_blank(),
    axis.title.x = element_blank(),
    axis.text.y = element_text(size = 10),
    strip.text  = element_text(face = "bold", size = 12, color = "black"),
    legend.position = "bottom",
    legend.title = element_blank(),
    legend.text  = element_text(size = 11),
    plot.margin  = margin(5,10,5,10))
print(Fig_Box)
```

```
# --- Export as TIFF ---
Fig_Box <- Fig_Box + theme(plot.background = element_rect(fill = "white", color = NA))
if (!knitr::is_html_output()) {
  ggsave("Figure1_SDQ_Boxplot.tiff",
         Fig_Box,
         width = 7, height = 5, dpi = 600,
         compression = "lzw")}

# --- Density plot (smooth version of histograms) ---
# Figure S1: Density plots for SDQ subscales and total score
SDQ_den <- SDQ_long %>%
  mutate(x_max = ifelse(Subscale == "Total Difficulties", 40, 10))
Fig_Density <- ggplot(SDQ_den, aes(x = Value, fill = Group)) +
  geom_blank(aes(x = 0)) +
  geom_blank(aes(x = x_max)) +
  geom_density(alpha = 0.5, color = "grey20", linewidth = 0.3) +
  facet_wrap(~ Subscale, scales = "free_x", ncol = 3) +
  scale_fill_manual(values = group_colors) +
  labs(
    x = "SDQ Score",
    y = "Density"
  ) +
  theme_minimal(base_size = 13) +
  theme(
    panel.grid = element_blank(),
    axis.text.x = element_text(size = 10),
    axis.text.y = element_text(size = 10),
    strip.text = element_text(face = "bold", size = 12, color = "black"),
    legend.position = "bottom",
    legend.title = element_blank(),
    legend.text = element_text(size = 11),
    plot.margin = margin(5, 10, 5, 10))
Fig_Density
```

```
# Figure S1 Density distributions of SDQ total and subscale scores by group (Functional, Somatic Control, Healthy Control). Subscales range 0–10; Total score 0–40.
```

## Calculation of crohnbach alpha for all Groups and SDQ Subscales

```
# Define variables
SDQ_variables <- c("SDQ_Emotional", "SDQ_Conduct", "SDQ_Hyperact", "SDQ_PeerProb", "SDQ_Prosocial", "SDQ_Total")

# Function to compute alpha per group
get_alpha <- function(data, group_name) {
  data_group <- data[data$Group == group_name, SDQ_variables]
  alpha_result <- psych::alpha(data_group, check.keys = TRUE)
  cat("\n--- Cronbach's Alpha for", group_name, "---\n")
  print(alpha_result$total$raw_alpha)
  return(alpha_result$total$raw_alpha)}

# Calculate for each group
alpha_functional <- get_alpha(mydata, "Functional")       # ~0.78
alpha_somatic    <- get_alpha(mydata, "Somatic Control")  # ~0.81
alpha_healthy    <- get_alpha(mydata, "Healthy Control")  # ~0.79

# Calculate for full sample
cat("\n--- Cronbach's Alpha for All Groups Combined ---\n")
alpha_all <- psych::alpha(mydata[, SDQ_variables], check.keys = TRUE)
print(alpha_all$total$raw_alpha)  # ~0.82

# Summary table for reference
cronbach_summary <- data.frame(
  Group = c("Functional", "Somatic Control", "Healthy Control", "All Groups"),
  Raw_Alpha = c(alpha_functional, alpha_somatic, alpha_healthy, alpha_all$total$raw_alpha))
print(cronbach_summary)

# Interpretation
cat("\nIn our study, Cronbach’s alpha for the SDQ was", 
    round(alpha_all$total$raw_alpha, 2), 
    ", indicating good internal consistency (≥0.8).\n")
cat("All SDQ subscales showed acceptable to good reliability (≥0.7),",
    "with only one value slightly below 0.7 in the Functional group for SDQ_Total.\n")
```

```
## 
## --- Cronbach's Alpha for Functional ---
## [1] 0.7822279
## 
## --- Cronbach's Alpha for Somatic Control ---
## [1] 0.8124338
## 
## --- Cronbach's Alpha for Healthy Control ---
## [1] 0.7932974
## 
## --- Cronbach's Alpha for All Groups Combined ---
## [1] 0.8194009
##             Group Raw_Alpha
## 1      Functional 0.7822279
## 2 Somatic Control 0.8124338
## 3 Healthy Control 0.7932974
## 4      All Groups 0.8194009
## 
## In our study, Cronbach’s alpha for the SDQ was 0.82 , indicating good internal consistency (≥0.8).
## All SDQ subscales showed acceptable to good reliability (≥0.7), with only one value slightly below 0.7 in the Functional group for SDQ_Total.
```

## Testing Normal distribution and homogenity of variance

```
# --- Shapiro–Wilk Test per Group and SDQ Variable ---
SDQ_shapiro <- map_df(SDQ_variables, ~ {
  result <- mydata %>%
    group_by(Group) %>%
    summarise(
      Shapiro_p = shapiro.test(.data[[.x]])$p.value,  
      Shapiro_W = shapiro.test(.data[[.x]])$statistic,  
      .groups = "drop")
  result$Variable <- .x  
  return(result)})
knitr::kable(SDQ_shapiro, caption = "Shapiro–Wilk test for normality of SDQ subscores by group")

# Interpretation: All variables show p < 0.05 → non-normal distribution → use Kruskal–Wallis tests.

# --- Levene’s Test for Homogeneity of Variance ---
SDQ_levene <- lapply(SDQ_variables, function(subscore) {
  test <- leveneTest(mydata[[subscore]] ~ mydata$Group)
  data.frame(Variable = subscore,
    F_Value = test$`F value`[1],
    P_Value = test$`Pr(>F)`[1] )})
knitr::kable(SDQ_levene, caption = "Levene’s test for homogeneity of variance across groups")

# Interpretation: Most subscores show p < 0.05 → variances are not homogeneous → non-parametric tests appropriate
```

Shapiro–Wilk test for normality of SDQ subscores by
group

| Group | Shapiro\_p | Shapiro\_W | Variable |
| --- | --- | --- | --- |
| Functional | 0.0251241 | 0.9579499 | SDQ\_Emotional |
| Somatic Control | 0.0000000 | 0.7289520 | SDQ\_Emotional |
| Healthy Control | 0.0000000 | 0.8188614 | SDQ\_Emotional |
| Functional | 0.0000219 | 0.8877443 | SDQ\_Conduct |
| Somatic Control | 0.0000000 | 0.6519406 | SDQ\_Conduct |
| Healthy Control | 0.0000001 | 0.8557322 | SDQ\_Conduct |
| Functional | 0.0101283 | 0.9502664 | SDQ\_Hyperact |
| Somatic Control | 0.0006965 | 0.9212831 | SDQ\_Hyperact |
| Healthy Control | 0.0000050 | 0.9020059 | SDQ\_Hyperact |
| Functional | 0.0000021 | 0.8575873 | SDQ\_PeerProb |
| Somatic Control | 0.0000000 | 0.5628329 | SDQ\_PeerProb |
| Healthy Control | 0.0000000 | 0.7633380 | SDQ\_PeerProb |
| Functional | 0.0008115 | 0.9270539 | SDQ\_Prosocial |
| Somatic Control | 0.0000000 | 0.4975388 | SDQ\_Prosocial |
| Healthy Control | 0.0000002 | 0.8678620 | SDQ\_Prosocial |
| Functional | 0.0783847 | 0.9672512 | SDQ\_Total |
| Somatic Control | 0.0000039 | 0.8580812 | SDQ\_Total |
| Healthy Control | 0.0005184 | 0.9414961 | SDQ\_Total |

Levene’s test for homogeneity of variance across groups

| | Variable | F\_Value | P\_Value | | --- | --- | --- | | SDQ\_Emotional | 13.36059 | 3.4e-06 | | | Variable | F\_Value | P\_Value | | --- | --- | --- | | SDQ\_Conduct | 11.70435 | 1.5e-05 | | | Variable | F\_Value | P\_Value | | --- | --- | --- | | SDQ\_Hyperact | 5.098433 | 0.0068657 | | | Variable | F\_Value | P\_Value | | --- | --- | --- | | SDQ\_PeerProb | 19.54998 | 0 | | | Variable | F\_Value | P\_Value | | --- | --- | --- | | SDQ\_Prosocial | 24.86909 | 0 | | | Variable | F\_Value | P\_Value | | --- | --- | --- | | SDQ\_Total | 7.251663 | 0.000896 | |

## Non-Parametric analysis for SDQ\_Total and Subscales

### Kruskal, Dunn post-hoc and Cliff’s delta effect size

```
# --- Kruskal–Wallis test for each SDQ variable ---
SDQ_kruskal <- lapply(SDQ_variables, function(var) {
  test <- kruskal.test(as.formula(paste(var, "~ Group")), data = mydata)
  data.frame(
    Variable = var,
    Chi_Square = round(test$statistic, 3),
    df = test$parameter,
    p_value = round(test$p.value, 5))})
SDQ_kruskal_df <- do.call(rbind, SDQ_kruskal)
cat("\n--- Kruskal-Wallis Tests for SDQ Scores ---\n")
print(SDQ_kruskal_df)

# --- Post-hoc Dunn tests (Bonferroni-adjusted) ---
SDQ_dunn <- lapply(SDQ_variables, function(var) {
  FSA::dunnTest(as.formula(paste(var, "~ Group")), data = mydata, method = "bonferroni")$res})
names(SDQ_dunn) <- SDQ_variables
SDQ_dunn_table <- do.call(rbind, Map(cbind, SDQ_dunn, Variable = names(SDQ_dunn)))
cat("\n--- Dunn Post-hoc Tests (Bonferroni adjusted) ---\n")
print(SDQ_dunn_table)

# --- Effect-size with Cliffs Delta---
cliffs_delta_results <- list()
for (var in SDQ_variables) {
  comparisons <- list(
    c("Functional", "Somatic Control"),
    c("Functional", "Healthy Control"),
    c("Healthy Control", "Somatic Control"))
  for (comp in comparisons) {
    group1 <- comp[1]
    group2 <- comp[2]
    result <- cliff.delta(mydata[[var]][mydata$Group == group1], 
                          mydata[[var]][mydata$Group == group2])
    cliffs_delta_results[[paste(var, group1, group2, sep = "_")]] <- data.frame(
      Variable = var,
      Group_1 = group1,
      Group_2 = group2,
      Cliff_Delta = round(result$estimate, 2), 
      CI_Lower = round(result$conf.int[1], 2),  
      CI_Upper = round(result$conf.int[2], 2), 
      Effect_Size = case_when(
        result$estimate > 0.70 ~ "Very large",
        result$estimate > 0.50 ~ "Large",
        result$estimate > 0.30 ~ "Medium",
        result$estimate > 0  ~ "Small",
        result$estimate < -0.70 ~ "Very large negative", 
        result$estimate < -0.50 ~ "Large negative",
        result$estimate < -0.30 ~ "Medium negative",
        TRUE ~ "Small negative"))}}
cliffs_delta_df <- do.call(rbind, cliffs_delta_results)
cat("\n--- Cliff’s Delta Effect Sizes ---\n")
print(cliffs_delta_df)

cat("\nInterpretation:\n")
cat("Kruskal-Wallis tests show significant group differences for all SDQ subscales (all p < .05).\n")
cat("Post-hoc Dunn tests confirm that the Functional group consistently scores higher than both control groups.\n")
cat("Cliff’s Delta indicates small to large effect sizes, strongest between Functional and Healthy Control groups.\n")
```

```
## 
## --- Kruskal-Wallis Tests for SDQ Scores ---
##                                  Variable Chi_Square df p_value
## Kruskal-Wallis chi-squared  SDQ_Emotional     66.030  2   0e+00
## Kruskal-Wallis chi-squared1   SDQ_Conduct     30.945  2   0e+00
## Kruskal-Wallis chi-squared2  SDQ_Hyperact     21.393  2   2e-05
## Kruskal-Wallis chi-squared3  SDQ_PeerProb     34.601  2   0e+00
## Kruskal-Wallis chi-squared4 SDQ_Prosocial     59.269  2   0e+00
## Kruskal-Wallis chi-squared5     SDQ_Total     63.883  2   0e+00
## 
## --- Dunn Post-hoc Tests (Bonferroni adjusted) ---
##                                        Comparison          Z      P.unadj
## SDQ_Emotional.1      Functional - Healthy Control  7.3283919 2.329307e-13
## SDQ_Emotional.2      Functional - Somatic Control  6.8539877 7.181924e-12
## SDQ_Emotional.3 Healthy Control - Somatic Control  0.1490291 8.815307e-01
## SDQ_Conduct.1        Functional - Healthy Control  3.0842752 2.040487e-03
## SDQ_Conduct.2        Functional - Somatic Control  5.5601575 2.695312e-08
## SDQ_Conduct.3   Healthy Control - Somatic Control  2.9298319 3.391454e-03
## SDQ_Hyperact.1       Functional - Healthy Control  3.8102228 1.388416e-04
## SDQ_Hyperact.2       Functional - Somatic Control  4.2400068 2.235130e-05
## SDQ_Hyperact.3  Healthy Control - Somatic Control  0.8023559 4.223471e-01
## SDQ_PeerProb.1       Functional - Healthy Control  3.3871251 7.062916e-04
## SDQ_PeerProb.2       Functional - Somatic Control  5.8727935 4.285122e-09
## SDQ_PeerProb.3  Healthy Control - Somatic Control  2.9674860 3.002459e-03
## SDQ_Prosocial.1      Functional - Healthy Control -2.1096676 3.488700e-02
## SDQ_Prosocial.2      Functional - Somatic Control -7.4001167 1.360649e-13
## SDQ_Prosocial.3 Healthy Control - Somatic Control -5.8584921 4.670889e-09
## SDQ_Total.1          Functional - Healthy Control  6.2385534 4.416360e-10
## SDQ_Total.2          Functional - Somatic Control  7.5390175 4.735255e-14
## SDQ_Total.3     Healthy Control - Somatic Control  1.9532120 5.079449e-02
##                        P.adj      Variable
## SDQ_Emotional.1 6.987921e-13 SDQ_Emotional
## SDQ_Emotional.2 2.154577e-11 SDQ_Emotional
## SDQ_Emotional.3 1.000000e+00 SDQ_Emotional
## SDQ_Conduct.1   6.121462e-03   SDQ_Conduct
## SDQ_Conduct.2   8.085937e-08   SDQ_Conduct
## SDQ_Conduct.3   1.017436e-02   SDQ_Conduct
## SDQ_Hyperact.1  4.165248e-04  SDQ_Hyperact
## SDQ_Hyperact.2  6.705390e-05  SDQ_Hyperact
## SDQ_Hyperact.3  1.000000e+00  SDQ_Hyperact
## SDQ_PeerProb.1  2.118875e-03  SDQ_PeerProb
## SDQ_PeerProb.2  1.285537e-08  SDQ_PeerProb
## SDQ_PeerProb.3  9.007378e-03  SDQ_PeerProb
## SDQ_Prosocial.1 1.046610e-01 SDQ_Prosocial
## SDQ_Prosocial.2 4.081946e-13 SDQ_Prosocial
## SDQ_Prosocial.3 1.401267e-08 SDQ_Prosocial
## SDQ_Total.1     1.324908e-09     SDQ_Total
## SDQ_Total.2     1.420577e-13     SDQ_Total
## SDQ_Total.3     1.523835e-01     SDQ_Total
## 
## --- Cliff’s Delta Effect Sizes ---
##                                                    Variable         Group_1
## SDQ_Emotional_Functional_Somatic Control      SDQ_Emotional      Functional
## SDQ_Emotional_Functional_Healthy Control      SDQ_Emotional      Functional
## SDQ_Emotional_Healthy Control_Somatic Control SDQ_Emotional Healthy Control
## SDQ_Conduct_Functional_Somatic Control          SDQ_Conduct      Functional
## SDQ_Conduct_Functional_Healthy Control          SDQ_Conduct      Functional
## SDQ_Conduct_Healthy Control_Somatic Control     SDQ_Conduct Healthy Control
## SDQ_Hyperact_Functional_Somatic Control        SDQ_Hyperact      Functional
## SDQ_Hyperact_Functional_Healthy Control        SDQ_Hyperact      Functional
## SDQ_Hyperact_Healthy Control_Somatic Control   SDQ_Hyperact Healthy Control
## SDQ_PeerProb_Functional_Somatic Control        SDQ_PeerProb      Functional
## SDQ_PeerProb_Functional_Healthy Control        SDQ_PeerProb      Functional
## SDQ_PeerProb_Healthy Control_Somatic Control   SDQ_PeerProb Healthy Control
## SDQ_Prosocial_Functional_Somatic Control      SDQ_Prosocial      Functional
## SDQ_Prosocial_Functional_Healthy Control      SDQ_Prosocial      Functional
## SDQ_Prosocial_Healthy Control_Somatic Control SDQ_Prosocial Healthy Control
## SDQ_Total_Functional_Somatic Control              SDQ_Total      Functional
## SDQ_Total_Functional_Healthy Control              SDQ_Total      Functional
## SDQ_Total_Healthy Control_Somatic Control         SDQ_Total Healthy Control
##                                                       Group_2 Cliff_Delta
## SDQ_Emotional_Functional_Somatic Control      Somatic Control        0.65
## SDQ_Emotional_Functional_Healthy Control      Healthy Control        0.69
## SDQ_Emotional_Healthy Control_Somatic Control Somatic Control        0.04
## SDQ_Conduct_Functional_Somatic Control        Somatic Control        0.52
## SDQ_Conduct_Functional_Healthy Control        Healthy Control        0.30
## SDQ_Conduct_Healthy Control_Somatic Control   Somatic Control        0.29
## SDQ_Hyperact_Functional_Somatic Control       Somatic Control        0.44
## SDQ_Hyperact_Functional_Healthy Control       Healthy Control        0.35
## SDQ_Hyperact_Healthy Control_Somatic Control  Somatic Control        0.07
## SDQ_PeerProb_Functional_Somatic Control       Somatic Control        0.54
## SDQ_PeerProb_Functional_Healthy Control       Healthy Control        0.31
## SDQ_PeerProb_Healthy Control_Somatic Control  Somatic Control        0.28
## SDQ_Prosocial_Functional_Somatic Control      Somatic Control       -0.66
## SDQ_Prosocial_Functional_Healthy Control      Healthy Control       -0.25
## SDQ_Prosocial_Healthy Control_Somatic Control Somatic Control       -0.60
## SDQ_Total_Functional_Somatic Control          Somatic Control        0.72
## SDQ_Total_Functional_Healthy Control          Healthy Control        0.62
## SDQ_Total_Healthy Control_Somatic Control     Somatic Control        0.22
##                                               CI_Lower CI_Upper    Effect_Size
## SDQ_Emotional_Functional_Somatic Control          0.48     0.77          Large
## SDQ_Emotional_Functional_Healthy Control          0.55     0.80          Large
## SDQ_Emotional_Healthy Control_Somatic Control    -0.14     0.21          Small
## SDQ_Conduct_Functional_Somatic Control            0.34     0.66          Large
## SDQ_Conduct_Functional_Healthy Control            0.12     0.47         Medium
## SDQ_Conduct_Healthy Control_Somatic Control       0.11     0.46          Small
## SDQ_Hyperact_Functional_Somatic Control           0.25     0.60         Medium
## SDQ_Hyperact_Functional_Healthy Control           0.17     0.51         Medium
## SDQ_Hyperact_Healthy Control_Somatic Control     -0.11     0.25          Small
## SDQ_PeerProb_Functional_Somatic Control           0.37     0.67          Large
## SDQ_PeerProb_Functional_Healthy Control           0.13     0.47         Medium
## SDQ_PeerProb_Healthy Control_Somatic Control      0.12     0.42          Small
## SDQ_Prosocial_Functional_Somatic Control         -0.77    -0.50 Large negative
## SDQ_Prosocial_Functional_Healthy Control         -0.42    -0.06 Small negative
## SDQ_Prosocial_Healthy Control_Somatic Control    -0.72    -0.44 Large negative
## SDQ_Total_Functional_Somatic Control              0.57     0.83     Very large
## SDQ_Total_Functional_Healthy Control              0.46     0.73          Large
## SDQ_Total_Healthy Control_Somatic Control         0.03     0.40          Small
## 
## Interpretation:
## Kruskal-Wallis tests show significant group differences for all SDQ subscales (all p < .05).
## Post-hoc Dunn tests confirm that the Functional group consistently scores higher than both control groups.
## Cliff’s Delta indicates small to large effect sizes, strongest between Functional and Healthy Control groups.
```

## Model for SDQ\_Total 1. —

```
cat("\n--- Negative Binomial Models ---\n")

# Simple negative binomial model
SDQ_nb_simple <- glm.nb(SDQ_Total ~ Group, data = mydata)
cat("\nModel: SDQ_Total ~ Group\n")
print(summary(SDQ_nb_simple))

# Main model with confounders
SDQ_nb <- glm.nb(SDQ_Total ~ Group + ChildGender + ChildAge + ParentsSeparated + 
                   Parent1_Education_level_num + PHQ_Total, data = mydata)
cat("\nModel: SDQ_Total ~ Group + Gender + Age + Separation + Education + PHQ_Total\n")
print(summary(SDQ_nb))

# Alternative models for sensitivity
SDQ_nb1 <- glm.nb(SDQ_Total ~ Group + ChildGender + ChildAge + ParentsSeparated + Parent1 + Parent1_Age, data = mydata)
cat("\nModel SDQ_nb1 summary:\n"); print(summary(SDQ_nb1))

SDQ_nb2 <- glm.nb(SDQ_Total ~ Group + ChildGender + ChildAge + ParentsSeparated + 
                    Parent1 + Parent1_Age + Parent1_Education_level_num + 
                    Parent2_Education_level_num, data = mydata)
cat("\nModel SDQ_nb2 summary:\n"); print(summary(SDQ_nb2))

SDQ_nb3 <- glm.nb(SDQ_Total ~ Group + ChildGender + ChildAge + ParentsSeparated + 
                    Parent1 + Parent1_Age + Parent1_Education_level_num + 
                    Parent2_Education_level_num + PHQ_Total, data = mydata)
cat("\nModel SDQ_nb3 summary:\n"); print(summary(SDQ_nb3))

# Model comparison using AIC
cat("\n--- Model Comparison (AIC) ---\n")
model_comp <- AIC(SDQ_nb_simple, SDQ_nb, SDQ_nb1, SDQ_nb2, SDQ_nb3)
print(model_comp)

cat("\n--- Poisson Model (for comparison) ---\n")
mean_val <- mean(mydata$SDQ_Total)
var_val  <- var(mydata$SDQ_Total)
cat("Mean:", round(mean_val, 2), " | Variance:", round(var_val, 2), "\n")

# Overdispersion check: var > mean → Poisson not ideal
if (var_val > mean_val) cat("→ Overdispersion detected: Negative Binomial preferred.\n")
ggplot(mydata, aes(x = SDQ_Total, fill = Group)) +  
  geom_histogram(binwidth = 1, position = "dodge", alpha = 0.6, color = "black") +  
  labs(x = "SDQ Total Score", y = "Count") + 
  theme_minimal() + 
  scale_fill_manual(values = c("skyblue", "orange", "green"))
```

```
# Poisson GLM as reference
SDQ_glm <- glm(SDQ_Total ~ Group + ChildGender + ChildAge + ParentsSeparated + 
                 Parent1_Education_level_num + PHQ_Total, 
               family = poisson(link = "log"), data = mydata)
cat("\nModel: Poisson (GLM) Summary\n")
print(summary(SDQ_glm))

# Compare model fit with likelihood ratio (drop1)
cat("\n--- Drop1 Test (Model Comparison) ---\n")
cat("\nPoisson model:\n"); print(drop1(SDQ_glm, test = "Chisq"))
cat("\nNegative Binomial model:\n"); print(drop1(SDQ_nb, test = "Chisq"))

cat("\nInterpretation:\n")
cat("The Negative Binomial model fits the data better (lower AIC, handles overdispersion).\n")
cat("Group remains a significant predictor of SDQ_Total even after adjusting for confounders.\n")
cat("Covariates (child age, gender, separation, parental PHQ) show significant influence as expected.\n")
```

```
## 
## --- Negative Binomial Models ---
## 
## Model: SDQ_Total ~ Group
## 
## Call:
## glm.nb(formula = SDQ_Total ~ Group, data = mydata, init.theta = 2.580593367, 
##     link = log)
## 
## Coefficients:
##                      Estimate Std. Error z value Pr(>|z|)    
## (Intercept)           2.60829    0.08359  31.203  < 2e-16 ***
## GroupSomatic Control -0.99563    0.12827  -7.762 8.36e-15 ***
## GroupHealthy Control -0.73307    0.11400  -6.430 1.27e-10 ***
## ---
## Signif. codes:  0 '***' 0.001 '**' 0.01 '*' 0.05 '.' 0.1 ' ' 1
## 
## (Dispersion parameter for Negative Binomial(2.5806) family taken to be 1)
## 
##     Null deviance: 325.95  on 217  degrees of freedom
## Residual deviance: 252.85  on 215  degrees of freedom
## AIC: 1307.1
## 
## Number of Fisher Scoring iterations: 1
## 
## 
##               Theta:  2.581 
##           Std. Err.:  0.363 
## 
##  2 x log-likelihood:  -1299.056 
## 
## Model: SDQ_Total ~ Group + Gender + Age + Separation + Education + PHQ_Total
## 
## Call:
## glm.nb(formula = SDQ_Total ~ Group + ChildGender + ChildAge + 
##     ParentsSeparated + Parent1_Education_level_num + PHQ_Total, 
##     data = mydata, init.theta = 3.561767479, link = log)
## 
## Coefficients:
##                             Estimate Std. Error z value Pr(>|z|)    
## (Intercept)                  2.39445    0.18758  12.765  < 2e-16 ***
## GroupSomatic Control        -0.71397    0.12538  -5.694 1.24e-08 ***
## GroupHealthy Control        -0.50131    0.11062  -4.532 5.84e-06 ***
## ChildGender                 -0.24553    0.09005  -2.726   0.0064 ** 
## ChildAge                    -0.03978    0.01580  -2.519   0.0118 *  
## ParentsSeparated             0.21254    0.10851   1.959   0.0501 .  
## Parent1_Education_level_num  0.11113    0.08115   1.369   0.1709    
## PHQ_Total                    0.11013    0.02056   5.357 8.47e-08 ***
## ---
## Signif. codes:  0 '***' 0.001 '**' 0.01 '*' 0.05 '.' 0.1 ' ' 1
## 
## (Dispersion parameter for Negative Binomial(3.5618) family taken to be 1)
## 
##     Null deviance: 393.80  on 214  degrees of freedom
## Residual deviance: 253.67  on 207  degrees of freedom
##   (3 observations deleted due to missingness)
## AIC: 1254.5
## 
## Number of Fisher Scoring iterations: 1
## 
## 
##               Theta:  3.562 
##           Std. Err.:  0.575 
## 
##  2 x log-likelihood:  -1236.544 
## 
## Model SDQ_nb1 summary:
## 
## Call:
## glm.nb(formula = SDQ_Total ~ Group + ChildGender + ChildAge + 
##     ParentsSeparated + Parent1 + Parent1_Age, data = mydata, 
##     init.theta = 2.895051302, link = log)
## 
## Coefficients:
##                      Estimate Std. Error z value Pr(>|z|)    
## (Intercept)           2.45387    0.35752   6.864 6.71e-12 ***
## GroupSomatic Control -0.96860    0.13765  -7.037 1.97e-12 ***
## GroupHealthy Control -0.67918    0.11778  -5.767 8.09e-09 ***
## ChildGender          -0.21666    0.10157  -2.133  0.03292 *  
## ChildAge             -0.05560    0.02034  -2.733  0.00628 ** 
## ParentsSeparated      0.30154    0.11976   2.518  0.01180 *  
## Parent1              -0.12075    0.11728  -1.030  0.30319    
## Parent1_Age           0.01312    0.01003   1.308  0.19088    
## ---
## Signif. codes:  0 '***' 0.001 '**' 0.01 '*' 0.05 '.' 0.1 ' ' 1
## 
## (Dispersion parameter for Negative Binomial(2.8951) family taken to be 1)
## 
##     Null deviance: 333.75  on 199  degrees of freedom
## Residual deviance: 233.81  on 192  degrees of freedom
##   (18 observations deleted due to missingness)
## AIC: 1181.3
## 
## Number of Fisher Scoring iterations: 1
## 
## 
##               Theta:  2.895 
##           Std. Err.:  0.451 
## 
##  2 x log-likelihood:  -1163.326 
## 
## Model SDQ_nb2 summary:
## 
## Call:
## glm.nb(formula = SDQ_Total ~ Group + ChildGender + ChildAge + 
##     ParentsSeparated + Parent1 + Parent1_Age + Parent1_Education_level_num + 
##     Parent2_Education_level_num, data = mydata, init.theta = 3.062605816, 
##     link = log)
## 
## Coefficients:
##                              Estimate Std. Error z value Pr(>|z|)    
## (Intercept)                  2.211865   0.400735   5.520 3.40e-08 ***
## GroupSomatic Control        -0.955608   0.137403  -6.955 3.53e-12 ***
## GroupHealthy Control        -0.650842   0.119351  -5.453 4.95e-08 ***
## ChildGender                 -0.257297   0.101653  -2.531 0.011370 *  
## ChildAge                    -0.066964   0.020346  -3.291 0.000997 ***
## ParentsSeparated             0.196237   0.122341   1.604 0.108709    
## Parent1                     -0.101220   0.117332  -0.863 0.388312    
## Parent1_Age                  0.013858   0.010339   1.340 0.180147    
## Parent1_Education_level_num -0.002548   0.108918  -0.023 0.981335    
## Parent2_Education_level_num  0.225602   0.088243   2.557 0.010570 *  
## ---
## Signif. codes:  0 '***' 0.001 '**' 0.01 '*' 0.05 '.' 0.1 ' ' 1
## 
## (Dispersion parameter for Negative Binomial(3.0626) family taken to be 1)
## 
##     Null deviance: 337.79  on 196  degrees of freedom
## Residual deviance: 230.27  on 187  degrees of freedom
##   (21 observations deleted due to missingness)
## AIC: 1155.8
## 
## Number of Fisher Scoring iterations: 1
## 
## 
##               Theta:  3.063 
##           Std. Err.:  0.491 
## 
##  2 x log-likelihood:  -1133.773 
## 
## Model SDQ_nb3 summary:
## 
## Call:
## glm.nb(formula = SDQ_Total ~ Group + ChildGender + ChildAge + 
##     ParentsSeparated + Parent1 + Parent1_Age + Parent1_Education_level_num + 
##     Parent2_Education_level_num + PHQ_Total, data = mydata, init.theta = 3.973946438, 
##     link = log)
## 
## Coefficients:
##                              Estimate Std. Error z value Pr(>|z|)    
## (Intercept)                  2.104421   0.372198   5.654 1.57e-08 ***
## GroupSomatic Control        -0.762380   0.132025  -5.774 7.72e-09 ***
## GroupHealthy Control        -0.498408   0.112621  -4.426 9.62e-06 ***
## ChildGender                 -0.297866   0.093943  -3.171  0.00152 ** 
## ChildAge                    -0.056536   0.018954  -2.983  0.00286 ** 
## ParentsSeparated             0.154186   0.113100   1.363  0.17280    
## Parent1                     -0.089861   0.108864  -0.825  0.40912    
## Parent1_Age                  0.008125   0.009631   0.844  0.39886    
## Parent1_Education_level_num  0.008819   0.099928   0.088  0.92967    
## Parent2_Education_level_num  0.154912   0.082045   1.888  0.05901 .  
## PHQ_Total                    0.122016   0.020702   5.894 3.77e-09 ***
## ---
## Signif. codes:  0 '***' 0.001 '**' 0.01 '*' 0.05 '.' 0.1 ' ' 1
## 
## (Dispersion parameter for Negative Binomial(3.9739) family taken to be 1)
## 
##     Null deviance: 392.78  on 196  degrees of freedom
## Residual deviance: 232.49  on 186  degrees of freedom
##   (21 observations deleted due to missingness)
## AIC: 1128.3
## 
## Number of Fisher Scoring iterations: 1
## 
## 
##               Theta:  3.974 
##           Std. Err.:  0.705 
## 
##  2 x log-likelihood:  -1104.299 
## 
## --- Model Comparison (AIC) ---
##               df      AIC
## SDQ_nb_simple  4 1307.056
## SDQ_nb         9 1254.544
## SDQ_nb1        9 1181.326
## SDQ_nb2       11 1155.773
## SDQ_nb3       12 1128.299
## 
## --- Poisson Model (for comparison) ---
## Mean: 8.23  | Variance: 41.19 
## → Overdispersion detected: Negative Binomial preferred.
## 
## Model: Poisson (GLM) Summary
## 
## Call:
## glm(formula = SDQ_Total ~ Group + ChildGender + ChildAge + ParentsSeparated + 
##     Parent1_Education_level_num + PHQ_Total, family = poisson(link = "log"), 
##     data = mydata)
## 
## Coefficients:
##                              Estimate Std. Error z value Pr(>|z|)    
## (Intercept)                  2.409181   0.096880  24.868  < 2e-16 ***
## GroupSomatic Control        -0.719022   0.070995 -10.128  < 2e-16 ***
## GroupHealthy Control        -0.515240   0.058304  -8.837  < 2e-16 ***
## ChildGender                 -0.262623   0.048846  -5.377 7.59e-08 ***
## ChildAge                    -0.031986   0.008472  -3.775  0.00016 ***
## ParentsSeparated             0.224349   0.054961   4.082 4.47e-05 ***
## Parent1_Education_level_num  0.096639   0.041626   2.322  0.02025 *  
## PHQ_Total                    0.092479   0.009565   9.668  < 2e-16 ***
## ---
## Signif. codes:  0 '***' 0.001 '**' 0.01 '*' 0.05 '.' 0.1 ' ' 1
## 
## (Dispersion parameter for poisson family taken to be 1)
## 
##     Null deviance: 1107.8  on 214  degrees of freedom
## Residual deviance:  644.1  on 207  degrees of freedom
##   (3 observations deleted due to missingness)
## AIC: 1412.8
## 
## Number of Fisher Scoring iterations: 5
## 
## 
## --- Drop1 Test (Model Comparison) ---
## 
## Poisson model:
## Single term deletions
## 
## Model:
## SDQ_Total ~ Group + ChildGender + ChildAge + ParentsSeparated + 
##     Parent1_Education_level_num + PHQ_Total
##                             Df Deviance    AIC     LRT  Pr(>Chi)    
## <none>                           644.10 1412.8                      
## Group                        2   773.09 1537.8 128.986 < 2.2e-16 ***
## ChildGender                  1   673.37 1440.1  29.266 6.310e-08 ***
## ChildAge                     1   658.41 1425.1  14.311 0.0001549 ***
## ParentsSeparated             1   660.35 1427.1  16.253 5.542e-05 ***
## Parent1_Education_level_num  1   649.40 1416.1   5.303 0.0212846 *  
## PHQ_Total                    1   729.71 1496.4  85.613 < 2.2e-16 ***
## ---
## Signif. codes:  0 '***' 0.001 '**' 0.01 '*' 0.05 '.' 0.1 ' ' 1
## 
## Negative Binomial model:
## Single term deletions
## 
## Model:
## SDQ_Total ~ Group + ChildGender + ChildAge + ParentsSeparated + 
##     Parent1_Education_level_num + PHQ_Total
##                             Df Deviance    AIC    LRT  Pr(>Chi)    
## <none>                           253.67 1252.5                     
## Group                        2   290.83 1285.7 37.158 8.535e-09 ***
## ChildGender                  1   261.04 1257.9  7.364  0.006654 ** 
## ChildAge                     1   259.78 1256.7  6.109  0.013451 *  
## ParentsSeparated             1   257.49 1254.4  3.818  0.050713 .  
## Parent1_Education_level_num  1   255.54 1252.4  1.872  0.171252    
## PHQ_Total                    1   281.61 1278.5 27.936 1.254e-07 ***
## ---
## Signif. codes:  0 '***' 0.001 '**' 0.01 '*' 0.05 '.' 0.1 ' ' 1
## 
## Interpretation:
## The Negative Binomial model fits the data better (lower AIC, handles overdispersion).
## Group remains a significant predictor of SDQ_Total even after adjusting for confounders.
## Covariates (child age, gender, separation, parental PHQ) show significant influence as expected.
```

### Residuals, Outliers and Multicollinearity

```
cat("\n--- Multicollinearity Check (VIF) ---\n")
vif_values <- vif(SDQ_nb)
print(vif_values)
cat("All VIF values < 2 → no relevant multicollinearity detected.\n")

cat("\n--- Residuals and Outlier Detection ---\n")
# Compute residuals and predictions
predictions <- predict(SDQ_nb, type = "response")
residuals_deviance <- residuals(SDQ_nb, type = "deviance")
# Standard deviation and thresholds (±2 SD)
residuals_sd <- sd(residuals_deviance)
upper <- mean(residuals_deviance) + 2 * residuals_sd
lower <- mean(residuals_deviance) - 2 * residuals_sd
outliers <- which(residuals_deviance > upper | residuals_deviance < lower)
cat("Number of identified outliers:", length(outliers), "\n")
# List outliers
if (length(outliers) > 0) {
  outlier_data <- data.frame(
    ID = mydata$ID_all[outliers],
    Residuals = residuals_deviance[outliers],
    Predicted = predictions[outliers])
  print(outlier_data)}

# Visualization: residuals vs predicted
plot_data <- data.frame(predicted = predictions, residuals = residuals_deviance)
ggplot(plot_data, aes(x = predicted, y = residuals)) +
  geom_point(alpha = 0.7) +
  geom_hline(yintercept = 0, color = "red", linewidth = 0.5) +
  geom_point(data = plot_data[outliers, ], 
             aes(x = predicted, y = residuals), 
             color = "red", size = 2) +
  theme_minimal() +
  labs(
    x = "Predicted values",
    y = "Deviance residuals",
    title = "Residuals vs Predicted (Negative Binomial Model)")
```

```
cat("\n--- Model Recalculation Without Outliers ---\n")
mydata_clean <- mydata[-outliers, ]
SDQ_nb_clean <- glm.nb(
  SDQ_Total ~ Group + ChildGender + ChildAge + ParentsSeparated + PHQ_Total + Parent1_Education_level_num,
  data = mydata_clean)
print(summary(SDQ_nb_clean))

# Compare AIC
aic_old <- AIC(SDQ_nb)
aic_new <- AIC(SDQ_nb_clean)
cat("\nAIC of original model: ", round(aic_old, 2),
    "\nAIC of model without outliers: ", round(aic_new, 2), "\n")

if (aic_new < aic_old) {
  cat("→ Lower AIC after removing outliers = slightly improved model fit.\n")} else {
  cat("→ AIC unchanged or higher = no improvement in model fit.\n")}

cat("\nInterpretation:\n")
cat("Excluding residual outliers (±2 SD) did not change model interpretation.\n")
cat("Negative Binomial model remains robust; results reported based on full dataset.\n")
```

```
## 
## --- Multicollinearity Check (VIF) ---
##                                 GVIF Df GVIF^(1/(2*Df))
## Group                       1.236175  2        1.054435
## ChildGender                 1.013285  1        1.006621
## ChildAge                    1.054651  1        1.026962
## ParentsSeparated            1.078478  1        1.038498
## Parent1_Education_level_num 1.072771  1        1.035747
## PHQ_Total                   1.122245  1        1.059361
## All VIF values < 2 → no relevant multicollinearity detected.
## 
## --- Residuals and Outlier Detection ---
## Number of identified outliers: 12 
##      ID Residuals Predicted
## 1     1 -2.644600  5.945532
## 74   71  2.610913  3.814997
## 93   90 -2.440163  4.654641
## 99   96 -2.723912  6.531131
## 114 111 -2.379397  4.323698
## 118 115  2.853798  5.431287
## 131 126 -2.392655  4.394045
## 146 138 -2.425432  4.572386
## 314 142 -2.550018  5.311907
## 316 144 -2.630222  5.844850
## 335 159  1.943527  5.699035
## 340 164 -2.675225  6.165476
## 
## --- Model Recalculation Without Outliers ---
## 
## Call:
## glm.nb(formula = SDQ_Total ~ Group + ChildGender + ChildAge + 
##     ParentsSeparated + PHQ_Total + Parent1_Education_level_num, 
##     data = mydata_clean, init.theta = 3.560933094, link = log)
## 
## Coefficients:
##                             Estimate Std. Error z value Pr(>|z|)    
## (Intercept)                  2.38028    0.19018  12.516  < 2e-16 ***
## GroupSomatic Control        -0.75125    0.13026  -5.767 8.06e-09 ***
## GroupHealthy Control        -0.51749    0.11519  -4.493 7.04e-06 ***
## ChildGender                 -0.24149    0.09271  -2.605  0.00919 ** 
## ChildAge                    -0.03539    0.01648  -2.148  0.03172 *  
## ParentsSeparated             0.21708    0.11049   1.965  0.04946 *  
## PHQ_Total                    0.10440    0.02196   4.754 2.00e-06 ***
## Parent1_Education_level_num  0.11832    0.08382   1.412  0.15810    
## ---
## Signif. codes:  0 '***' 0.001 '**' 0.01 '*' 0.05 '.' 0.1 ' ' 1
## 
## (Dispersion parameter for Negative Binomial(3.5609) family taken to be 1)
## 
##     Null deviance: 374.45  on 202  degrees of freedom
## Residual deviance: 239.49  on 195  degrees of freedom
##   (3 observations deleted due to missingness)
## AIC: 1188.7
## 
## Number of Fisher Scoring iterations: 1
## 
## 
##               Theta:  3.561 
##           Std. Err.:  0.591 
## 
##  2 x log-likelihood:  -1170.738 
## 
## AIC of original model:  1254.54 
## AIC of model without outliers:  1188.74 
## → Lower AIC after removing outliers = slightly improved model fit.
## 
## Interpretation:
## Excluding residual outliers (±2 SD) did not change model interpretation.
## Negative Binomial model remains robust; results reported based on full dataset.
```

### Model with pooled imputed data

```
# Fit model across all imputed datasets
SDQ_nb_imp_pool <- with(
  imp_SDQ,
  glm.nb(SDQ_Total ~ Group + ChildGender + ChildAge + ParentsSeparated + 
           Parent1_Education_level + PHQ_Total))

# Pooling across imputations 
pooled_results <- pool(SDQ_nb_imp_pool)
cat("\nPooled Model Summary (Imputed Data):\n")
print(summary(pooled_results))

# Compare with single imputed dataset (first imputation)
cat("\n--- Model on Single Imputed Dataset (Complete Imputation 1) ---\n")
SDQ_nb_imp <- glm.nb(
  SDQ_Total ~ Group + ChildGender + ChildAge + ParentsSeparated + 
    Parent1_Education_level + PHQ_Total,
  data = mydata_SDQ_impute)
print(summary(SDQ_nb_imp))

cat("\nInterpretation:\n")
cat("Results from the pooled imputed model were nearly identical to those from the original dataset.\n")
cat("No change in statistical significance or interpretation was observed after multiple imputation.\n")
cat("The primary model (SDQ_nb) is therefore considered robust to missing data.\n")
```

```
## 
## Pooled Model Summary (Imputed Data):
##                        term    estimate  std.error  statistic       df
## 1               (Intercept)  2.59933733 0.16062059 16.1830888 206.7560
## 2      GroupSomatic Control -0.71764695 0.12385240 -5.7943726 206.8407
## 3      GroupHealthy Control -0.49808114 0.10950467 -4.5484923 206.9008
## 4               ChildGender -0.24596066 0.08906498 -2.7615868 206.8860
## 5                  ChildAge -0.03732085 0.01563529 -2.3869627 206.9432
## 6          ParentsSeparated  0.22960484 0.10778801  2.1301519 206.7930
## 7 Parent1_Education_level.L  0.15972941 0.15301888  1.0438542 205.7624
## 8 Parent1_Education_level.Q -0.01519436 0.10917979 -0.1391683 206.4182
## 9                 PHQ_Total  0.10905671 0.02044903  5.3330998 206.3678
##        p.value
## 1 1.345919e-38
## 2 2.529334e-08
## 3 9.196965e-06
## 4 6.269598e-03
## 5 1.788851e-02
## 6 3.434069e-02
## 7 2.977777e-01
## 8 8.894529e-01
## 9 2.526489e-07
## 
## --- Model on Single Imputed Dataset (Complete Imputation 1) ---
## 
## Call:
## glm.nb(formula = SDQ_Total ~ Group + ChildGender + ChildAge + 
##     ParentsSeparated + Parent1_Education_level + PHQ_Total, data = mydata_SDQ_impute, 
##     init.theta = 3.601658642, link = log)
## 
## Coefficients:
##                           Estimate Std. Error z value Pr(>|z|)    
## (Intercept)                2.60030    0.16047  16.205  < 2e-16 ***
## GroupSomatic Control      -0.71865    0.12375  -5.807 6.35e-09 ***
## GroupHealthy Control      -0.49876    0.10942  -4.558 5.16e-06 ***
## ChildGender               -0.24520    0.08905  -2.754  0.00589 ** 
## ChildAge                  -0.03727    0.01563  -2.384  0.01714 *  
## ParentsSeparated           0.23094    0.10783   2.142  0.03222 *  
## Parent1_Education_level.L  0.16170    0.15265   1.059  0.28949    
## Parent1_Education_level.Q -0.01454    0.10909  -0.133  0.89395    
## PHQ_Total                  0.10890    0.02042   5.333 9.68e-08 ***
## ---
## Signif. codes:  0 '***' 0.001 '**' 0.01 '*' 0.05 '.' 0.1 ' ' 1
## 
## (Dispersion parameter for Negative Binomial(3.6017) family taken to be 1)
## 
##     Null deviance: 399.65  on 217  degrees of freedom
## Residual deviance: 257.20  on 209  degrees of freedom
## AIC: 1275.3
## 
## Number of Fisher Scoring iterations: 1
## 
## 
##               Theta:  3.602 
##           Std. Err.:  0.578 
## 
##  2 x log-likelihood:  -1255.313 
## 
## Interpretation:
## Results from the pooled imputed model were nearly identical to those from the original dataset.
## No change in statistical significance or interpretation was observed after multiple imputation.
## The primary model (SDQ_nb) is therefore considered robust to missing data.
```

### Sensitivity analyses: Interactions, age-adjusted testing

```
## --- Interaction: Group × Child Age ---
cat("\n--- Interaction Test: Group × Child Age ---\n")
SDQ_nb_age_int <- glm.nb(
  SDQ_Total ~ Group * ChildAge + ChildGender + ParentsSeparated +
    Parent1_Education_level_num + PHQ_Total,
  data = mydata)
print(summary(SDQ_nb_age_int))

cat("\nLikelihood Ratio Test (Group × Age interaction):\n")
print(anova(SDQ_nb, SDQ_nb_age_int, test = "Chisq"))
cat("\nInterpretation:\n")
cat("Non-significant Group × Age interaction indicates that group differences in SDQ_Total\n")
cat("are consistent across the age range studied.\n")

## --- Interaction: Group × Parental Separation ---
cat("\n--- Interaction Test: Group × Parental Separation ---\n")
SDQ_nb_sep_int <- glm.nb(
  SDQ_Total ~ Group * ParentsSeparated + ChildAge + ChildGender +
    Parent1_Education_level_num + PHQ_Total,
  data = mydata)
print(summary(SDQ_nb_sep_int))

cat("\nLikelihood Ratio Test (Group × Parental Separation interaction):\n")
print(anova(SDQ_nb, SDQ_nb_sep_int, test = "Chisq"))
cat("\nInterpretation:\n")
cat("Non-significant Group × Parental Separation interaction indicates that group differences\n")
cat("in SDQ_Total are not driven by family structure.\n")

## --- Interaction: Group × Child Gender ---
cat("\n--- Interaction Test: Group × Child Gender ---\n")
SDQ_nb_gender_int <- glm.nb(
  SDQ_Total ~ Group * ChildGender + ChildAge + ParentsSeparated +
    Parent1_Education_level_num + PHQ_Total,
  data = mydata)
print(summary(SDQ_nb_gender_int))

cat("\nLikelihood Ratio Test (Group × Gender interaction):\n")
print(anova(SDQ_nb, SDQ_nb_gender_int, test = "Chisq"))
cat("\nInterpretation:\n")
cat("Non-significant Group × Gender interaction suggests that group effects are similar\n")
cat("for boys and girls.\n")

## --- Interaction: Group × Index Parent Educational Level ---
cat("\n--- Interaction Test: Group × Index Parent Educational Level ---\n")
SDQ_nb_edu_int <- glm.nb(
  SDQ_Total ~ Group * Parent1_Education_level_num + ChildAge + ChildGender +
    ParentsSeparated + PHQ_Total,
  data = mydata)
print(summary(SDQ_nb_edu_int))

cat("\nLikelihood Ratio Test (Group × Index Parent Educational Level interaction):\n")
print(anova(SDQ_nb, SDQ_nb_edu_int, test = "Chisq"))
cat("\nInterpretation:\n")
cat("Non-significant Group × Index Parent Educational Level interaction indicates that group differences\n")
cat("in SDQ_Total are not moderated by parental educational level.\n")

## --- Age-stratified analyses ---
cat("\n--- Age-Stratified Models ---\n")
mydata <- mydata %>%
  mutate(AgeGroup = ifelse(ChildAge <= 5, "2–5 years", "6–12 years"))

SDQ_nb_age_young <- glm.nb(
  SDQ_Total ~ Group + ChildGender + ParentsSeparated +
    Parent1_Education_level_num + PHQ_Total,
  data = mydata,
  subset = AgeGroup == "2–5 years")

SDQ_nb_age_old <- glm.nb(
  SDQ_Total ~ Group + ChildGender + ParentsSeparated +
    Parent1_Education_level_num + PHQ_Total,
  data = mydata,
  subset = AgeGroup == "6–12 years")

cat("\nModel: 2–5 years\n")
print(model_parameters(SDQ_nb_age_young, exponentiate = TRUE))
cat("\nModel: 6–12 years\n")
print(model_parameters(SDQ_nb_age_old, exponentiate = TRUE))

cat("\nInterpretation:\n")
cat("Group effects show comparable direction and magnitude across age strata,\n")
cat("supporting robustness of findings across developmental stages.\n")

## --- Overlapping age range sensitivity (4–10 years) ---
cat("\n--- Sensitivity Analysis: Overlapping Age Range (4–10 years) ---\n")
SDQ_nb_overlap <- glm.nb(
  SDQ_Total ~ Group + ChildGender + ParentsSeparated +
    Parent1_Education_level_num + PHQ_Total,
  data = mydata,
  subset = ChildAge >= 4 & ChildAge <= 10)
print(model_parameters(SDQ_nb_overlap, exponentiate = TRUE))

cat("\nInterpretation:\n")
cat("Results within the overlapping age range are consistent with the main analysis.\n")
```

```
## 
## --- Interaction Test: Group × Child Age ---
## 
## Call:
## glm.nb(formula = SDQ_Total ~ Group * ChildAge + ChildGender + 
##     ParentsSeparated + Parent1_Education_level_num + PHQ_Total, 
##     data = mydata, init.theta = 3.60834796, link = log)
## 
## Coefficients:
##                               Estimate Std. Error z value Pr(>|z|)    
## (Intercept)                    2.26879    0.23719   9.565  < 2e-16 ***
## GroupSomatic Control          -0.35972    0.31132  -1.155  0.24789    
## GroupHealthy Control          -0.41168    0.27758  -1.483  0.13804    
## ChildAge                      -0.01972    0.02741  -0.719  0.47195    
## ChildGender                   -0.24541    0.08991  -2.729  0.00635 ** 
## ParentsSeparated               0.20352    0.10861   1.874  0.06096 .  
## Parent1_Education_level_num    0.11696    0.08104   1.443  0.14899    
## PHQ_Total                      0.10608    0.02073   5.118 3.08e-07 ***
## GroupSomatic Control:ChildAge -0.04937    0.04028  -1.226  0.22029    
## GroupHealthy Control:ChildAge -0.01492    0.03763  -0.397  0.69169    
## ---
## Signif. codes:  0 '***' 0.001 '**' 0.01 '*' 0.05 '.' 0.1 ' ' 1
## 
## (Dispersion parameter for Negative Binomial(3.6083) family taken to be 1)
## 
##     Null deviance: 396.79  on 214  degrees of freedom
## Residual deviance: 253.93  on 205  degrees of freedom
##   (3 observations deleted due to missingness)
## AIC: 1257.1
## 
## Number of Fisher Scoring iterations: 1
## 
## 
##               Theta:  3.608 
##           Std. Err.:  0.586 
## 
##  2 x log-likelihood:  -1235.068 
## 
## Likelihood Ratio Test (Group × Age interaction):
## Likelihood ratio tests of Negative Binomial Models
## 
## Response: SDQ_Total
##                                                                                         Model
## 1 Group + ChildGender + ChildAge + ParentsSeparated + Parent1_Education_level_num + PHQ_Total
## 2 Group * ChildAge + ChildGender + ParentsSeparated + Parent1_Education_level_num + PHQ_Total
##      theta Resid. df    2 x log-lik.   Test    df LR stat.   Pr(Chi)
## 1 3.561767       207       -1236.544                                
## 2 3.608348       205       -1235.068 1 vs 2     2  1.47682 0.4778731
## 
## Interpretation:
## Non-significant Group × Age interaction indicates that group differences in SDQ_Total
## are consistent across the age range studied.
## 
## --- Interaction Test: Group × Parental Separation ---
## 
## Call:
## glm.nb(formula = SDQ_Total ~ Group * ParentsSeparated + ChildAge + 
##     ChildGender + Parent1_Education_level_num + PHQ_Total, data = mydata, 
##     init.theta = 3.599897521, link = log)
## 
## Coefficients:
##                                       Estimate Std. Error z value Pr(>|z|)    
## (Intercept)                            2.35744    0.19021  12.394  < 2e-16 ***
## GroupSomatic Control                  -0.67417    0.14308  -4.712 2.45e-06 ***
## GroupHealthy Control                  -0.44362    0.12419  -3.572 0.000354 ***
## ParentsSeparated                       0.31577    0.15766   2.003 0.045189 *  
## ChildAge                              -0.03871    0.01586  -2.441 0.014642 *  
## ChildGender                           -0.24743    0.08992  -2.752 0.005931 ** 
## Parent1_Education_level_num            0.10856    0.08087   1.342 0.179474    
## PHQ_Total                              0.10869    0.02055   5.289 1.23e-07 ***
## GroupSomatic Control:ParentsSeparated -0.12447    0.27158  -0.458 0.646728    
## GroupHealthy Control:ParentsSeparated -0.25785    0.25432  -1.014 0.310648    
## ---
## Signif. codes:  0 '***' 0.001 '**' 0.01 '*' 0.05 '.' 0.1 ' ' 1
## 
## (Dispersion parameter for Negative Binomial(3.5999) family taken to be 1)
## 
##     Null deviance: 396.25  on 214  degrees of freedom
## Residual deviance: 254.07  on 205  degrees of freedom
##   (3 observations deleted due to missingness)
## AIC: 1257.5
## 
## Number of Fisher Scoring iterations: 1
## 
## 
##               Theta:  3.600 
##           Std. Err.:  0.584 
## 
##  2 x log-likelihood:  -1235.515 
## 
## Likelihood Ratio Test (Group × Parental Separation interaction):
## Likelihood ratio tests of Negative Binomial Models
## 
## Response: SDQ_Total
##                                                                                         Model
## 1 Group + ChildGender + ChildAge + ParentsSeparated + Parent1_Education_level_num + PHQ_Total
## 2 Group * ParentsSeparated + ChildAge + ChildGender + Parent1_Education_level_num + PHQ_Total
##      theta Resid. df    2 x log-lik.   Test    df LR stat.   Pr(Chi)
## 1 3.561767       207       -1236.544                                
## 2 3.599898       205       -1235.515 1 vs 2     2 1.029779 0.5975666
## 
## Interpretation:
## Non-significant Group × Parental Separation interaction indicates that group differences
## in SDQ_Total are not driven by family structure.
## 
## --- Interaction Test: Group × Child Gender ---
## 
## Call:
## glm.nb(formula = SDQ_Total ~ Group * ChildGender + ChildAge + 
##     ParentsSeparated + Parent1_Education_level_num + PHQ_Total, 
##     data = mydata, init.theta = 3.630413131, link = log)
## 
## Coefficients:
##                                  Estimate Std. Error z value Pr(>|z|)    
## (Intercept)                       2.43786    0.19420  12.553  < 2e-16 ***
## GroupSomatic Control             -0.87345    0.16707  -5.228 1.71e-07 ***
## GroupHealthy Control             -0.52531    0.14025  -3.746  0.00018 ***
## ChildGender                      -0.34821    0.15195  -2.292  0.02193 *  
## ChildAge                         -0.03947    0.01574  -2.507  0.01217 *  
## ParentsSeparated                  0.20744    0.10792   1.922  0.05459 .  
## Parent1_Education_level_num       0.11065    0.08070   1.371  0.17030    
## PHQ_Total                         0.11091    0.02049   5.412 6.23e-08 ***
## GroupSomatic Control:ChildGender  0.32158    0.23389   1.375  0.16916    
## GroupHealthy Control:ChildGender  0.05163    0.20775   0.249  0.80374    
## ---
## Signif. codes:  0 '***' 0.001 '**' 0.01 '*' 0.05 '.' 0.1 ' ' 1
## 
## (Dispersion parameter for Negative Binomial(3.6304) family taken to be 1)
## 
##     Null deviance: 398.19  on 214  degrees of freedom
## Residual deviance: 254.12  on 205  degrees of freedom
##   (3 observations deleted due to missingness)
## AIC: 1256.4
## 
## Number of Fisher Scoring iterations: 1
## 
## 
##               Theta:  3.630 
##           Std. Err.:  0.591 
## 
##  2 x log-likelihood:  -1234.436 
## 
## Likelihood Ratio Test (Group × Gender interaction):
## Likelihood ratio tests of Negative Binomial Models
## 
## Response: SDQ_Total
##                                                                                         Model
## 1 Group + ChildGender + ChildAge + ParentsSeparated + Parent1_Education_level_num + PHQ_Total
## 2 Group * ChildGender + ChildAge + ParentsSeparated + Parent1_Education_level_num + PHQ_Total
##      theta Resid. df    2 x log-lik.   Test    df LR stat.   Pr(Chi)
## 1 3.561767       207       -1236.544                                
## 2 3.630413       205       -1234.436 1 vs 2     2 2.108171 0.3485111
## 
## Interpretation:
## Non-significant Group × Gender interaction suggests that group effects are similar
## for boys and girls.
## 
## --- Interaction Test: Group × Index Parent Educational Level ---
## 
## Call:
## glm.nb(formula = SDQ_Total ~ Group * Parent1_Education_level_num + 
##     ChildAge + ChildGender + ParentsSeparated + PHQ_Total, data = mydata, 
##     init.theta = 3.585473957, link = log)
## 
## Coefficients:
##                                                  Estimate Std. Error z value
## (Intercept)                                       2.52106    0.23353  10.796
## GroupSomatic Control                             -0.90382    0.32517  -2.780
## GroupHealthy Control                             -0.71820    0.27349  -2.626
## Parent1_Education_level_num                       0.02685    0.12385   0.217
## ChildAge                                         -0.04062    0.01584  -2.564
## ChildGender                                      -0.24805    0.08994  -2.758
## ParentsSeparated                                  0.21678    0.10840   2.000
## PHQ_Total                                         0.11220    0.02061   5.443
## GroupSomatic Control:Parent1_Education_level_num  0.13108    0.20601   0.636
## GroupHealthy Control:Parent1_Education_level_num  0.15819    0.18521   0.854
##                                                  Pr(>|z|)    
## (Intercept)                                       < 2e-16 ***
## GroupSomatic Control                              0.00544 ** 
## GroupHealthy Control                              0.00864 ** 
## Parent1_Education_level_num                       0.82837    
## ChildAge                                          0.01034 *  
## ChildGender                                       0.00582 ** 
## ParentsSeparated                                  0.04552 *  
## PHQ_Total                                        5.23e-08 ***
## GroupSomatic Control:Parent1_Education_level_num  0.52461    
## GroupHealthy Control:Parent1_Education_level_num  0.39305    
## ---
## Signif. codes:  0 '***' 0.001 '**' 0.01 '*' 0.05 '.' 0.1 ' ' 1
## 
## (Dispersion parameter for Negative Binomial(3.5855) family taken to be 1)
## 
##     Null deviance: 395.32  on 214  degrees of freedom
## Residual deviance: 253.74  on 205  degrees of freedom
##   (3 observations deleted due to missingness)
## AIC: 1257.7
## 
## Number of Fisher Scoring iterations: 1
## 
## 
##               Theta:  3.585 
##           Std. Err.:  0.580 
## 
##  2 x log-likelihood:  -1235.720 
## 
## Likelihood Ratio Test (Group × Index Parent Educational Level interaction):
## Likelihood ratio tests of Negative Binomial Models
## 
## Response: SDQ_Total
##                                                                                         Model
## 1 Group + ChildGender + ChildAge + ParentsSeparated + Parent1_Education_level_num + PHQ_Total
## 2 Group * Parent1_Education_level_num + ChildAge + ChildGender + ParentsSeparated + PHQ_Total
##      theta Resid. df    2 x log-lik.   Test    df  LR stat.   Pr(Chi)
## 1 3.561767       207       -1236.544                                 
## 2 3.585474       205       -1235.720 1 vs 2     2 0.8240463 0.6623089
## 
## Interpretation:
## Non-significant Group × Index Parent Educational Level interaction indicates that group differences
## in SDQ_Total are not moderated by parental educational level.
## 
## --- Age-Stratified Models ---
## 
## Model: 2–5 years
## Parameter                   |   IRR |   SE |        95% CI |     z |      p
## ---------------------------------------------------------------------------
## (Intercept)                 | 12.32 | 2.59 | [8.23, 18.52] | 11.94 | < .001
## Group [Somatic Control]     |  0.59 | 0.10 | [0.43,  0.82] | -3.17 | 0.001 
## Group [Healthy Control]     |  0.53 | 0.08 | [0.40,  0.72] | -4.17 | < .001
## ChildGender                 |  0.63 | 0.08 | [0.49,  0.82] | -3.52 | < .001
## ParentsSeparated            |  1.06 | 0.17 | [0.78,  1.45] |  0.38 | 0.706 
## Parent1 Education level num |  1.00 | 0.11 | [0.82,  1.24] |  0.04 | 0.969 
## PHQ Total                   |  1.09 | 0.03 | [1.04,  1.15] |  3.38 | < .001
## 
## Model: 6–12 years
## Parameter                   |  IRR |   SE |        95% CI |     z |      p
## --------------------------------------------------------------------------
## (Intercept)                 | 6.71 | 1.48 | [4.37, 10.35] |  8.64 | < .001
## Group [Somatic Control]     | 0.42 | 0.07 | [0.30,  0.59] | -4.88 | < .001
## Group [Healthy Control]     | 0.63 | 0.10 | [0.46,  0.85] | -2.95 | 0.003 
## ChildGender                 | 0.85 | 0.10 | [0.67,  1.08] | -1.32 | 0.185 
## ParentsSeparated            | 1.24 | 0.18 | [0.93,  1.66] |  1.49 | 0.136 
## Parent1 Education level num | 1.21 | 0.14 | [0.96,  1.53] |  1.69 | 0.091 
## PHQ Total                   | 1.13 | 0.04 | [1.07,  1.21] |  4.06 | < .001
## 
## Interpretation:
## Group effects show comparable direction and magnitude across age strata,
## supporting robustness of findings across developmental stages.
## 
## --- Sensitivity Analysis: Overlapping Age Range (4–10 years) ---
## Parameter                   |  IRR |   SE |        95% CI |     z |      p
## --------------------------------------------------------------------------
## (Intercept)                 | 8.49 | 1.53 | [6.02, 12.02] | 11.85 | < .001
## Group [Somatic Control]     | 0.51 | 0.08 | [0.38,  0.69] | -4.45 | < .001
## Group [Healthy Control]     | 0.55 | 0.07 | [0.43,  0.71] | -4.64 | < .001
## ChildGender                 | 0.78 | 0.08 | [0.63,  0.96] | -2.31 | 0.021 
## ParentsSeparated            | 1.03 | 0.13 | [0.80,  1.34] |  0.26 | 0.797 
## Parent1 Education level num | 1.18 | 0.11 | [0.98,  1.42] |  1.74 | 0.083 
## PHQ Total                   | 1.13 | 0.03 | [1.07,  1.19] |  4.61 | < .001
## 
## Interpretation:
## Results within the overlapping age range are consistent with the main analysis.
```

### Evaluate nb-Model and calculate effect sizes

```
cat("\n--- Model Summary (Negative Binomial) ---\n")
summary(SDQ_nb)
cat("\n--- Regression Coefficients (log scale) ---\n")
print(coef(SDQ_nb))
cat("\n--- Exponentiated Coefficients (Rate Ratios) ---\n")
exp_coef <- exp(coef(SDQ_nb))
print(round(exp_coef, 3))
cat("\n--- 95% Confidence Intervals (log scale) ---\n")
confint_log <- suppressMessages(confint(SDQ_nb))
print(round(confint_log, 3))
cat("\n--- Exponentiated 95% Confidence Intervals (Rate Ratios) ---\n")
exp_confint <- exp(confint_log)
print(round(exp_confint, 3))

cat("\n--- Model Parameters (Exponentiated) ---\n") # Includes estimates, SE, CI, z, p, etc.
print(model_parameters(SDQ_nb, exponentiate = TRUE))
cat("\n--- Model Performance Metrics ---\n")
# Pseudo-R², AIC, RMSE, etc.
print(performance(SDQ_nb))

cat("\nInterpretation:\n")
cat("Exponentiated coefficients (Rate Ratios) > 1 indicate higher SDQ scores relative to reference.\n")
cat("Confidence intervals not crossing 1 indicate statistically significant effects.\n")
cat("Model diagnostics show good fit and robust parameter estimates.\n")
```

```
## 
## --- Model Summary (Negative Binomial) ---
## 
## Call:
## glm.nb(formula = SDQ_Total ~ Group + ChildGender + ChildAge + 
##     ParentsSeparated + Parent1_Education_level_num + PHQ_Total, 
##     data = mydata, init.theta = 3.561767479, link = log)
## 
## Coefficients:
##                             Estimate Std. Error z value Pr(>|z|)    
## (Intercept)                  2.39445    0.18758  12.765  < 2e-16 ***
## GroupSomatic Control        -0.71397    0.12538  -5.694 1.24e-08 ***
## GroupHealthy Control        -0.50131    0.11062  -4.532 5.84e-06 ***
## ChildGender                 -0.24553    0.09005  -2.726   0.0064 ** 
## ChildAge                    -0.03978    0.01580  -2.519   0.0118 *  
## ParentsSeparated             0.21254    0.10851   1.959   0.0501 .  
## Parent1_Education_level_num  0.11113    0.08115   1.369   0.1709    
## PHQ_Total                    0.11013    0.02056   5.357 8.47e-08 ***
## ---
## Signif. codes:  0 '***' 0.001 '**' 0.01 '*' 0.05 '.' 0.1 ' ' 1
## 
## (Dispersion parameter for Negative Binomial(3.5618) family taken to be 1)
## 
##     Null deviance: 393.80  on 214  degrees of freedom
## Residual deviance: 253.67  on 207  degrees of freedom
##   (3 observations deleted due to missingness)
## AIC: 1254.5
## 
## Number of Fisher Scoring iterations: 1
## 
## 
##               Theta:  3.562 
##           Std. Err.:  0.575 
## 
##  2 x log-likelihood:  -1236.544 
## 
## --- Regression Coefficients (log scale) ---
##                 (Intercept)        GroupSomatic Control 
##                  2.39445466                 -0.71397064 
##        GroupHealthy Control                 ChildGender 
##                 -0.50131471                 -0.24552584 
##                    ChildAge            ParentsSeparated 
##                 -0.03978497                  0.21254437 
## Parent1_Education_level_num                   PHQ_Total 
##                  0.11113079                  0.11013086 
## 
## --- Exponentiated Coefficients (Rate Ratios) ---
##                 (Intercept)        GroupSomatic Control 
##                      10.962                       0.490 
##        GroupHealthy Control                 ChildGender 
##                       0.606                       0.782 
##                    ChildAge            ParentsSeparated 
##                       0.961                       1.237 
## Parent1_Education_level_num                   PHQ_Total 
##                       1.118                       1.116 
## 
## --- 95% Confidence Intervals (log scale) ---
##                              2.5 % 97.5 %
## (Intercept)                  2.032  2.760
## GroupSomatic Control        -0.956 -0.472
## GroupHealthy Control        -0.718 -0.286
## ChildGender                 -0.423 -0.068
## ChildAge                    -0.071 -0.008
## ParentsSeparated            -0.001  0.428
## Parent1_Education_level_num -0.048  0.272
## PHQ_Total                    0.068  0.153
## 
## --- Exponentiated 95% Confidence Intervals (Rate Ratios) ---
##                             2.5 % 97.5 %
## (Intercept)                 7.629 15.805
## GroupSomatic Control        0.384  0.624
## GroupHealthy Control        0.488  0.752
## ChildGender                 0.655  0.934
## ChildAge                    0.931  0.992
## ParentsSeparated            0.999  1.535
## Parent1_Education_level_num 0.953  1.313
## PHQ_Total                   1.071  1.165
## 
## --- Model Parameters (Exponentiated) ---
## Parameter                   |   IRR |   SE |        95% CI |     z |      p
## ---------------------------------------------------------------------------
## (Intercept)                 | 10.96 | 2.06 | [7.63, 15.81] | 12.77 | < .001
## Group [Somatic Control]     |  0.49 | 0.06 | [0.38,  0.62] | -5.69 | < .001
## Group [Healthy Control]     |  0.61 | 0.07 | [0.49,  0.75] | -4.53 | < .001
## ChildGender                 |  0.78 | 0.07 | [0.66,  0.93] | -2.73 | 0.006 
## ChildAge                    |  0.96 | 0.02 | [0.93,  0.99] | -2.52 | 0.012 
## ParentsSeparated            |  1.24 | 0.13 | [1.00,  1.53] |  1.96 | 0.050 
## Parent1 Education level num |  1.12 | 0.09 | [0.95,  1.31] |  1.37 | 0.171 
## PHQ Total                   |  1.12 | 0.02 | [1.07,  1.17] |  5.36 | < .001
## 
## --- Model Performance Metrics ---
## # Indices of model performance
## 
## AIC      |     AICc |      BIC | Nagelkerke's R2 |  RMSE | Sigma | Score_log | Score_spherical
## ----------------------------------------------------------------------------------------------
## 1254.544 | 1255.422 | 1284.880 |           0.570 | 4.773 | 1.000 |    -2.879 |           0.059
## 
## Interpretation:
## Exponentiated coefficients (Rate Ratios) > 1 indicate higher SDQ scores relative to reference.
## Confidence intervals not crossing 1 indicate statistically significant effects.
## Model diagnostics show good fit and robust parameter estimates.
```

## Model for SDQ\_Emotional Problems 1.1 —

```
cat("\n--- Model for SDQ_Emotional Problems ---\n")
# Negative binomial model with confounders
SDQ_Emotional_nb <- glm.nb(
  SDQ_Emotional ~ Group + ChildGender + ChildAge + ParentsSeparated + 
    Parent1_Education_level_num + PHQ_Total,
  data = mydata)
summary(SDQ_Emotional_nb)

# Poisson model (comparison)
SDQ_Emotional_glm <- glm(
  SDQ_Emotional ~ Group + ChildGender + ChildAge + ParentsSeparated +
    Parent1_Education_level_num + PHQ_Total,
  family = poisson(link = "log"),
  data = mydata)
cat("\n--- Poisson Model (Comparison) ---\n")
summary(SDQ_Emotional_glm)
cat("\nInterpretation: Both models yield comparable results; PHQ_Total and Group remain significant predictors.\n")
```

```
## 
## --- Model for SDQ_Emotional Problems ---
## 
## Call:
## glm.nb(formula = SDQ_Emotional ~ Group + ChildGender + ChildAge + 
##     ParentsSeparated + Parent1_Education_level_num + PHQ_Total, 
##     data = mydata, init.theta = 4.077110258, link = log)
## 
## Coefficients:
##                             Estimate Std. Error z value Pr(>|z|)    
## (Intercept)                  0.86344    0.25335   3.408 0.000654 ***
## GroupSomatic Control        -0.87677    0.17129  -5.119 3.08e-07 ***
## GroupHealthy Control        -1.01599    0.15403  -6.596 4.22e-11 ***
## ChildGender                  0.06091    0.12468   0.489 0.625153    
## ChildAge                    -0.01288    0.02193  -0.587 0.556935    
## ParentsSeparated             0.04211    0.14757   0.285 0.775380    
## Parent1_Education_level_num  0.08072    0.10973   0.736 0.461934    
## PHQ_Total                    0.13454    0.02565   5.246 1.56e-07 ***
## ---
## Signif. codes:  0 '***' 0.001 '**' 0.01 '*' 0.05 '.' 0.1 ' ' 1
## 
## (Dispersion parameter for Negative Binomial(4.0771) family taken to be 1)
## 
##     Null deviance: 373.88  on 214  degrees of freedom
## Residual deviance: 248.75  on 207  degrees of freedom
##   (3 observations deleted due to missingness)
## AIC: 746.32
## 
## Number of Fisher Scoring iterations: 1
## 
## 
##               Theta:  4.08 
##           Std. Err.:  1.38 
## 
##  2 x log-likelihood:  -728.319 
## 
## --- Poisson Model (Comparison) ---
## 
## Call:
## glm(formula = SDQ_Emotional ~ Group + ChildGender + ChildAge + 
##     ParentsSeparated + Parent1_Education_level_num + PHQ_Total, 
##     family = poisson(link = "log"), data = mydata)
## 
## Coefficients:
##                              Estimate Std. Error z value Pr(>|z|)    
## (Intercept)                  0.852728   0.194480   4.385 1.16e-05 ***
## GroupSomatic Control        -0.885908   0.140215  -6.318 2.65e-10 ***
## GroupHealthy Control        -1.034866   0.126175  -8.202 2.37e-16 ***
## ChildGender                  0.083363   0.097496   0.855    0.393    
## ChildAge                    -0.001194   0.017083  -0.070    0.944    
## ParentsSeparated             0.057095   0.111991   0.510    0.610    
## Parent1_Education_level_num  0.060590   0.084294   0.719    0.472    
## PHQ_Total                    0.112764   0.018047   6.248 4.15e-10 ***
## ---
## Signif. codes:  0 '***' 0.001 '**' 0.01 '*' 0.05 '.' 0.1 ' ' 1
## 
## (Dispersion parameter for poisson family taken to be 1)
## 
##     Null deviance: 530.17  on 214  degrees of freedom
## Residual deviance: 340.96  on 207  degrees of freedom
##   (3 observations deleted due to missingness)
## AIC: 760.64
## 
## Number of Fisher Scoring iterations: 5
## 
## 
## Interpretation: Both models yield comparable results; PHQ_Total and Group remain significant predictors.
```

### Residuals, Outliers and Multicollinearity

```
cat("\n--- Multicollinearity Check (VIF) ---\n")
print(vif(SDQ_Emotional_nb))

# Residuals and outliers
predictions <- predict(SDQ_Emotional_nb, type = "response")
residuals_dev <- residuals(SDQ_Emotional_nb, type = "deviance")
resid_sd <- sd(residuals_dev)
upper <- mean(residuals_dev) + 2 * resid_sd
lower <- mean(residuals_dev) - 2 * resid_sd
outliers <- which(residuals_dev > upper | residuals_dev < lower)
cat("Number of detected outliers:", length(outliers), "\n")
if (length(outliers) > 0) {
  outlier_data <- data.frame(
    ID = mydata$ID_all[outliers],
    Residuals = residuals_dev[outliers],
    Predicted = predictions[outliers])
  print(outlier_data)}

# Visualization
plot_data <- data.frame(predicted = predictions, residuals = residuals_dev)
ggplot(plot_data, aes(x = predicted, y = residuals)) +
  geom_point(alpha = 0.7) +
  geom_hline(yintercept = 0, color = "red", linewidth = 0.5) +
  geom_point(data = plot_data[outliers, ], aes(x = predicted, y = residuals),
             color = "red", size = 2) +
  theme_minimal() +
  labs(x = "Predicted values", y = "Deviance residuals", 
       title = "Residuals vs Predicted (SDQ Emotional Model)")
```

```
# Refit model without outliers
mydata_Emotional_clean <- mydata[-outliers, ]
SDQ_Emotional_nb_clean <- glm.nb(
  SDQ_Emotional ~ Group + ChildGender + ChildAge + ParentsSeparated +
    Parent1_Education_level_num + PHQ_Total,
  data = mydata_Emotional_clean)
cat("\n--- Model without Outliers ---\n")
summary(SDQ_Emotional_nb_clean)

# AIC comparison
aic_old <- AIC(SDQ_Emotional_nb)
aic_new <- AIC(SDQ_Emotional_nb_clean)
cat("\nAIC (original):", round(aic_old, 2),
    "| AIC (without outliers):", round(aic_new, 2), "\n")
if (aic_new < aic_old) {
  cat("→ Slight improvement after removing outliers, interpretation unchanged.\n")
} else {
  cat("→ No relevant change after removing outliers.\n")}
```

```
## 
## --- Multicollinearity Check (VIF) ---
##                                 GVIF Df GVIF^(1/(2*Df))
## Group                       1.252909  2        1.057986
## ChildGender                 1.013610  1        1.006782
## ChildAge                    1.052917  1        1.026117
## ParentsSeparated            1.092395  1        1.045177
## Parent1_Education_level_num 1.072839  1        1.035779
## PHQ_Total                   1.125855  1        1.061063
## Number of detected outliers: 8 
##      ID Residuals Predicted
## 70   67  2.236230 1.4364862
## 74   71  2.209538 1.1118171
## 96   93  1.870204 1.8259050
## 100  97  1.841565 1.4448217
## 118 115  2.545010 1.4790940
## 315 143  1.929570 0.9729345
## 351 174  2.234558 1.4380778
## 400 213  2.114303 0.8395755
## 
## --- Model without Outliers ---
## 
## Call:
## glm.nb(formula = SDQ_Emotional ~ Group + ChildGender + ChildAge + 
##     ParentsSeparated + Parent1_Education_level_num + PHQ_Total, 
##     data = mydata_Emotional_clean, init.theta = 3.744234288, 
##     link = log)
## 
## Coefficients:
##                             Estimate Std. Error z value Pr(>|z|)    
## (Intercept)                  0.86872    0.25972   3.345 0.000823 ***
## GroupSomatic Control        -0.88461    0.17912  -4.939 7.86e-07 ***
## GroupHealthy Control        -0.99976    0.15868  -6.300 2.97e-10 ***
## ChildGender                  0.04716    0.12857   0.367 0.713741    
## ChildAge                    -0.01548    0.02274  -0.681 0.496110    
## ParentsSeparated             0.04065    0.15039   0.270 0.786953    
## Parent1_Education_level_num  0.09171    0.11228   0.817 0.414022    
## PHQ_Total                    0.13578    0.02664   5.097 3.45e-07 ***
## ---
## Signif. codes:  0 '***' 0.001 '**' 0.01 '*' 0.05 '.' 0.1 ' ' 1
## 
## (Dispersion parameter for Negative Binomial(3.7442) family taken to be 1)
## 
##     Null deviance: 358.45  on 206  degrees of freedom
## Residual deviance: 238.76  on 199  degrees of freedom
##   (3 observations deleted due to missingness)
## AIC: 723.88
## 
## Number of Fisher Scoring iterations: 1
## 
## 
##               Theta:  3.74 
##           Std. Err.:  1.23 
## 
##  2 x log-likelihood:  -705.883 
## 
## AIC (original): 746.32 | AIC (without outliers): 723.88 
## → Slight improvement after removing outliers, interpretation unchanged.
```

### Model with pooled imputed data

```
cat("\n--- Pooled Imputed Data Model ---\n")
SDQ_Emotional_nb_imp_pool <- with(
  imp_SDQ,
  glm.nb(SDQ_Emotional ~ Group + ChildGender + ChildAge + ParentsSeparated +
           Parent1_Education_level + PHQ_Total))
summary(pool(SDQ_Emotional_nb_imp_pool))

cat("\n--- Model on Single Imputed Dataset ---\n")
SDQ_Emotional_nb_imp <- glm.nb(
  SDQ_Emotional ~ Group + ChildGender + ChildAge + ParentsSeparated +
    Parent1_Education_level + PHQ_Total,
  data = mydata_SDQ_impute)
summary(SDQ_Emotional_nb_imp)
cat("\nInterpretation: No change in results; pooled and single-imputed models confirm robustness.\n")
```

```
## 
## --- Pooled Imputed Data Model ---
```

```
## 
## --- Model on Single Imputed Dataset ---
## 
## Call:
## glm.nb(formula = SDQ_Emotional ~ Group + ChildGender + ChildAge + 
##     ParentsSeparated + Parent1_Education_level + PHQ_Total, data = mydata_SDQ_impute, 
##     init.theta = 4.388193234, link = log)
## 
## Coefficients:
##                           Estimate Std. Error z value Pr(>|z|)    
## (Intercept)                0.98922    0.21145   4.678 2.89e-06 ***
## GroupSomatic Control      -0.88260    0.16789  -5.257 1.46e-07 ***
## GroupHealthy Control      -1.00968    0.15181  -6.651 2.91e-11 ***
## ChildGender                0.06040    0.12190   0.495    0.620    
## ChildAge                  -0.01180    0.02148  -0.549    0.583    
## ParentsSeparated           0.06129    0.14529   0.422    0.673    
## Parent1_Education_level.L  0.04413    0.20379   0.217    0.829    
## Parent1_Education_level.Q -0.08024    0.14412  -0.557    0.578    
## PHQ_Total                  0.13231    0.02517   5.256 1.47e-07 ***
## ---
## Signif. codes:  0 '***' 0.001 '**' 0.01 '*' 0.05 '.' 0.1 ' ' 1
## 
## (Dispersion parameter for Negative Binomial(4.3882) family taken to be 1)
## 
##     Null deviance: 383.95  on 217  degrees of freedom
## Residual deviance: 253.16  on 209  degrees of freedom
## AIC: 759.74
## 
## Number of Fisher Scoring iterations: 1
## 
## 
##               Theta:  4.39 
##           Std. Err.:  1.53 
## 
##  2 x log-likelihood:  -739.739 
## 
## Interpretation: No change in results; pooled and single-imputed models confirm robustness.
```

### Evaluate nb-Model and calculate effect sizes

```
cat("\n--- Model Evaluation and Effect Sizes (SDQ Emotional) ---\n")
summary(SDQ_Emotional_nb)

cat("\nLog-scale coefficients:\n")
print(round(coef(SDQ_Emotional_nb), 3))

cat("\nExponentiated coefficients (Rate Ratios):\n")
print(round(exp(coef(SDQ_Emotional_nb)), 3))
cat("\nExponentiated 95% Confidence Intervals:\n")
exp_confint <- exp(suppressMessages(confint(SDQ_Emotional_nb)))
print(round(exp_confint, 3))

cat("\nModel Parameters (Exponentiated):\n")
print(model_parameters(SDQ_Emotional_nb, exponentiate = TRUE))
cat("\nModel Performance:\n")
print(performance(SDQ_Emotional_nb))

cat("\nInterpretation:\n")
cat("Emotional problem scores are significantly higher in children with FGIDs,\n")
cat("driven primarily by Group and parental PHQ_Total. Model diagnostics show good fit.\n")
```

```
## 
## --- Model Evaluation and Effect Sizes (SDQ Emotional) ---
## 
## Call:
## glm.nb(formula = SDQ_Emotional ~ Group + ChildGender + ChildAge + 
##     ParentsSeparated + Parent1_Education_level_num + PHQ_Total, 
##     data = mydata, init.theta = 4.077110258, link = log)
## 
## Coefficients:
##                             Estimate Std. Error z value Pr(>|z|)    
## (Intercept)                  0.86344    0.25335   3.408 0.000654 ***
## GroupSomatic Control        -0.87677    0.17129  -5.119 3.08e-07 ***
## GroupHealthy Control        -1.01599    0.15403  -6.596 4.22e-11 ***
## ChildGender                  0.06091    0.12468   0.489 0.625153    
## ChildAge                    -0.01288    0.02193  -0.587 0.556935    
## ParentsSeparated             0.04211    0.14757   0.285 0.775380    
## Parent1_Education_level_num  0.08072    0.10973   0.736 0.461934    
## PHQ_Total                    0.13454    0.02565   5.246 1.56e-07 ***
## ---
## Signif. codes:  0 '***' 0.001 '**' 0.01 '*' 0.05 '.' 0.1 ' ' 1
## 
## (Dispersion parameter for Negative Binomial(4.0771) family taken to be 1)
## 
##     Null deviance: 373.88  on 214  degrees of freedom
## Residual deviance: 248.75  on 207  degrees of freedom
##   (3 observations deleted due to missingness)
## AIC: 746.32
## 
## Number of Fisher Scoring iterations: 1
## 
## 
##               Theta:  4.08 
##           Std. Err.:  1.38 
## 
##  2 x log-likelihood:  -728.319 
## 
## Log-scale coefficients:
##                 (Intercept)        GroupSomatic Control 
##                       0.863                      -0.877 
##        GroupHealthy Control                 ChildGender 
##                      -1.016                       0.061 
##                    ChildAge            ParentsSeparated 
##                      -0.013                       0.042 
## Parent1_Education_level_num                   PHQ_Total 
##                       0.081                       0.135 
## 
## Exponentiated coefficients (Rate Ratios):
##                 (Intercept)        GroupSomatic Control 
##                       2.371                       0.416 
##        GroupHealthy Control                 ChildGender 
##                       0.362                       1.063 
##                    ChildAge            ParentsSeparated 
##                       0.987                       1.043 
## Parent1_Education_level_num                   PHQ_Total 
##                       1.084                       1.144 
## 
## Exponentiated 95% Confidence Intervals:
##                             2.5 % 97.5 %
## (Intercept)                 1.428  3.925
## GroupSomatic Control        0.299  0.576
## GroupHealthy Control        0.267  0.488
## ChildGender                 0.832  1.357
## ChildAge                    0.945  1.031
## ParentsSeparated            0.779  1.391
## Parent1_Education_level_num 0.872  1.344
## PHQ_Total                   1.085  1.207
## 
## Model Parameters (Exponentiated):
## Parameter                   |  IRR |   SE |       95% CI |     z |      p
## -------------------------------------------------------------------------
## (Intercept)                 | 2.37 | 0.60 | [1.43, 3.92] |  3.41 | < .001
## Group [Somatic Control]     | 0.42 | 0.07 | [0.30, 0.58] | -5.12 | < .001
## Group [Healthy Control]     | 0.36 | 0.06 | [0.27, 0.49] | -6.60 | < .001
## ChildGender                 | 1.06 | 0.13 | [0.83, 1.36] |  0.49 | 0.625 
## ChildAge                    | 0.99 | 0.02 | [0.94, 1.03] | -0.59 | 0.557 
## ParentsSeparated            | 1.04 | 0.15 | [0.78, 1.39] |  0.29 | 0.775 
## Parent1 Education level num | 1.08 | 0.12 | [0.87, 1.34] |  0.74 | 0.462 
## PHQ Total                   | 1.14 | 0.03 | [1.09, 1.21] |  5.25 | < .001
## 
## Model Performance:
## # Indices of model performance
## 
## AIC     |    AICc |     BIC | Nagelkerke's R2 |  RMSE | Sigma | Score_log | Score_spherical
## -------------------------------------------------------------------------------------------
## 746.319 | 747.197 | 776.655 |           0.535 | 1.773 | 1.000 |    -1.695 |           0.059
## 
## Interpretation:
## Emotional problem scores are significantly higher in children with FGIDs,
## driven primarily by Group and parental PHQ_Total. Model diagnostics show good fit.
```

## Model for SDQ\_Conduct Problems 1.2 —

```
cat("\n--- Model for SDQ_Conduct Problems ---\n")
# Negative binomial model with confounders
SDQ_Conduct_nb <- glm.nb(
  SDQ_Conduct ~ Group + ChildGender + ChildAge + ParentsSeparated +
    Parent1_Education_level_num + PHQ_Total,
  data = mydata)
summary(SDQ_Conduct_nb)

cat("\nInterpretation: Children with FGIDs show significantly higher conduct problem scores;\n")
cat("both control groups display lower symptom levels. PHQ_Total, male gender, and younger age contribute to higher SDQ_Conduct scores.\n")

# Comparison Poisson model (for reference)
SDQ_Conduct_glm <- glm(
  SDQ_Conduct ~ Group + ChildGender + ChildAge + ParentsSeparated +
    Parent1_Education_level_num + PHQ_Total,
  family = poisson(link = "log"),
  data = mydata)
cat("\n--- Poisson Model (Comparison) ---\n")
summary(SDQ_Conduct_glm)
cat("\nMean vs Variance check: Overdispersion present, NB preferred.\n")
```

```
## 
## --- Model for SDQ_Conduct Problems ---
## 
## Call:
## glm.nb(formula = SDQ_Conduct ~ Group + ChildGender + ChildAge + 
##     ParentsSeparated + Parent1_Education_level_num + PHQ_Total, 
##     data = mydata, init.theta = 1.851704958, link = log)
## 
## Coefficients:
##                             Estimate Std. Error z value Pr(>|z|)    
## (Intercept)                  0.89655    0.30559   2.934 0.003348 ** 
## GroupSomatic Control        -0.78724    0.21397  -3.679 0.000234 ***
## GroupHealthy Control        -0.33099    0.17915  -1.848 0.064672 .  
## ChildGender                 -0.26063    0.14964  -1.742 0.081567 .  
## ChildAge                    -0.05873    0.02627  -2.236 0.025359 *  
## ParentsSeparated             0.37403    0.17477   2.140 0.032343 *  
## Parent1_Education_level_num  0.15652    0.13165   1.189 0.234481    
## PHQ_Total                    0.09969    0.03271   3.048 0.002304 ** 
## ---
## Signif. codes:  0 '***' 0.001 '**' 0.01 '*' 0.05 '.' 0.1 ' ' 1
## 
## (Dispersion parameter for Negative Binomial(1.8517) family taken to be 1)
## 
##     Null deviance: 298.35  on 214  degrees of freedom
## Residual deviance: 236.87  on 207  degrees of freedom
##   (3 observations deleted due to missingness)
## AIC: 759.61
## 
## Number of Fisher Scoring iterations: 1
## 
## 
##               Theta:  1.852 
##           Std. Err.:  0.434 
## 
##  2 x log-likelihood:  -741.607 
## 
## Interpretation: Children with FGIDs show significantly higher conduct problem scores;
## both control groups display lower symptom levels. PHQ_Total, male gender, and younger age contribute to higher SDQ_Conduct scores.
## 
## --- Poisson Model (Comparison) ---
## 
## Call:
## glm(formula = SDQ_Conduct ~ Group + ChildGender + ChildAge + 
##     ParentsSeparated + Parent1_Education_level_num + PHQ_Total, 
##     family = poisson(link = "log"), data = mydata)
## 
## Coefficients:
##                             Estimate Std. Error z value Pr(>|z|)    
## (Intercept)                  0.89292    0.20386   4.380 1.19e-05 ***
## GroupSomatic Control        -0.77978    0.15554  -5.014 5.34e-07 ***
## GroupHealthy Control        -0.37252    0.12155  -3.065 0.002179 ** 
## ChildGender                 -0.34981    0.10389  -3.367 0.000759 ***
## ChildAge                    -0.04633    0.01797  -2.578 0.009951 ** 
## ParentsSeparated             0.42692    0.11275   3.786 0.000153 ***
## Parent1_Education_level_num  0.14184    0.08596   1.650 0.098916 .  
## PHQ_Total                    0.08863    0.02039   4.347 1.38e-05 ***
## ---
## Signif. codes:  0 '***' 0.001 '**' 0.01 '*' 0.05 '.' 0.1 ' ' 1
## 
## (Dispersion parameter for poisson family taken to be 1)
## 
##     Null deviance: 536.70  on 214  degrees of freedom
## Residual deviance: 409.11  on 207  degrees of freedom
##   (3 observations deleted due to missingness)
## AIC: 805.35
## 
## Number of Fisher Scoring iterations: 6
## 
## 
## Mean vs Variance check: Overdispersion present, NB preferred.
```

### Residuals, Outliers and Multicollinearity

```
cat("\n--- Multicollinearity Check (VIF) ---\n")
print(vif(SDQ_Conduct_nb))

# Residuals and outliers
pred <- predict(SDQ_Conduct_nb, type = "response")
resid_dev <- residuals(SDQ_Conduct_nb, type = "deviance")
sd_resid <- sd(resid_dev)
upper <- mean(resid_dev) + 2 * sd_resid
lower <- mean(resid_dev) - 2 * sd_resid
outliers <- which(resid_dev > upper | resid_dev < lower)
cat("Number of detected outliers:", length(outliers), "\n")
if (length(outliers) > 0) {
  outlier_data <- data.frame(
    ID = mydata$ID_all[outliers],
    Residuals = resid_dev[outliers],
    Predicted = pred[outliers])
  print(outlier_data)}

# Visualization
ggplot(data.frame(predicted = pred, residuals = resid_dev), aes(x = predicted, y = residuals)) +
  geom_point(alpha = 0.7) +
  geom_hline(yintercept = 0, color = "red", linewidth = 0.5) +
  geom_point(data = subset(data.frame(predicted = pred, residuals = resid_dev), residuals %in% resid_dev[outliers]),
             color = "red", size = 2) +
  theme_minimal() +
  labs(x = "Predicted values", y = "Deviance residuals", 
       title = "Residuals vs Predicted (SDQ Conduct Model)")
```

```
# Refit model without outliers
mydata_Conduct_clean <- mydata[-outliers, ]
SDQ_Conduct_nb_clean <- glm.nb(
  SDQ_Conduct ~ Group + ChildGender + ChildAge + ParentsSeparated +
    Parent1_Education_level_num + PHQ_Total,
  data = mydata_Conduct_clean)
cat("\n--- Model without Outliers ---\n")
summary(SDQ_Conduct_nb_clean)

# AIC comparison
aic_old <- AIC(SDQ_Conduct_nb)
aic_new <- AIC(SDQ_Conduct_nb_clean)
cat("\nAIC (original):", round(aic_old, 2), "| AIC (without outliers):", round(aic_new, 2), "\n")
if (aic_new < aic_old) {
  cat("→ Slight improvement after removing outliers, interpretation unchanged.\n")
} else {
  cat("→ No relevant change after removing outliers.\n")}
```

```
## 
## --- Multicollinearity Check (VIF) ---
##                                 GVIF Df GVIF^(1/(2*Df))
## Group                       1.238596  2        1.054951
## ChildGender                 1.014107  1        1.007029
## ChildAge                    1.046834  1        1.023149
## ParentsSeparated            1.091169  1        1.044590
## Parent1_Education_level_num 1.077841  1        1.038191
## PHQ_Total                   1.116955  1        1.056861
## Number of detected outliers: 10 
##      ID Residuals Predicted
## 17   17  1.818018 2.1370152
## 57   54  2.152169 2.2662855
## 72   69  1.754787 0.5925109
## 74   71  2.413725 0.7348188
## 76   73  1.777912 1.5328649
## 103 100  1.771894 1.2126760
## 118 115  2.162472 1.1592982
## 335 159  1.941254 1.3612970
## 373 193  2.256835 0.8314370
## 398 211  2.054092 0.9741607
## 
## --- Model without Outliers ---
## 
## Call:
## glm.nb(formula = SDQ_Conduct ~ Group + ChildGender + ChildAge + 
##     ParentsSeparated + Parent1_Education_level_num + PHQ_Total, 
##     data = mydata_Conduct_clean, init.theta = 1.882844301, link = log)
## 
## Coefficients:
##                             Estimate Std. Error z value Pr(>|z|)   
## (Intercept)                  0.78512    0.31541   2.489  0.01280 * 
## GroupSomatic Control        -0.71518    0.22108  -3.235  0.00122 **
## GroupHealthy Control        -0.26380    0.18460  -1.429  0.15299   
## ChildGender                 -0.24830    0.15227  -1.631  0.10296   
## ChildAge                    -0.06186    0.02679  -2.309  0.02096 * 
## ParentsSeparated             0.43065    0.17712   2.431  0.01504 * 
## Parent1_Education_level_num  0.19204    0.13575   1.415  0.15717   
## PHQ_Total                    0.10485    0.03292   3.185  0.00145 **
## ---
## Signif. codes:  0 '***' 0.001 '**' 0.01 '*' 0.05 '.' 0.1 ' ' 1
## 
## (Dispersion parameter for Negative Binomial(1.8828) family taken to be 1)
## 
##     Null deviance: 287.54  on 204  degrees of freedom
## Residual deviance: 227.05  on 197  degrees of freedom
##   (3 observations deleted due to missingness)
## AIC: 725.2
## 
## Number of Fisher Scoring iterations: 1
## 
## 
##               Theta:  1.883 
##           Std. Err.:  0.459 
## 
##  2 x log-likelihood:  -707.203 
## 
## AIC (original): 759.61 | AIC (without outliers): 725.2 
## → Slight improvement after removing outliers, interpretation unchanged.
```

### Model with pooled imputed data

```
cat("\n--- Pooled Imputed Data Model ---\n")
SDQ_Conduct_nb_imp_pool <- with(
  imp_SDQ,
  glm.nb(SDQ_Conduct ~ Group + ChildGender + ChildAge + ParentsSeparated +
           Parent1_Education_level + PHQ_Total))
summary(pool(SDQ_Conduct_nb_imp_pool))

cat("\n--- Single Imputation Model ---\n")
SDQ_Conduct_nb_imp <- glm.nb(
  SDQ_Conduct ~ Group + ChildGender + ChildAge + ParentsSeparated +
    Parent1_Education_level + PHQ_Total,
  data = mydata_SDQ_impute)
summary(SDQ_Conduct_nb_imp)
cat("\nInterpretation: Results remain stable; imputation slightly strengthens significance for the healthy group.\n")
```

```
## 
## --- Pooled Imputed Data Model ---
```

```
## 
## --- Single Imputation Model ---
## 
## Call:
## glm.nb(formula = SDQ_Conduct ~ Group + ChildGender + ChildAge + 
##     ParentsSeparated + Parent1_Education_level + PHQ_Total, data = mydata_SDQ_impute, 
##     init.theta = 1.893817297, link = log)
## 
## Coefficients:
##                           Estimate Std. Error z value Pr(>|z|)    
## (Intercept)                1.21396    0.25528   4.755 1.98e-06 ***
## GroupSomatic Control      -0.79473    0.21083  -3.770 0.000164 ***
## GroupHealthy Control      -0.33905    0.17689  -1.917 0.055278 .  
## ChildGender               -0.25153    0.14734  -1.707 0.087797 .  
## ChildAge                  -0.05296    0.02590  -2.045 0.040875 *  
## ParentsSeparated           0.39572    0.17286   2.289 0.022065 *  
## Parent1_Education_level.L  0.32241    0.23291   1.384 0.166270    
## Parent1_Education_level.Q  0.06420    0.17057   0.376 0.706642    
## PHQ_Total                  0.09765    0.03241   3.013 0.002590 ** 
## ---
## Signif. codes:  0 '***' 0.001 '**' 0.01 '*' 0.05 '.' 0.1 ' ' 1
## 
## (Dispersion parameter for Negative Binomial(1.8938) family taken to be 1)
## 
##     Null deviance: 304.41  on 217  degrees of freedom
## Residual deviance: 240.70  on 209  degrees of freedom
## AIC: 774.81
## 
## Number of Fisher Scoring iterations: 1
## 
## 
##               Theta:  1.894 
##           Std. Err.:  0.443 
## 
##  2 x log-likelihood:  -754.811 
## 
## Interpretation: Results remain stable; imputation slightly strengthens significance for the healthy group.
```

### Evaluate nb-Model and calculate effect sizes

```
cat("\n--- Model Evaluation and Effect Sizes (SDQ Conduct) ---\n")
summary(SDQ_Conduct_nb)

cat("\nLog-scale coefficients:\n")
print(round(coef(SDQ_Conduct_nb), 3))
cat("\nExponentiated coefficients (Rate Ratios):\n")
print(round(exp(coef(SDQ_Conduct_nb)), 3))
cat("\nExponentiated 95% Confidence Intervals:\n")
exp_confint <- exp(suppressMessages(confint(SDQ_Conduct_nb)))
print(round(exp_confint, 3))

cat("\nModel Parameters (Exponentiated):\n")
print(model_parameters(SDQ_Conduct_nb, exponentiate = TRUE))

cat("\nModel Performance:\n")
print(performance(SDQ_Conduct_nb))

cat("\nInterpretation:\n")
cat("Conduct problems were significantly more pronounced in FGID children,\n")
cat("with PHQ_Total, male gender, and younger age emerging as key covariates.\n")
cat("Model fit was adequate (Nagelkerke R² ≈ 0.33, AIC ~760).\n")
```

```
## 
## --- Model Evaluation and Effect Sizes (SDQ Conduct) ---
## 
## Call:
## glm.nb(formula = SDQ_Conduct ~ Group + ChildGender + ChildAge + 
##     ParentsSeparated + Parent1_Education_level_num + PHQ_Total, 
##     data = mydata, init.theta = 1.851704958, link = log)
## 
## Coefficients:
##                             Estimate Std. Error z value Pr(>|z|)    
## (Intercept)                  0.89655    0.30559   2.934 0.003348 ** 
## GroupSomatic Control        -0.78724    0.21397  -3.679 0.000234 ***
## GroupHealthy Control        -0.33099    0.17915  -1.848 0.064672 .  
## ChildGender                 -0.26063    0.14964  -1.742 0.081567 .  
## ChildAge                    -0.05873    0.02627  -2.236 0.025359 *  
## ParentsSeparated             0.37403    0.17477   2.140 0.032343 *  
## Parent1_Education_level_num  0.15652    0.13165   1.189 0.234481    
## PHQ_Total                    0.09969    0.03271   3.048 0.002304 ** 
## ---
## Signif. codes:  0 '***' 0.001 '**' 0.01 '*' 0.05 '.' 0.1 ' ' 1
## 
## (Dispersion parameter for Negative Binomial(1.8517) family taken to be 1)
## 
##     Null deviance: 298.35  on 214  degrees of freedom
## Residual deviance: 236.87  on 207  degrees of freedom
##   (3 observations deleted due to missingness)
## AIC: 759.61
## 
## Number of Fisher Scoring iterations: 1
## 
## 
##               Theta:  1.852 
##           Std. Err.:  0.434 
## 
##  2 x log-likelihood:  -741.607 
## 
## Log-scale coefficients:
##                 (Intercept)        GroupSomatic Control 
##                       0.897                      -0.787 
##        GroupHealthy Control                 ChildGender 
##                      -0.331                      -0.261 
##                    ChildAge            ParentsSeparated 
##                      -0.059                       0.374 
## Parent1_Education_level_num                   PHQ_Total 
##                       0.157                       0.100 
## 
## Exponentiated coefficients (Rate Ratios):
##                 (Intercept)        GroupSomatic Control 
##                       2.451                       0.455 
##        GroupHealthy Control                 ChildGender 
##                       0.718                       0.771 
##                    ChildAge            ParentsSeparated 
##                       0.943                       1.454 
## Parent1_Education_level_num                   PHQ_Total 
##                       1.169                       1.105 
## 
## Exponentiated 95% Confidence Intervals:
##                             2.5 % 97.5 %
## (Intercept)                 1.372  4.391
## GroupSomatic Control        0.301  0.685
## GroupHealthy Control        0.504  1.023
## ChildGender                 0.573  1.035
## ChildAge                    0.895  0.994
## ParentsSeparated            1.028  2.058
## Parent1_Education_level_num 0.905  1.514
## PHQ_Total                   1.035  1.181
## 
## Model Parameters (Exponentiated):
## Parameter                   |  IRR |   SE |       95% CI |     z |      p
## -------------------------------------------------------------------------
## (Intercept)                 | 2.45 | 0.75 | [1.37, 4.39] |  2.93 | 0.003 
## Group [Somatic Control]     | 0.46 | 0.10 | [0.30, 0.68] | -3.68 | < .001
## Group [Healthy Control]     | 0.72 | 0.13 | [0.50, 1.02] | -1.85 | 0.065 
## ChildGender                 | 0.77 | 0.12 | [0.57, 1.04] | -1.74 | 0.082 
## ChildAge                    | 0.94 | 0.02 | [0.89, 0.99] | -2.24 | 0.025 
## ParentsSeparated            | 1.45 | 0.25 | [1.03, 2.06] |  2.14 | 0.032 
## Parent1 Education level num | 1.17 | 0.15 | [0.91, 1.51] |  1.19 | 0.234 
## PHQ Total                   | 1.10 | 0.04 | [1.04, 1.18] |  3.05 | 0.002 
## 
## Model Performance:
## # Indices of model performance
## 
## AIC     |    AICc |     BIC | Nagelkerke's R2 |  RMSE | Sigma | Score_log | Score_spherical
## -------------------------------------------------------------------------------------------
## 759.607 | 760.485 | 789.943 |           0.331 | 1.865 | 1.000 |    -1.733 |           0.058
## 
## Interpretation:
## Conduct problems were significantly more pronounced in FGID children,
## with PHQ_Total, male gender, and younger age emerging as key covariates.
## Model fit was adequate (Nagelkerke R² ≈ 0.33, AIC ~760).
```

## Model for SDQ\_Hyperactivity/Inattention 1.3 —

```
cat("\n--- Model for SDQ_Hyperactivity/Inattention ---\n")

# Negative binomial model with confounders
SDQ_Hyperact_nb <- glm.nb(
  SDQ_Hyperact ~ Group + ChildGender + ChildAge + ParentsSeparated +
    Parent1_Education_level_num + PHQ_Total,
  data = mydata)
summary(SDQ_Hyperact_nb)
cat("\nInterpretation: Children with FGIDs show significantly higher hyperactivity/inattention scores.\n")
cat("Both control groups show lower scores. PHQ_Total, male gender, and younger age contribute to higher SDQ_Hyperact scores.\n")

# Comparison model (Poisson)
SDQ_Hyperact_glm <- glm(
  SDQ_Hyperact ~ Group + ChildGender + ChildAge + ParentsSeparated +
    Parent1_Education_level_num + PHQ_Total,
  family = poisson(link = "log"),
  data = mydata)
cat("\n--- Poisson Model (Comparison) ---\n")
summary(SDQ_Hyperact_glm)
cat("\nOverdispersion detected (Var > Mean), NB model preferred.\n")
```

```
## 
## --- Model for SDQ_Hyperactivity/Inattention ---
## 
## Call:
## glm.nb(formula = SDQ_Hyperact ~ Group + ChildGender + ChildAge + 
##     ParentsSeparated + Parent1_Education_level_num + PHQ_Total, 
##     data = mydata, init.theta = 4.542641712, link = log)
## 
## Coefficients:
##                             Estimate Std. Error z value Pr(>|z|)    
## (Intercept)                  1.61337    0.21271   7.585 3.33e-14 ***
## GroupSomatic Control        -0.41445    0.14358  -2.887  0.00389 ** 
## GroupHealthy Control        -0.30425    0.12635  -2.408  0.01604 *  
## ChildGender                 -0.43228    0.10454  -4.135 3.55e-05 ***
## ChildAge                    -0.04824    0.01806  -2.671  0.00757 ** 
## ParentsSeparated             0.29137    0.12142   2.400  0.01641 *  
## Parent1_Education_level_num  0.07290    0.09259   0.787  0.43104    
## PHQ_Total                    0.04938    0.02349   2.102  0.03555 *  
## ---
## Signif. codes:  0 '***' 0.001 '**' 0.01 '*' 0.05 '.' 0.1 ' ' 1
## 
## (Dispersion parameter for Negative Binomial(4.5426) family taken to be 1)
## 
##     Null deviance: 327.13  on 214  degrees of freedom
## Residual deviance: 262.31  on 207  degrees of freedom
##   (3 observations deleted due to missingness)
## AIC: 924.57
## 
## Number of Fisher Scoring iterations: 1
## 
## 
##               Theta:  4.54 
##           Std. Err.:  1.28 
## 
##  2 x log-likelihood:  -906.571 
## 
## Interpretation: Children with FGIDs show significantly higher hyperactivity/inattention scores.
## Both control groups show lower scores. PHQ_Total, male gender, and younger age contribute to higher SDQ_Hyperact scores.
## 
## --- Poisson Model (Comparison) ---
## 
## Call:
## glm(formula = SDQ_Hyperact ~ Group + ChildGender + ChildAge + 
##     ParentsSeparated + Parent1_Education_level_num + PHQ_Total, 
##     family = poisson(link = "log"), data = mydata)
## 
## Coefficients:
##                             Estimate Std. Error z value Pr(>|z|)    
## (Intercept)                  1.63576    0.15696  10.421  < 2e-16 ***
## GroupSomatic Control        -0.44918    0.10980  -4.091 4.30e-05 ***
## GroupHealthy Control        -0.32774    0.09405  -3.485 0.000492 ***
## ChildGender                 -0.43713    0.08048  -5.431 5.59e-08 ***
## ChildAge                    -0.04696    0.01366  -3.437 0.000588 ***
## ParentsSeparated             0.27503    0.08888   3.094 0.001973 ** 
## Parent1_Education_level_num  0.07661    0.06802   1.126 0.260086    
## PHQ_Total                    0.04312    0.01710   2.522 0.011668 *  
## ---
## Signif. codes:  0 '***' 0.001 '**' 0.01 '*' 0.05 '.' 0.1 ' ' 1
## 
## (Dispersion parameter for poisson family taken to be 1)
## 
##     Null deviance: 504.30  on 214  degrees of freedom
## Residual deviance: 392.26  on 207  degrees of freedom
##   (3 observations deleted due to missingness)
## AIC: 948.38
## 
## Number of Fisher Scoring iterations: 5
## 
## 
## Overdispersion detected (Var > Mean), NB model preferred.
```

### Residuals, Outliers, and Multicollinearity

```
cat("\n--- Multicollinearity Check (VIF) ---\n")
print(vif(SDQ_Hyperact_nb))

# Residuals and outliers
predictions <- predict(SDQ_Hyperact_nb, type = "response")
residuals_dev <- residuals(SDQ_Hyperact_nb, type = "deviance")
resid_sd <- sd(residuals_dev)
upper <- mean(residuals_dev) + 2 * resid_sd
lower <- mean(residuals_dev) - 2 * resid_sd
outliers <- which(residuals_dev > upper | residuals_dev < lower)
cat("Number of detected outliers:", length(outliers), "\n")
if (length(outliers) > 0) {
  outlier_data <- data.frame(
    ID = mydata$ID_all[outliers],
    Residuals = residuals_dev[outliers],
    Predicted = predictions[outliers])
  print(outlier_data)}

# Visualization
ggplot(data.frame(predicted = predictions, residuals = residuals_dev),
       aes(x = predicted, y = residuals)) +
  geom_point(alpha = 0.7) +
  geom_hline(yintercept = 0, color = "red", linewidth = 0.5) +
  geom_point(data = subset(data.frame(predicted = predictions, residuals = residuals_dev),
                           residuals %in% residuals_dev[outliers]),
             color = "red", size = 2) +
  theme_minimal() +
  labs(x = "Predicted values", y = "Deviance residuals", 
       title = "Residuals vs Predicted (SDQ Hyperactivity Model)")
```

```
# Refit model without outliers
mydata_Hyperact_clean <- mydata[-outliers, ]
SDQ_Hyperact_nb_clean <- glm.nb(
  SDQ_Hyperact ~ Group + ChildGender + ChildAge + ParentsSeparated +
    Parent1_Education_level_num + PHQ_Total,
  data = mydata_Hyperact_clean)
cat("\n--- Model without Outliers ---\n")
summary(SDQ_Hyperact_nb_clean)

# AIC comparison
aic_old <- AIC(SDQ_Hyperact_nb)
aic_new <- AIC(SDQ_Hyperact_nb_clean)
cat("\nAIC (original):", round(aic_old, 2), "| AIC (without outliers):", round(aic_new, 2), "\n")
if (aic_new < aic_old) {
  cat("→ Slight improvement after removing outliers, interpretation unchanged.\n")
} else {
  cat("→ No relevant change after removing outliers.\n")}
```

```
## 
## --- Multicollinearity Check (VIF) ---
##                                 GVIF Df GVIF^(1/(2*Df))
## Group                       1.241023  2        1.055468
## ChildGender                 1.013576  1        1.006765
## ChildAge                    1.050729  1        1.025051
## ParentsSeparated            1.085593  1        1.041918
## Parent1_Education_level_num 1.079038  1        1.038767
## PHQ_Total                   1.122366  1        1.059418
## Number of detected outliers: 5 
##      ID Residuals Predicted
## 45   42 -2.412185  4.076236
## 321 148  2.147124  3.138899
## 335 159  1.996544  2.136237
## 391 206 -2.481960  4.406360
## 402 214 -2.395325  3.999685
## 
## --- Model without Outliers ---
## 
## Call:
## glm.nb(formula = SDQ_Hyperact ~ Group + ChildGender + ChildAge + 
##     ParentsSeparated + Parent1_Education_level_num + PHQ_Total, 
##     data = mydata_Hyperact_clean, init.theta = 4.702408953, link = log)
## 
## Coefficients:
##                             Estimate Std. Error z value Pr(>|z|)    
## (Intercept)                  1.59498    0.21237   7.510 5.90e-14 ***
## GroupSomatic Control        -0.40745    0.14306  -2.848  0.00440 ** 
## GroupHealthy Control        -0.27017    0.12723  -2.123  0.03372 *  
## ChildGender                 -0.44621    0.10493  -4.253 2.11e-05 ***
## ChildAge                    -0.04818    0.01803  -2.673  0.00752 ** 
## ParentsSeparated             0.32886    0.12263   2.682  0.00733 ** 
## Parent1_Education_level_num  0.08293    0.09429   0.880  0.37912    
## PHQ_Total                    0.04543    0.02348   1.935  0.05297 .  
## ---
## Signif. codes:  0 '***' 0.001 '**' 0.01 '*' 0.05 '.' 0.1 ' ' 1
## 
## (Dispersion parameter for Negative Binomial(4.7024) family taken to be 1)
## 
##     Null deviance: 320.56  on 209  degrees of freedom
## Residual deviance: 255.19  on 202  degrees of freedom
##   (3 observations deleted due to missingness)
## AIC: 903.57
## 
## Number of Fisher Scoring iterations: 1
## 
## 
##               Theta:  4.70 
##           Std. Err.:  1.35 
## 
##  2 x log-likelihood:  -885.571 
## 
## AIC (original): 924.57 | AIC (without outliers): 903.57 
## → Slight improvement after removing outliers, interpretation unchanged.
```

### Model with pooled imputed data

```
cat("\n--- Pooled Imputed Data Model ---\n")
SDQ_Hyperact_nb_imp_pool <- with(
  imp_SDQ,
  glm.nb(SDQ_Hyperact ~ Group + ChildGender + ChildAge + ParentsSeparated +
           Parent1_Education_level + PHQ_Total))
summary(pool(SDQ_Hyperact_nb_imp_pool))

cat("\n--- Model on Single Imputed Dataset ---\n")
SDQ_Hyperact_nb_imp <- glm.nb(
  SDQ_Hyperact ~ Group + ChildGender + ChildAge + ParentsSeparated +
    Parent1_Education_level + PHQ_Total,
  data = mydata_SDQ_impute)
summary(SDQ_Hyperact_nb_imp)
cat("\nInterpretation: Results remain broadly consistent across original, cleaned, and imputed data.\n")
cat("Model with outlier removal (SDQ_Hyperact_nb_clean) shows best AIC fit.\n")
```

```
## 
## --- Pooled Imputed Data Model ---
```

```
## 
## --- Model on Single Imputed Dataset ---
## 
## Call:
## glm.nb(formula = SDQ_Hyperact ~ Group + ChildGender + ChildAge + 
##     ParentsSeparated + Parent1_Education_level + PHQ_Total, data = mydata_SDQ_impute, 
##     init.theta = 4.499065519, link = log)
## 
## Coefficients:
##                           Estimate Std. Error z value Pr(>|z|)    
## (Intercept)                1.74795    0.17996   9.713  < 2e-16 ***
## GroupSomatic Control      -0.42262    0.14257  -2.964  0.00303 ** 
## GroupHealthy Control      -0.30451    0.12576  -2.421  0.01547 *  
## ChildGender               -0.42747    0.10375  -4.120 3.79e-05 ***
## ChildAge                  -0.04390    0.01797  -2.443  0.01456 *  
## ParentsSeparated           0.31313    0.12123   2.583  0.00980 ** 
## Parent1_Education_level.L  0.14929    0.17108   0.873  0.38287    
## Parent1_Education_level.Q  0.01333    0.12324   0.108  0.91389    
## PHQ_Total                  0.04804    0.02351   2.044  0.04100 *  
## ---
## Signif. codes:  0 '***' 0.001 '**' 0.01 '*' 0.05 '.' 0.1 ' ' 1
## 
## (Dispersion parameter for Negative Binomial(4.4991) family taken to be 1)
## 
##     Null deviance: 330.10  on 217  degrees of freedom
## Residual deviance: 265.05  on 209  degrees of freedom
## AIC: 940.78
## 
## Number of Fisher Scoring iterations: 1
## 
## 
##               Theta:  4.50 
##           Std. Err.:  1.24 
## 
##  2 x log-likelihood:  -920.78 
## 
## Interpretation: Results remain broadly consistent across original, cleaned, and imputed data.
## Model with outlier removal (SDQ_Hyperact_nb_clean) shows best AIC fit.
```

### Evaluate nb-Model and calculate effect sizes

```
cat("\n--- Model Evaluation and Effect Sizes (SDQ Hyperactivity) ---\n")
summary(SDQ_Hyperact_nb)

cat("\nLog-scale coefficients:\n")
print(round(coef(SDQ_Hyperact_nb), 3))
cat("\nExponentiated coefficients (Rate Ratios):\n")
print(round(exp(coef(SDQ_Hyperact_nb)), 3))
cat("\nExponentiated 95% Confidence Intervals:\n")
exp_confint <- exp(suppressMessages(confint(SDQ_Hyperact_nb)))
print(round(exp_confint, 3))

cat("\nModel Parameters (Exponentiated):\n")
print(model_parameters(SDQ_Hyperact_nb, exponentiate = TRUE))
cat("\nModel Performance:\n")
print(performance(SDQ_Hyperact_nb))

cat("\nInterpretation:\n")
cat("Children with FGIDs show higher hyperactivity/inattention symptoms compared to both control groups.\n")
cat("Overall model fit remains good (Nagelkerke R² ≈ 0.33; AIC ≈ 924).\n")
```

```
## 
## --- Model Evaluation and Effect Sizes (SDQ Hyperactivity) ---
## 
## Call:
## glm.nb(formula = SDQ_Hyperact ~ Group + ChildGender + ChildAge + 
##     ParentsSeparated + Parent1_Education_level_num + PHQ_Total, 
##     data = mydata, init.theta = 4.542641712, link = log)
## 
## Coefficients:
##                             Estimate Std. Error z value Pr(>|z|)    
## (Intercept)                  1.61337    0.21271   7.585 3.33e-14 ***
## GroupSomatic Control        -0.41445    0.14358  -2.887  0.00389 ** 
## GroupHealthy Control        -0.30425    0.12635  -2.408  0.01604 *  
## ChildGender                 -0.43228    0.10454  -4.135 3.55e-05 ***
## ChildAge                    -0.04824    0.01806  -2.671  0.00757 ** 
## ParentsSeparated             0.29137    0.12142   2.400  0.01641 *  
## Parent1_Education_level_num  0.07290    0.09259   0.787  0.43104    
## PHQ_Total                    0.04938    0.02349   2.102  0.03555 *  
## ---
## Signif. codes:  0 '***' 0.001 '**' 0.01 '*' 0.05 '.' 0.1 ' ' 1
## 
## (Dispersion parameter for Negative Binomial(4.5426) family taken to be 1)
## 
##     Null deviance: 327.13  on 214  degrees of freedom
## Residual deviance: 262.31  on 207  degrees of freedom
##   (3 observations deleted due to missingness)
## AIC: 924.57
## 
## Number of Fisher Scoring iterations: 1
## 
## 
##               Theta:  4.54 
##           Std. Err.:  1.28 
## 
##  2 x log-likelihood:  -906.571 
## 
## Log-scale coefficients:
##                 (Intercept)        GroupSomatic Control 
##                       1.613                      -0.414 
##        GroupHealthy Control                 ChildGender 
##                      -0.304                      -0.432 
##                    ChildAge            ParentsSeparated 
##                      -0.048                       0.291 
## Parent1_Education_level_num                   PHQ_Total 
##                       0.073                       0.049 
## 
## Exponentiated coefficients (Rate Ratios):
##                 (Intercept)        GroupSomatic Control 
##                       5.020                       0.661 
##        GroupHealthy Control                 ChildGender 
##                       0.738                       0.649 
##                    ChildAge            ParentsSeparated 
##                       0.953                       1.338 
## Parent1_Education_level_num                   PHQ_Total 
##                       1.076                       1.051 
## 
## Exponentiated 95% Confidence Intervals:
##                             2.5 % 97.5 %
## (Intercept)                 3.296  7.651
## GroupSomatic Control        0.498  0.876
## GroupHealthy Control        0.576  0.944
## ChildGender                 0.528  0.796
## ChildAge                    0.919  0.988
## ParentsSeparated            1.054  1.698
## Parent1_Education_level_num 0.895  1.291
## PHQ_Total                   1.002  1.102
## 
## Model Parameters (Exponentiated):
## Parameter                   |  IRR |   SE |       95% CI |     z |      p
## -------------------------------------------------------------------------
## (Intercept)                 | 5.02 | 1.07 | [3.30, 7.65] |  7.58 | < .001
## Group [Somatic Control]     | 0.66 | 0.09 | [0.50, 0.88] | -2.89 | 0.004 
## Group [Healthy Control]     | 0.74 | 0.09 | [0.58, 0.94] | -2.41 | 0.016 
## ChildGender                 | 0.65 | 0.07 | [0.53, 0.80] | -4.14 | < .001
## ChildAge                    | 0.95 | 0.02 | [0.92, 0.99] | -2.67 | 0.008 
## ParentsSeparated            | 1.34 | 0.16 | [1.05, 1.70] |  2.40 | 0.016 
## Parent1 Education level num | 1.08 | 0.10 | [0.90, 1.29] |  0.79 | 0.431 
## PHQ Total                   | 1.05 | 0.02 | [1.00, 1.10] |  2.10 | 0.036 
## 
## Model Performance:
## # Indices of model performance
## 
## AIC     |    AICc |     BIC | Nagelkerke's R2 |  RMSE | Sigma | Score_log | Score_spherical
## -------------------------------------------------------------------------------------------
## 924.571 | 925.449 | 954.907 |           0.333 | 2.195 | 1.000 |    -2.118 |           0.061
## 
## Interpretation:
## Children with FGIDs show higher hyperactivity/inattention symptoms compared to both control groups.
## Overall model fit remains good (Nagelkerke R² ≈ 0.33; AIC ≈ 924).
```

## Model for SDQ\_Peer Problems 1.4 —

```
 cat("\n--- Model for SDQ_Peer Problems ---\n")
# Negative binomial model with confounders
SDQ_PeerProb_nb <- glm.nb(
  SDQ_PeerProb ~ Group + ChildGender + ChildAge + ParentsSeparated +
    Parent1_Education_level_num + PHQ_Total,
  data = mydata)
summary(SDQ_PeerProb_nb)
cat("\nInterpretation: Both control groups show significantly fewer peer problems compared to FGID group.\n")
cat("Parental PHQ_Total also shows a positive association with peer difficulties.\n")

# Comparison Poisson model
SDQ_PeerProb_glm <- glm(
  SDQ_PeerProb ~ Group + ChildGender + ChildAge + ParentsSeparated +
    Parent1_Education_level_num + PHQ_Total,
  family = poisson(link = "log"),
  data = mydata)
cat("\n--- Poisson Model (Comparison) ---\n")
summary(SDQ_PeerProb_glm)
cat("\nOverdispersion confirmed → NB model preferred.\n")
```

```
## 
## --- Model for SDQ_Peer Problems ---
## 
## Call:
## glm.nb(formula = SDQ_PeerProb ~ Group + ChildGender + ChildAge + 
##     ParentsSeparated + Parent1_Education_level_num + PHQ_Total, 
##     data = mydata, init.theta = 1.993262476, link = log)
## 
## Coefficients:
##                             Estimate Std. Error z value Pr(>|z|)    
## (Intercept)                  0.17741    0.34460   0.515   0.6067    
## GroupSomatic Control        -1.18139    0.26402  -4.475 7.66e-06 ***
## GroupHealthy Control        -0.38304    0.19736  -1.941   0.0523 .  
## ChildGender                 -0.28732    0.17031  -1.687   0.0916 .  
## ChildAge                    -0.02534    0.02981  -0.850   0.3953    
## ParentsSeparated             0.03654    0.20356   0.179   0.8575    
## Parent1_Education_level_num  0.18304    0.14775   1.239   0.2154    
## PHQ_Total                    0.16916    0.03433   4.927 8.35e-07 ***
## ---
## Signif. codes:  0 '***' 0.001 '**' 0.01 '*' 0.05 '.' 0.1 ' ' 1
## 
## (Dispersion parameter for Negative Binomial(1.9933) family taken to be 1)
## 
##     Null deviance: 302.78  on 214  degrees of freedom
## Residual deviance: 223.29  on 207  degrees of freedom
##   (3 observations deleted due to missingness)
## AIC: 604.56
## 
## Number of Fisher Scoring iterations: 1
## 
## 
##               Theta:  1.993 
##           Std. Err.:  0.658 
## 
##  2 x log-likelihood:  -586.556 
## 
## Interpretation: Both control groups show significantly fewer peer problems compared to FGID group.
## Parental PHQ_Total also shows a positive association with peer difficulties.
## 
## --- Poisson Model (Comparison) ---
## 
## Call:
## glm(formula = SDQ_PeerProb ~ Group + ChildGender + ChildAge + 
##     ParentsSeparated + Parent1_Education_level_num + PHQ_Total, 
##     family = poisson(link = "log"), data = mydata)
## 
## Coefficients:
##                             Estimate Std. Error z value Pr(>|z|)    
## (Intercept)                  0.23526    0.25610   0.919  0.35829    
## GroupSomatic Control        -1.20327    0.22246  -5.409 6.34e-08 ***
## GroupHealthy Control        -0.42944    0.14812  -2.899  0.00374 ** 
## ChildGender                 -0.27063    0.12804  -2.114  0.03455 *  
## ChildAge                    -0.02144    0.02219  -0.966  0.33401    
## ParentsSeparated             0.02182    0.14953   0.146  0.88398    
## Parent1_Education_level_num  0.15007    0.10949   1.371  0.17051    
## PHQ_Total                    0.16087    0.02224   7.233 4.74e-13 ***
## ---
## Signif. codes:  0 '***' 0.001 '**' 0.01 '*' 0.05 '.' 0.1 ' ' 1
## 
## (Dispersion parameter for poisson family taken to be 1)
## 
##     Null deviance: 459.07  on 214  degrees of freedom
## Residual deviance: 327.32  on 207  degrees of freedom
##   (3 observations deleted due to missingness)
## AIC: 623.56
## 
## Number of Fisher Scoring iterations: 6
## 
## 
## Overdispersion confirmed → NB model preferred.
```

### Residuals, Outliers and Multicollinearity

```
cat("\n--- Multicollinearity Check (VIF) ---\n")
print(vif(SDQ_PeerProb_nb))

# Residuals and outliers
pred <- predict(SDQ_PeerProb_nb, type = "response")
resid_dev <- residuals(SDQ_PeerProb_nb, type = "deviance")
resid_sd <- sd(resid_dev)
upper <- mean(resid_dev) + 2 * resid_sd
lower <- mean(resid_dev) - 2 * resid_sd
outliers <- which(resid_dev > upper | resid_dev < lower)

cat("Number of detected outliers:", length(outliers), "\n")
if (length(outliers) > 0) {
  outlier_data <- data.frame(
    ID = mydata$ID_all[outliers],
    Residuals = resid_dev[outliers],
    Predicted = pred[outliers])
  print(outlier_data)}

# Visualization
ggplot(data.frame(predicted = pred, residuals = resid_dev), aes(x = predicted, y = residuals)) +
  geom_point(alpha = 0.7) +
  geom_hline(yintercept = 0, color = "red", linewidth = 0.5) +
  geom_point(data = subset(data.frame(predicted = pred, residuals = resid_dev),
                           residuals %in% resid_dev[outliers]),
             color = "red", size = 2) +
  theme_minimal() +
  labs(x = "Predicted values", y = "Deviance residuals",
       title = "Residuals vs Predicted (SDQ Peer Problems Model)")
```

```
# Refit model without outliers
mydata_PeerProb_clean <- mydata[-outliers, ]
SDQ_PeerProb_nb_clean <- glm.nb(
  SDQ_PeerProb ~ Group + ChildGender + ChildAge + ParentsSeparated +
    Parent1_Education_level_num + PHQ_Total,
  data = mydata_PeerProb_clean)
cat("\n--- Model without Outliers ---\n")
summary(SDQ_PeerProb_nb_clean)

# AIC comparison
aic_old <- AIC(SDQ_PeerProb_nb)
aic_new <- AIC(SDQ_PeerProb_nb_clean)
cat("\nAIC (original):", round(aic_old, 2), "| AIC (without outliers):", round(aic_new, 2), "\n")
if (aic_new < aic_old) {
  cat("→ Slight improvement after removing outliers; interpretation unchanged.\n")
} else {
  cat("→ No relevant change after removing outliers.\n")}
```

```
## 
## --- Multicollinearity Check (VIF) ---
##                                 GVIF Df GVIF^(1/(2*Df))
## Group                       1.239935  2        1.055236
## ChildGender                 1.015460  1        1.007701
## ChildAge                    1.043841  1        1.021686
## ParentsSeparated            1.106027  1        1.051678
## Parent1_Education_level_num 1.082150  1        1.040264
## PHQ_Total                   1.109848  1        1.053493
## Number of detected outliers: 8 
##      ID Residuals Predicted
## 57   54  1.718321 1.2957452
## 94   91  1.897694 0.2591165
## 97   94  1.803032 0.2908435
## 100  97  2.272370 0.5967926
## 118 115  2.065602 0.4476383
## 123 120  1.711283 0.3246417
## 351 174  2.067425 1.5703946
## 370 190  1.991981 0.7590531
## 
## --- Model without Outliers ---
## 
## Call:
## glm.nb(formula = SDQ_PeerProb ~ Group + ChildGender + ChildAge + 
##     ParentsSeparated + Parent1_Education_level_num + PHQ_Total, 
##     data = mydata_PeerProb_clean, init.theta = 2.012157104, link = log)
## 
## Coefficients:
##                             Estimate Std. Error z value Pr(>|z|)    
## (Intercept)                  0.12866    0.34878   0.369   0.7122    
## GroupSomatic Control        -1.26868    0.27961  -4.537 5.70e-06 ***
## GroupHealthy Control        -0.35494    0.20025  -1.772   0.0763 .  
## ChildGender                 -0.31352    0.17423  -1.799   0.0720 .  
## ChildAge                    -0.02683    0.03029  -0.886   0.3757    
## ParentsSeparated            -0.02382    0.20740  -0.115   0.9086    
## Parent1_Education_level_num  0.21433    0.14902   1.438   0.1504    
## PHQ_Total                    0.18194    0.03453   5.270 1.37e-07 ***
## ---
## Signif. codes:  0 '***' 0.001 '**' 0.01 '*' 0.05 '.' 0.1 ' ' 1
## 
## (Dispersion parameter for Negative Binomial(2.0122) family taken to be 1)
## 
##     Null deviance: 295.29  on 206  degrees of freedom
## Residual deviance: 211.79  on 199  degrees of freedom
##   (3 observations deleted due to missingness)
## AIC: 580.03
## 
## Number of Fisher Scoring iterations: 1
## 
## 
##               Theta:  2.012 
##           Std. Err.:  0.667 
## 
##  2 x log-likelihood:  -562.027 
## 
## AIC (original): 604.56 | AIC (without outliers): 580.03 
## → Slight improvement after removing outliers; interpretation unchanged.
```

### Model with pooled imputed data

```
cat("\n--- Pooled Imputed Data Model ---\n")
SDQ_PeerProb_nb_imp_pool <- with(
  imp_SDQ,
  glm.nb(SDQ_PeerProb ~ Group + ChildGender + ChildAge + ParentsSeparated +
           Parent1_Education_level + PHQ_Total))
summary(pool(SDQ_PeerProb_nb_imp_pool))

cat("\n--- Model on Single Imputed Dataset ---\n")
SDQ_PeerProb_nb_imp <- glm.nb(
  SDQ_PeerProb ~ Group + ChildGender + ChildAge + ParentsSeparated +
    Parent1_Education_level + PHQ_Total,
  data = mydata_SDQ_impute)
summary(SDQ_PeerProb_nb_imp)
cat("\nInterpretation: Imputed models confirm robustness; no changes in interpretation.\n")
```

```
## 
## --- Pooled Imputed Data Model ---
```

```
## 
## --- Model on Single Imputed Dataset ---
## 
## Call:
## glm.nb(formula = SDQ_PeerProb ~ Group + ChildGender + ChildAge + 
##     ParentsSeparated + Parent1_Education_level + PHQ_Total, data = mydata_SDQ_impute, 
##     init.theta = 2.022229256, link = log)
## 
## Coefficients:
##                           Estimate Std. Error z value Pr(>|z|)    
## (Intercept)                0.49180    0.28974   1.697   0.0896 .  
## GroupSomatic Control      -1.15417    0.26223  -4.401 1.08e-05 ***
## GroupHealthy Control      -0.35607    0.19686  -1.809   0.0705 .  
## ChildGender               -0.30596    0.16882  -1.812   0.0699 .  
## ChildAge                  -0.02844    0.02963  -0.960   0.3371    
## ParentsSeparated           0.04146    0.20365   0.204   0.8387    
## Parent1_Education_level.L  0.13779    0.27786   0.496   0.6200    
## Parent1_Education_level.Q -0.08721    0.19861  -0.439   0.6606    
## PHQ_Total                  0.17168    0.03418   5.022 5.10e-07 ***
## ---
## Signif. codes:  0 '***' 0.001 '**' 0.01 '*' 0.05 '.' 0.1 ' ' 1
## 
## (Dispersion parameter for Negative Binomial(2.0222) family taken to be 1)
## 
##     Null deviance: 305.99  on 217  degrees of freedom
## Residual deviance: 226.34  on 209  degrees of freedom
## AIC: 613.6
## 
## Number of Fisher Scoring iterations: 1
## 
## 
##               Theta:  2.022 
##           Std. Err.:  0.666 
## 
##  2 x log-likelihood:  -593.603 
## 
## Interpretation: Imputed models confirm robustness; no changes in interpretation.
```

### Evaluate nb-Model and calculate effect sizes

```
cat("\n--- Model Evaluation and Effect Sizes (SDQ Peer Problems) ---\n")
summary(SDQ_PeerProb_nb)

cat("\nLog-scale coefficients:\n")
print(round(coef(SDQ_PeerProb_nb), 3))
cat("\nExponentiated coefficients (Rate Ratios):\n")
print(round(exp(coef(SDQ_PeerProb_nb)), 3))
cat("\nExponentiated 95% Confidence Intervals:\n")
exp_confint <- exp(suppressMessages(confint(SDQ_PeerProb_nb)))
print(round(exp_confint, 3))

cat("\nModel Parameters (Exponentiated):\n")
print(model_parameters(SDQ_PeerProb_nb, exponentiate = TRUE))
cat("\nModel Performance:\n")
print(performance(SDQ_PeerProb_nb))

cat("\nInterpretation:\n")
cat("Children with FGIDs report higher peer problems than both control groups.\n")
cat("Parental PHQ_Total predicts greater peer issues, while other covariates show minor effects.\n")
cat("Model fit: Nagelkerke R² ≈ 0.41, AIC ≈ 605 → good model quality.\n")
```

```
## 
## --- Model Evaluation and Effect Sizes (SDQ Peer Problems) ---
## 
## Call:
## glm.nb(formula = SDQ_PeerProb ~ Group + ChildGender + ChildAge + 
##     ParentsSeparated + Parent1_Education_level_num + PHQ_Total, 
##     data = mydata, init.theta = 1.993262476, link = log)
## 
## Coefficients:
##                             Estimate Std. Error z value Pr(>|z|)    
## (Intercept)                  0.17741    0.34460   0.515   0.6067    
## GroupSomatic Control        -1.18139    0.26402  -4.475 7.66e-06 ***
## GroupHealthy Control        -0.38304    0.19736  -1.941   0.0523 .  
## ChildGender                 -0.28732    0.17031  -1.687   0.0916 .  
## ChildAge                    -0.02534    0.02981  -0.850   0.3953    
## ParentsSeparated             0.03654    0.20356   0.179   0.8575    
## Parent1_Education_level_num  0.18304    0.14775   1.239   0.2154    
## PHQ_Total                    0.16916    0.03433   4.927 8.35e-07 ***
## ---
## Signif. codes:  0 '***' 0.001 '**' 0.01 '*' 0.05 '.' 0.1 ' ' 1
## 
## (Dispersion parameter for Negative Binomial(1.9933) family taken to be 1)
## 
##     Null deviance: 302.78  on 214  degrees of freedom
## Residual deviance: 223.29  on 207  degrees of freedom
##   (3 observations deleted due to missingness)
## AIC: 604.56
## 
## Number of Fisher Scoring iterations: 1
## 
## 
##               Theta:  1.993 
##           Std. Err.:  0.658 
## 
##  2 x log-likelihood:  -586.556 
## 
## Log-scale coefficients:
##                 (Intercept)        GroupSomatic Control 
##                       0.177                      -1.181 
##        GroupHealthy Control                 ChildGender 
##                      -0.383                      -0.287 
##                    ChildAge            ParentsSeparated 
##                      -0.025                       0.037 
## Parent1_Education_level_num                   PHQ_Total 
##                       0.183                       0.169 
## 
## Exponentiated coefficients (Rate Ratios):
##                 (Intercept)        GroupSomatic Control 
##                       1.194                       0.307 
##        GroupHealthy Control                 ChildGender 
##                       0.682                       0.750 
##                    ChildAge            ParentsSeparated 
##                       0.975                       1.037 
## Parent1_Education_level_num                   PHQ_Total 
##                       1.201                       1.184 
## 
## Exponentiated 95% Confidence Intervals:
##                             2.5 % 97.5 %
## (Intercept)                 0.620  2.289
## GroupSomatic Control        0.181  0.509
## GroupHealthy Control        0.462  1.007
## ChildGender                 0.535  1.049
## ChildAge                    0.919  1.034
## ParentsSeparated            0.689  1.552
## Parent1_Education_level_num 0.897  1.606
## PHQ_Total                   1.106  1.271
## 
## Model Parameters (Exponentiated):
## Parameter                   |  IRR |   SE |       95% CI |     z |      p
## -------------------------------------------------------------------------
## (Intercept)                 | 1.19 | 0.41 | [0.62, 2.29] |  0.51 | 0.607 
## Group [Somatic Control]     | 0.31 | 0.08 | [0.18, 0.51] | -4.47 | < .001
## Group [Healthy Control]     | 0.68 | 0.13 | [0.46, 1.01] | -1.94 | 0.052 
## ChildGender                 | 0.75 | 0.13 | [0.53, 1.05] | -1.69 | 0.092 
## ChildAge                    | 0.97 | 0.03 | [0.92, 1.03] | -0.85 | 0.395 
## ParentsSeparated            | 1.04 | 0.21 | [0.69, 1.55] |  0.18 | 0.858 
## Parent1 Education level num | 1.20 | 0.18 | [0.90, 1.61] |  1.24 | 0.215 
## PHQ Total                   | 1.18 | 0.04 | [1.11, 1.27] |  4.93 | < .001
## 
## Model Performance:
## # Indices of model performance
## 
## AIC     |    AICc |     BIC | Nagelkerke's R2 |  RMSE | Sigma | Score_log | Score_spherical
## -------------------------------------------------------------------------------------------
## 604.556 | 605.434 | 634.891 |           0.409 | 1.367 | 1.000 |    -1.367 |           0.057
## 
## Interpretation:
## Children with FGIDs report higher peer problems than both control groups.
## Parental PHQ_Total predicts greater peer issues, while other covariates show minor effects.
## Model fit: Nagelkerke R² ≈ 0.41, AIC ≈ 605 → good model quality.
```

## Model for SDQ\_Prosocial Behavior 1.5 —

```
cat("\n--- Model for SDQ_Prosocial (Prosocial Behaviour) ---\n")
# Negative binomial model with confounders
SDQ_Prosocial_nb <- glm.nb(
  SDQ_Prosocial ~ Group + ChildGender + ChildAge + ParentsSeparated +
    Parent1_Education_level_num + PHQ_Total,
  data = mydata)
summary(SDQ_Prosocial_nb)
cat("\nInterpretation: Somatic Control group shows significantly higher prosocial scores.\n")
cat("No other covariates significantly influence prosocial behaviour.\n")
cat("Prosocial scores are left-skewed but showed overdispersion and ceiling effects; negative binomial models were therefore retained for consistency with other SDQ outcomes.\n")
```

```
## 
## --- Model for SDQ_Prosocial (Prosocial Behaviour) ---
## 
## Call:
## glm.nb(formula = SDQ_Prosocial ~ Group + ChildGender + ChildAge + 
##     ParentsSeparated + Parent1_Education_level_num + PHQ_Total, 
##     data = mydata, init.theta = 381705.018, link = log)
## 
## Coefficients:
##                              Estimate Std. Error z value Pr(>|z|)    
## (Intercept)                  1.997579   0.106079  18.831  < 2e-16 ***
## GroupSomatic Control         0.233729   0.067827   3.446 0.000569 ***
## GroupHealthy Control         0.084016   0.064068   1.311 0.189738    
## ChildGender                  0.082833   0.047973   1.727 0.084226 .  
## ChildAge                     0.013357   0.008491   1.573 0.115729    
## ParentsSeparated            -0.065360   0.060981  -1.072 0.283803    
## Parent1_Education_level_num -0.070259   0.045753  -1.536 0.124632    
## PHQ_Total                   -0.018030   0.012431  -1.450 0.146953    
## ---
## Signif. codes:  0 '***' 0.001 '**' 0.01 '*' 0.05 '.' 0.1 ' ' 1
## 
## (Dispersion parameter for Negative Binomial(381705) family taken to be 1)
## 
##     Null deviance: 128.565  on 214  degrees of freedom
## Residual deviance:  92.646  on 207  degrees of freedom
##   (3 observations deleted due to missingness)
## AIC: 951.51
## 
## Number of Fisher Scoring iterations: 1
## 
## 
##               Theta:  381705 
##           Std. Err.:  3743151 
## Warning while fitting theta: iteration limit reached 
## 
##  2 x log-likelihood:  -933.511 
## 
## Interpretation: Somatic Control group shows significantly higher prosocial scores.
## No other covariates significantly influence prosocial behaviour.
## Prosocial scores are left-skewed but showed overdispersion and ceiling effects; negative binomial models were therefore retained for consistency with other SDQ outcomes.
```

### Residuals, Outliers and Multicollinearity

```
cat("\n--- Multicollinearity Check (VIF) ---\n")
print(vif(SDQ_Prosocial_nb))

# Residuals and outliers
pred <- predict(SDQ_Prosocial_nb, type = "response")
resid_dev <- residuals(SDQ_Prosocial_nb, type = "deviance")
resid_sd <- sd(resid_dev)
upper <- mean(resid_dev) + 2 * resid_sd
lower <- mean(resid_dev) - 2 * resid_sd
outliers <- which(resid_dev > upper | resid_dev < lower)

cat("Number of detected outliers:", length(outliers), "\n")
if (length(outliers) > 0) {
  outlier_data <- data.frame(
    ID = mydata$ID_all[outliers],
    Residuals = resid_dev[outliers],
    Predicted = pred[outliers])
  print(outlier_data)}

# Visualization
ggplot(data.frame(predicted = pred, residuals = resid_dev),
       aes(x = predicted, y = residuals)) +
  geom_point(alpha = 0.7) +
  geom_hline(yintercept = 0, color = "red", linewidth = 0.5) +
  geom_point(data = subset(data.frame(predicted = pred, residuals = resid_dev),
                           residuals %in% resid_dev[outliers]),
             color = "red", size = 2) +
  theme_minimal() +
  labs(x = "Predicted values", y = "Deviance residuals",
       title = "Residuals vs Predicted (SDQ Prosocial Model)")
```

```
# Refit model without outliers
mydata_Prosocial_clean <- mydata[-outliers, ]
SDQ_Prosocial_nb_clean <- glm.nb(
  SDQ_Prosocial ~ Group + ChildGender + ChildAge + ParentsSeparated +
    Parent1_Education_level_num + PHQ_Total,
  data = mydata_Prosocial_clean)
cat("\n--- Model without Outliers ---\n")
summary(SDQ_Prosocial_nb_clean)

# AIC comparison
aic_old <- AIC(SDQ_Prosocial_nb)
aic_new <- AIC(SDQ_Prosocial_nb_clean)
cat("\nAIC (original):", round(aic_old, 2), "| AIC (without outliers):", round(aic_new, 2), "\n")
if (aic_new < aic_old) {
  cat("→ Model fit improved after removing outliers; interpretation unchanged.\n")
} else {
  cat("→ No relevant change after removing outliers.\n")}
```

```
## 
## --- Multicollinearity Check (VIF) ---
##                                 GVIF Df GVIF^(1/(2*Df))
## Group                       1.224546  2        1.051947
## ChildGender                 1.013960  1        1.006956
## ChildAge                    1.075643  1        1.037132
## ParentsSeparated            1.054204  1        1.026744
## Parent1_Education_level_num 1.063837  1        1.031425
## PHQ_Total                   1.132089  1        1.063997
## Number of detected outliers: 8 
##      ID Residuals Predicted
## 6     6 -1.409958  6.145161
## 17   17 -1.352304  7.345612
## 28   26 -1.509605  6.423541
## 29   27 -1.788419  5.690564
## 33   31  1.307822  6.413650
## 57   54 -3.807383  7.248152
## 364 185 -1.597565  8.096853
## 382 199 -3.194002  8.205724
## 
## --- Model without Outliers ---
## 
## Call:
## glm.nb(formula = SDQ_Prosocial ~ Group + ChildGender + ChildAge + 
##     ParentsSeparated + Parent1_Education_level_num + PHQ_Total, 
##     data = mydata_Prosocial_clean, init.theta = 394669.8684, 
##     link = log)
## 
## Coefficients:
##                              Estimate Std. Error z value Pr(>|z|)    
## (Intercept)                  2.002588   0.108798  18.407  < 2e-16 ***
## GroupSomatic Control         0.233856   0.069813   3.350 0.000809 ***
## GroupHealthy Control         0.083125   0.066335   1.253 0.210170    
## ChildGender                  0.082291   0.048753   1.688 0.091427 .  
## ChildAge                     0.012139   0.008569   1.417 0.156610    
## ParentsSeparated            -0.071287   0.062074  -1.148 0.250790    
## Parent1_Education_level_num -0.065613   0.046068  -1.424 0.154370    
## PHQ_Total                   -0.018191   0.012662  -1.437 0.150834    
## ---
## Signif. codes:  0 '***' 0.001 '**' 0.01 '*' 0.05 '.' 0.1 ' ' 1
## 
## (Dispersion parameter for Negative Binomial(394669.9) family taken to be 1)
## 
##     Null deviance: 120.797  on 206  degrees of freedom
## Residual deviance:  87.191  on 199  degrees of freedom
##   (3 observations deleted due to missingness)
## AIC: 915.85
## 
## Number of Fisher Scoring iterations: 1
## 
## 
##               Theta:  394670 
##           Std. Err.:  3975482 
## Warning while fitting theta: iteration limit reached 
## 
##  2 x log-likelihood:  -897.849 
## 
## AIC (original): 951.51 | AIC (without outliers): 915.85 
## → Model fit improved after removing outliers; interpretation unchanged.
```

### Model with pooled imputed data

```
cat("\n--- Pooled Imputed Data Model ---\n")
SDQ_Prosocial_nb_imp_pool <- with(
  imp_SDQ,
  glm.nb(SDQ_Prosocial ~ Group + ChildGender + ChildAge + ParentsSeparated +
           Parent1_Education_level + PHQ_Total))
summary(pool(SDQ_Prosocial_nb_imp_pool))

cat("\n--- Model on Single Imputed Dataset ---\n")
SDQ_Prosocial_nb_imp <- glm.nb(
  SDQ_Prosocial ~ Group + ChildGender + ChildAge + ParentsSeparated +
    Parent1_Education_level + PHQ_Total,
  data = mydata_SDQ_impute)
summary(SDQ_Prosocial_nb_imp)
cat("\nInterpretation: Imputed and original models show same pattern; results robust.\n")
```

```
## 
## --- Pooled Imputed Data Model ---
```

```
## 
## --- Model on Single Imputed Dataset ---
## 
## Call:
## glm.nb(formula = SDQ_Prosocial ~ Group + ChildGender + ChildAge + 
##     ParentsSeparated + Parent1_Education_level + PHQ_Total, data = mydata_SDQ_impute, 
##     init.theta = 391327.8733, link = log)
## 
## Coefficients:
##                            Estimate Std. Error z value Pr(>|z|)    
## (Intercept)                1.817456   0.096825  18.771  < 2e-16 ***
## GroupSomatic Control       0.215578   0.066957   3.220  0.00128 ** 
## GroupHealthy Control       0.080550   0.063256   1.273  0.20287    
## ChildGender                0.083843   0.047741   1.756  0.07905 .  
## ChildAge                   0.012496   0.008446   1.479  0.13902    
## ParentsSeparated          -0.064793   0.060822  -1.065  0.28674    
## Parent1_Education_level.L -0.237453   0.103649  -2.291  0.02197 *  
## Parent1_Education_level.Q -0.124149   0.069664  -1.782  0.07473 .  
## PHQ_Total                 -0.019373   0.012398  -1.563  0.11817    
## ---
## Signif. codes:  0 '***' 0.001 '**' 0.01 '*' 0.05 '.' 0.1 ' ' 1
## 
## (Dispersion parameter for Negative Binomial(391327.9) family taken to be 1)
## 
##     Null deviance: 130.757  on 217  degrees of freedom
## Residual deviance:  91.739  on 209  degrees of freedom
## AIC: 964.4
## 
## Number of Fisher Scoring iterations: 1
## 
## 
##               Theta:  391328 
##           Std. Err.:  3842220 
## Warning while fitting theta: iteration limit reached 
## 
##  2 x log-likelihood:  -944.398 
## 
## Interpretation: Imputed and original models show same pattern; results robust.
```

### Evaluate nb-Model and calculate effect sizes

```
cat("\n--- Model Evaluation and Effect Sizes (SDQ Prosocial) ---\n")
summary(SDQ_Prosocial_nb)

cat("\nLog-scale coefficients:\n")
print(round(coef(SDQ_Prosocial_nb), 3))
cat("\nExponentiated coefficients (Rate Ratios):\n")
print(round(exp(coef(SDQ_Prosocial_nb)), 3))
cat("\nExponentiated 95% Confidence Intervals:\n")
exp_confint <- exp(suppressMessages(confint(SDQ_Prosocial_nb)))
print(round(exp_confint, 3))

cat("\nModel Parameters (Exponentiated):\n")
print(model_parameters(SDQ_Prosocial_nb, exponentiate = TRUE))
cat("\nModel Performance:\n")
print(performance(SDQ_Prosocial_nb))

cat("\nInterpretation:\n")
cat("Children in the somatic control group show slightly higher prosocial behaviour scores.\n")
cat("Other factors (age, gender, parental PHQ) show no significant effects.\n")
cat("Model fit is moderate (Nagelkerke R² ≈ 0.34, AIC ≈ 952).\n")
```

```
## 
## --- Model Evaluation and Effect Sizes (SDQ Prosocial) ---
## 
## Call:
## glm.nb(formula = SDQ_Prosocial ~ Group + ChildGender + ChildAge + 
##     ParentsSeparated + Parent1_Education_level_num + PHQ_Total, 
##     data = mydata, init.theta = 381705.018, link = log)
## 
## Coefficients:
##                              Estimate Std. Error z value Pr(>|z|)    
## (Intercept)                  1.997579   0.106079  18.831  < 2e-16 ***
## GroupSomatic Control         0.233729   0.067827   3.446 0.000569 ***
## GroupHealthy Control         0.084016   0.064068   1.311 0.189738    
## ChildGender                  0.082833   0.047973   1.727 0.084226 .  
## ChildAge                     0.013357   0.008491   1.573 0.115729    
## ParentsSeparated            -0.065360   0.060981  -1.072 0.283803    
## Parent1_Education_level_num -0.070259   0.045753  -1.536 0.124632    
## PHQ_Total                   -0.018030   0.012431  -1.450 0.146953    
## ---
## Signif. codes:  0 '***' 0.001 '**' 0.01 '*' 0.05 '.' 0.1 ' ' 1
## 
## (Dispersion parameter for Negative Binomial(381705) family taken to be 1)
## 
##     Null deviance: 128.565  on 214  degrees of freedom
## Residual deviance:  92.646  on 207  degrees of freedom
##   (3 observations deleted due to missingness)
## AIC: 951.51
## 
## Number of Fisher Scoring iterations: 1
## 
## 
##               Theta:  381705 
##           Std. Err.:  3743151 
## Warning while fitting theta: iteration limit reached 
## 
##  2 x log-likelihood:  -933.511 
## 
## Log-scale coefficients:
##                 (Intercept)        GroupSomatic Control 
##                       1.998                       0.234 
##        GroupHealthy Control                 ChildGender 
##                       0.084                       0.083 
##                    ChildAge            ParentsSeparated 
##                       0.013                      -0.065 
## Parent1_Education_level_num                   PHQ_Total 
##                      -0.070                      -0.018 
## 
## Exponentiated coefficients (Rate Ratios):
##                 (Intercept)        GroupSomatic Control 
##                       7.371                       1.263 
##        GroupHealthy Control                 ChildGender 
##                       1.088                       1.086 
##                    ChildAge            ParentsSeparated 
##                       1.013                       0.937 
## Parent1_Education_level_num                   PHQ_Total 
##                       0.932                       0.982 
## 
## Exponentiated 95% Confidence Intervals:
##                             2.5 % 97.5 %
## (Intercept)                 5.981  9.065
## GroupSomatic Control        1.106  1.443
## GroupHealthy Control        0.960  1.234
## ChildGender                 0.989  1.193
## ChildAge                    0.997  1.030
## ParentsSeparated            0.830  1.054
## Parent1_Education_level_num 0.851  1.019
## PHQ_Total                   0.958  1.006
## 
## Model Parameters (Exponentiated):
## Parameter                   |  IRR |       SE |       95% CI |     z |      p
## -----------------------------------------------------------------------------
## (Intercept)                 | 7.37 |     0.78 | [5.98, 9.07] | 18.83 | < .001
## Group [Somatic Control]     | 1.26 |     0.09 | [1.11, 1.44] |  3.45 | < .001
## Group [Healthy Control]     | 1.09 |     0.07 | [0.96, 1.23] |  1.31 | 0.190 
## ChildGender                 | 1.09 |     0.05 | [0.99, 1.19] |  1.73 | 0.084 
## ChildAge                    | 1.01 | 8.61e-03 | [1.00, 1.03] |  1.57 | 0.116 
## ParentsSeparated            | 0.94 |     0.06 | [0.83, 1.05] | -1.07 | 0.284 
## Parent1 Education level num | 0.93 |     0.04 | [0.85, 1.02] | -1.54 | 0.125 
## PHQ Total                   | 0.98 |     0.01 | [0.96, 1.01] | -1.45 | 0.147 
## 
## Model Performance:
## # Indices of model performance
## 
## AIC     |    AICc |     BIC | Nagelkerke's R2 |  RMSE | Sigma | Score_log | Score_spherical
## -------------------------------------------------------------------------------------------
## 951.511 | 952.389 | 981.847 |           0.342 | 1.651 | 1.000 |      -Inf |           0.066
## 
## Interpretation:
## Children in the somatic control group show slightly higher prosocial behaviour scores.
## Other factors (age, gender, parental PHQ) show no significant effects.
## Model fit is moderate (Nagelkerke R² ≈ 0.34, AIC ≈ 952).
```

# Forest plot SDQ subscores —

```
SDQ_forestplot <- data.frame(
  Outcome = rep(c("Total difficulties score", "Emotional problems", "Conduct problems",
                  "Hyperactivity", "Peer problems", "Prosocial behavior"), each = 2),
  Group   = rep(c("Healthy Control", "Somatic Control"), times = 6),
  IRR     = c(0.61, 0.49, 0.36, 0.42, 0.72, 0.46, 0.74, 0.66, 0.68, 0.31, 1.09, 1.26),
  CI_low  = c(0.49, 0.38, 0.27, 0.30, 0.50, 0.30, 0.58, 0.50, 0.46, 0.18, 0.96, 1.11),
  CI_high = c(0.75, 0.62, 0.49, 0.58, 1.02, 0.68, 0.94, 0.88, 1.01, 0.51, 1.23, 1.44))

# --- Reorder outcomes ---
SDQ_forestplot <- SDQ_forestplot %>%
  mutate(Outcome = factor(Outcome, levels = rev(c(
    "Total difficulties score",
    "Emotional problems",
    "Conduct problems",
    "Peer problems",
    "Hyperactivity",
    "Prosocial behavior"))))
group_colors <- c("Somatic Control" = "#E7B800",  
                  "Healthy Control" = "#1B9E77")

# Forest plot SDQ subscores – optimized spacing for readability
Fig_Forest <- ggplot(SDQ_forestplot, aes(x = IRR, y = Outcome, color = Group)) +
  geom_errorbarh(aes(xmin = CI_low, xmax = CI_high),
                 position = position_dodge(width = 0.5),
                 height = 0.15, linewidth = 0.9) +
  geom_point(position = position_dodge(width = 0.5),
             size = 3.8, show.legend = FALSE) +
  geom_text(aes(x = CI_high + 0.05,
                label = sprintf("%.2f [%.2f–%.2f]", IRR, CI_low, CI_high)),
            position = position_dodge(width = 0.5),
            hjust = 0, vjust = 0.4, size = 3.4,
            show.legend = FALSE) +
  geom_vline(xintercept = 1, linetype = "dashed", color = "grey70", linewidth = 0.4,alpha = 0.6) +
  geom_hline(yintercept = 5.5, color = "grey70", linetype = "solid", linewidth = 0.6) +
  scale_x_continuous(limits = c(0.15, 1.65),
                     breaks = seq(0.2, 1.4, 0.2),
                     expand = expansion(mult = c(0, 0.25))) +
  scale_color_manual(values = group_colors) +
  labs(x = "Incidence Rate Ratio (IRR; point estimate with 95% CI)", y = "", color = "Group") +
  theme_minimal(base_size = 13) +
  theme(
    legend.position = "top",
    legend.text = element_text(size = 11),
    panel.grid.major.y = element_blank(),
    panel.grid.minor = element_blank(),
    panel.background = element_rect(fill = "white", color = NA),
    plot.background = element_rect(fill = "white", color = NA),
    axis.text = element_text(size = 11, color = "black"),
    axis.title.x = element_text(size = 13, face = "bold"),
    strip.text = element_text(face = "bold", size = 12),
    plot.margin = margin(5, 60, 5, 5))
print(Fig_Forest)
```

```
# Forest plot SDQ subscores ------------------------------------------------
SDQ_forestplot <- data.frame(
  Outcome = rep(c("Total Difficulties",
                  "Emotional Problems",
                  "Conduct Problems",
                  "Hyperactivity",
                  "Peer Problems",
                  "Prosocial Behaviour"), each = 2),
  Group   = rep(c("Healthy Control", "Somatic Control"), times = 6),
  IRR     = c(0.61, 0.49, 0.36, 0.42, 0.72, 0.46, 0.74, 0.66, 0.68, 0.31, 1.09, 1.26),
  CI_low  = c(0.49, 0.38, 0.27, 0.30, 0.50, 0.30, 0.58, 0.50, 0.46, 0.18, 0.96, 1.11),
  CI_high = c(0.75, 0.62, 0.49, 0.58, 1.02, 0.68, 0.94, 0.88, 1.01, 0.51, 1.23, 1.44))

# --- Reorder outcomes (same structure as before) ---
SDQ_forestplot <- SDQ_forestplot %>%
  mutate(Outcome = factor(Outcome, levels = rev(c(
    "Total Difficulties",
    "Emotional Problems",
    "Conduct Problems",
    "Peer Problems",
    "Hyperactivity",
    "Prosocial Behaviour"))))
group_colors <- c("Somatic Control" = "#E7B800",
                  "Healthy Control" = "#1B9E77")

# Forest plot SDQ subscores – optimized spacing for readability
Fig_Forest <- ggplot(SDQ_forestplot, aes(x = IRR, y = Outcome, color = Group)) +
  geom_errorbarh(aes(xmin = CI_low, xmax = CI_high),
                 position = position_dodge(width = 0.5),
                 height = 0.15, linewidth = 0.9) +
  geom_point(position = position_dodge(width = 0.5),
             size = 3.8, show.legend = FALSE) +
  geom_text(aes(x = CI_high + 0.05,
                label = sprintf("%.2f [%.2f–%.2f]", IRR, CI_low, CI_high)),
            position = position_dodge(width = 0.5),
            hjust = 0, vjust = 0.4, size = 3.4,
            show.legend = FALSE) +
  geom_vline(xintercept = 1, linetype = "dashed", color = "grey70",
             linewidth = 0.4, alpha = 0.6) +
  geom_hline(yintercept = 5.5, color = "grey70", linetype = "solid", linewidth = 0.6) +
  scale_x_continuous(limits = c(0.15, 1.65),
                     breaks = seq(0.2, 1.4, 0.2),
                     expand = expansion(mult = c(0, 0.25))) +
  scale_color_manual(values = group_colors) +
  labs(x = NULL, y = NULL, color = "Group") +
  theme_minimal(base_size = 13) +
  theme(
    legend.position = "bottom",
    legend.text = element_text(size = 11),
    panel.grid.major.y = element_blank(),
    panel.grid.minor = element_blank(),
    panel.background = element_rect(fill = "white", color = NA),
    plot.background = element_rect(fill = "white", color = NA),
    axis.text.x = element_text(size = 11, color = "black"),
    axis.text.y = element_text(size = 12, color = "black", face = "bold"),
    axis.title.x = element_blank(),
    plot.margin = margin(5, 60, 5, 5))
print(Fig_Forest)
```

```
# Export final figure
ggsave("Figure2_SDQ_ForestPlot.tiff",
       Fig_Forest,
       width = 7, height = 5, dpi = 600,
       compression = "lzw",
       bg = "white")
```

# Saving the data

```
cat("\n\nAnalysis completed successfully on:", Sys.Date(), "\n")

save(mydata, file=dataSetOutput)

save(SDQ_nb, SDQ_Emotional_nb, SDQ_Conduct_nb, SDQ_Hyperact_nb, 
     SDQ_PeerProb_nb, SDQ_Prosocial_nb, 
     Fig_Box, Fig_Forest,
     file = "Final_Models_and_Figures.RData")
```

```
## 
## 
## Analysis completed successfully on: 20487
```

# Session Info

```
sessionInfo()
```

```
## R version 4.4.2 (2024-10-31)
## Platform: aarch64-apple-darwin20
## Running under: macOS Sonoma 14.6
## 
## Matrix products: default
## BLAS:   /Library/Frameworks/R.framework/Versions/4.4-arm64/Resources/lib/libRblas.0.dylib 
## LAPACK: /Library/Frameworks/R.framework/Versions/4.4-arm64/Resources/lib/libRlapack.dylib;  LAPACK version 3.12.0
## 
## locale:
## [1] en_US.UTF-8/en_US.UTF-8/en_US.UTF-8/C/en_US.UTF-8/en_US.UTF-8
## 
## time zone: Europe/Zurich
## tzcode source: internal
## 
## attached base packages:
## [1] stats     graphics  grDevices utils     datasets  methods   base     
## 
## other attached packages:
##  [1] mice_3.17.0        reshape2_1.4.4     Hmisc_5.2-2        lm.beta_1.7-2     
##  [5] janitor_2.2.1      see_0.9.0          report_0.6.0       parameters_0.24.1 
##  [9] modelbased_0.8.9   insight_1.0.1      effectsize_1.0.0   datawizard_1.0.0  
## [13] correlation_0.8.6  bayestestR_0.15.0  easystats_0.7.3    jtools_2.3.0      
## [17] QuantPsyc_1.6      MASS_7.3-61        boot_1.3-31        effsize_0.8.1     
## [21] car_3.1-3          carData_3.0-5      performance_0.13.0 pwr_1.3-0         
## [25] lmtest_0.9-40      zoo_1.8-12         lubridate_1.9.4    forcats_1.0.0     
## [29] stringr_1.5.1      purrr_1.0.2        readr_2.1.5        tibble_3.2.1      
## [33] ggplot2_3.5.1      tidyverse_2.0.0    psych_2.5.3        FSA_0.9.6         
## [37] tidyr_1.3.1        dplyr_1.1.4        tableone_0.13.2   
## 
## loaded via a namespace (and not attached):
##   [1] shape_1.4.6.1       rstudioapi_0.17.1   jsonlite_1.8.9     
##   [4] magrittr_2.0.3      dunn.test_1.3.6     jomo_2.7-6         
##   [7] TH.data_1.1-2       estimability_1.5.1  nloptr_2.1.1       
##  [10] farver_2.1.2        rmarkdown_2.29      ragg_1.3.3         
##  [13] vctrs_0.6.5         minqa_1.2.8         base64enc_0.1-3    
##  [16] htmltools_0.5.8.1   haven_2.5.4         survey_4.4-2       
##  [19] broom_1.0.7         Formula_1.2-5       mitml_0.4-5        
##  [22] sass_0.4.9          parallelly_1.42.0   bslib_0.8.0        
##  [25] htmlwidgets_1.6.4   plyr_1.8.9          sandwich_3.1-1     
##  [28] emmeans_1.10.7      cachem_1.1.0        lifecycle_1.0.4    
##  [31] iterators_1.0.14    pkgconfig_2.0.3     Matrix_1.7-1       
##  [34] R6_2.5.1            fastmap_1.2.0       future_1.34.0      
##  [37] snakecase_0.11.1    digest_0.6.37       colorspace_2.1-1   
##  [40] furrr_0.3.1         textshaping_0.4.1   labeling_0.4.3     
##  [43] fansi_1.0.6         timechange_0.3.0    abind_1.4-8        
##  [46] compiler_4.4.2      proxy_0.4-27        withr_3.0.2        
##  [49] pander_0.6.5        htmlTable_2.4.3     backports_1.5.0    
##  [52] DBI_1.2.3           pan_1.9             broom.mixed_0.2.9.6
##  [55] tools_4.4.2         foreign_0.8-87      nnet_7.3-20        
##  [58] glue_1.8.0          nlme_3.1-166        grid_4.4.2         
##  [61] checkmate_2.3.2     cluster_2.1.6       generics_0.1.3     
##  [64] gtable_0.3.6        labelled_2.13.0     tzdb_0.5.0         
##  [67] class_7.3-22        data.table_1.16.4   hms_1.1.3          
##  [70] utf8_1.2.4          foreach_1.5.2       pillar_1.9.0       
##  [73] mitools_2.4         splines_4.4.2       lattice_0.22-6     
##  [76] survival_3.7-0      tidyselect_1.2.1    knitr_1.49         
##  [79] gridExtra_2.3       xfun_0.54           stringi_1.8.4      
##  [82] yaml_2.3.10         evaluate_1.0.1      codetools_0.2-20   
##  [85] cli_3.6.5           rpart_4.1.23        systemfonts_1.1.0  
##  [88] xtable_1.8-4        munsell_0.5.1       jquerylib_0.1.4    
##  [91] Rcpp_1.0.13-1       globals_0.16.3      coda_0.19-4.1      
##  [94] parallel_4.4.2      lme4_1.1-35.5       listenv_0.9.1      
##  [97] glmnet_4.1-8        mvtnorm_1.3-3       scales_1.3.0       
## [100] e1071_1.7-16        rlang_1.1.6         multcomp_1.4-26    
## [103] mnormt_2.1.1
```
